# Proteomic Analysis of a Fraction with Intact Eyespots of *Chlamydomonas reinhardtii* and Assignment of Protein Methylation

**DOI:** 10.3389/fpls.2015.01085

**Published:** 2015-12-15

**Authors:** Nicole Eitzinger, Volker Wagner, Wolfram Weisheit, Stefan Geimer, David Boness, Georg Kreimer, Maria Mittag

**Affiliations:** ^1^Cell Biology, Department of Biology, Friedrich-Alexander-University Erlangen-NurembergErlangen, Germany; ^2^Institute of General Botany and Plant Physiology, Faculty of Biology and Pharmacy, Friedrich Schiller University JenaJena, Germany; ^3^Cell Biology and Electron Microscopy, University of BayreuthBayreuth, Germany

**Keywords:** ATP synthase, *Chlamydomonas reinhardtii*, EYE2, eyespot proteome, phototaxis, protein methylation, SOUL heme-binding protein 3

## Abstract

Flagellate green algae possess a visual system, the eyespot. In *Chlamydomonas reinhardtii* it is situated at the edge of the chloroplast and consists of two carotenoid rich lipid globule layers subtended by thylakoid membranes (TM) that are attached to both chloroplast envelope membranes and a specialized area of the plasma membrane (PM). A former analysis of an eyespot fraction identified 203 proteins. To increase the understanding of eyespot related processes, knowledge of the protein composition of the membranes in its close vicinity is desirable. Here, we present a purification procedure that allows isolation of intact eyespots. This gain in intactness goes, however, hand in hand with an increase of contaminants from other organelles. Proteomic analysis identified 742 proteins. Novel candidates include proteins for eyespot development, retina-related proteins, ion pumps, and membrane-associated proteins, calcium sensing proteins as well as kinases, phosphatases and 14-3-3 proteins. Methylation of proteins at Arg or Lys is known as an important posttranslational modification involved in, e.g., signal transduction. Here, we identify several proteins from eyespot fractions that are methylated at Arg and/or Lys. Among them is the eyespot specific SOUL3 protein that influences the size and position of the eyespot and EYE2, a protein important for its development.

## Introduction

**FIGURE 1:**
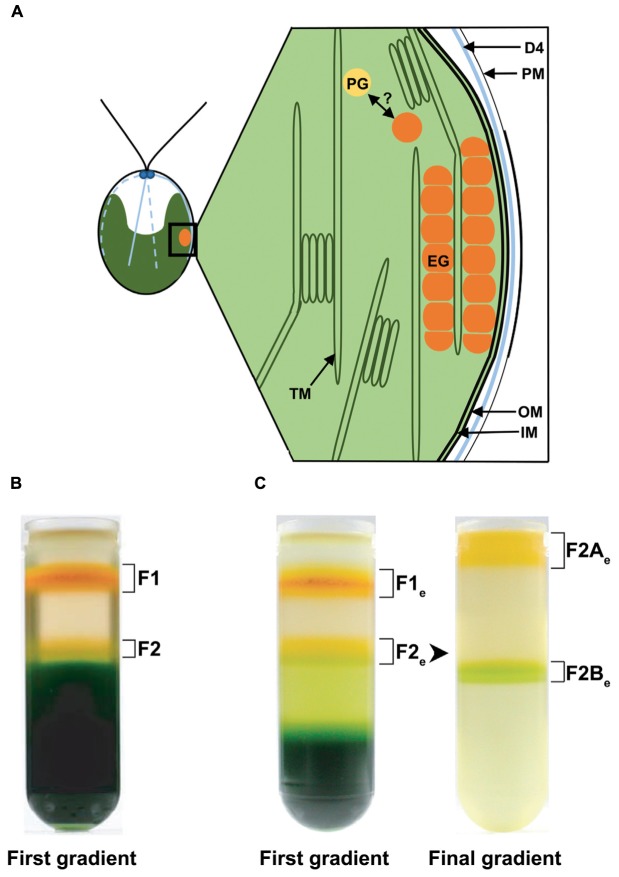
**Schematic drawing of the eyespot in *Chlamydomonas reinhardtii* and distribution of different fractions enriched in eyespots after flotation on discontinuous sucrose gradients. (A)** Schematic longitudinal section through the eyespot in the region of the four stranded microtubular root (D4), which is important for eyespot positioning. The functional eyespot consists of local specializations of different compartments. The part of the plasma membrane (PM) overlying the eyespot globules (EG) is thickened and in close association with the inner and outer chloroplast envelope (IM/OM). The EG are in contact with the thylakoid membrane (TM) and arranged in layers, the outermost being also in contact with the chloroplast envelope. The possible link between plastoglobules (PG) and EG is indicated. **(B)** Separation of the cell homogenate in the first gradient using the established eyespot isolation method by Schmidt et al. (2006). **(C)** Separation of the cell homogenate in the first gradient using the here described (see Materials and Methods) modified cell rupture method for an extended (“e”) eyespot fraction; separation of fraction 2 (F2_e_) from these gradients in fraction 2A (F2A_e_) and 2B (F2B_e_) in the final gradient. Note the increased amount of F2_e_ and the decreased amount of F1_e_ and thylakoid debris in comparison to the original cell rupture method.

Many motile algae exhibit a peculiar photo-behavior: movement toward or away from the light source depending on the light intensity and quality. This behavior is known as positive or negative phototaxis. To allow such precise movement responses, many flagellate algae of all major phylogenetic lineages have developed specialized optical devices (eyespots), which are antennae that determine the direction of incident light (Foster and Smyth, 1980; Kreimer, 1994, 2009). In some warnowiid dinoflagellates, this structure is extremely complex and called ocelloid. Its ultrastructure bears apparent resemblance to camera-type eyes of some animals and has recently been shown to be composed of different specialized cell organelles such as plastids, mitochondria, and vesicles. The ocelloid is probably homologous to simpler dinoflagellate eyespot types, most of which involve parts of the chloroplasts (Dodge, 1984; Kawai and Kreimer, 2000; Gavelis et al., 2015; Hayakawa et al., 2015). In green algae, the functional eyespot is also a composed “organelle”, involving local specializations from different subcellular compartments and highly ordered carotenoid-rich globules inside the chloroplast. It is peripherally located in the cell and readily observable by bright-field microscopy as an orange- to red-colored spot (Melkonian and Robenek, 1984; Kreimer, 2009). In *Chlamydomonas reinhardtii*, the eyespot typically consists of two highly ordered layers of such globules, each subtended by a thylakoid (see **Figure 1A** for a schematic drawing). The outermost globule layer is attached to specialized areas of both the chloroplast envelope and the adjacent PM. The globule layers modulate the light intensity reaching the photoreceptors in the PM patch as the cell rotates around its longitudinal axis during forward swimming. They function as a combined absorption screen and quarter-wave interference reflector. Thereby, the contrast at the photoreceptors is increased up to eightfold, making the whole optical system highly directional (Foster and Smyth, 1980; Kreimer and Melkonian, 1990; Harz et al., 1992; Kreimer et al., 1992). Beside its function as a sensor for light direction and quality, the eyespot might have potential additional roles mainly for chloroplast function. There is, e.g., increasing evidence from both, ultrastructural and proteomic data, for a link between eyespot globules (EG) and plastoglobules (PG) and thereby for a more general role in chloroplast metabolism such as the biosynthesis of prenylquinones and carotenoids (Kreimer, 2009; Nacir and Bréhélin, 2013; Davidi et al., 2015). Whereas PG are directly connected with the thylakoids via the outer lipid leaflet of the TM (Austin et al., 2006), it is currently not known whether this is also true for the globules of the eyespot. Methods for the isolation of PG from green algae as well as EG and fragments are established (Kreimer et al., 1991; Renninger et al., 2001; Schmidt et al., 2006; Davidi et al., 2015).

Until 2005, only six proteins relevant to the structure and function of the eyespot of *C. reinhardtii* were identified, mainly based on genetic approaches. These included EYE2 and MIN1, two proteins important for eyespot assembly (Roberts et al., 2001; Dieckmann, 2003), two splicing variants of the abundant retinal binding protein COP, and the two unique seven-transmembrane domain photoreceptors, Channelrhodopsin1 (ChR1), and ChR2 serving as basis for the development of optogenetics (Deininger et al., 1995; Fuhrmann et al., 2001; Nagel et al., 2002; Sineshchekov et al., 2002; Suzuki et al., 2003; Hegemann and Nagel, 2013). As an in depth knowledge of the protein composition of this complex organelle is one prerequisite to understand its function at a molecular level, also proteomic approaches to a fraction strongly enriched in eyespot fragments were applied. Thereby, 203 different proteins covered with at least two different peptides were identified and some of them were shown to be phosphorylated (Schmidt et al., 2006; Wagner et al., 2008). Based on these proteomic approaches, recent functions in eyespot development have been demonstrated for SOUL3, one of the five SOUL heme-binding proteins in *C. reinhardtii* (Merchant et al., 2007; Schulze et al., 2013), Casein kinase 1 and the blue-light receptor Phototropin (Schmidt et al., 2006; Trippens et al., 2012). Additionally, evidence for a specialized localization of the alpha- and beta-subunit of the chloroplast ATP synthase and thereby probably function within the eyespot was found (Schmidt et al., 2007). The eyespots used in the proteomic approach by Schmidt et al. (2006) retained, however, only small parts of the eyespot membranes. Thus, it is well feasible that core membrane-associated eyespot proteins were not identified in this study. To further increase the understanding of eyespot related signaling processes in depth, knowledge of the protein composition of the membranes in its close vicinity is desirable. We thus developed a method that allows isolation of intact eyespots containing the two layers of lipid rich globules still associated with large parts of the eyespot membranes and the adjacent PM as well as chloroplast envelope areas and analyzed this fraction by a proteomic approach.

Beside phosphorylation, protein methylation is an important posttranslational modification involved in the regulation of protein stability, localization, activity, and protein–protein interactions (Reinders and Sickmann, 2007; Biggar and Li, 2015). The importance of protein methylation at Arg and Lys residues in regulating protein activity is also becoming apparent in vascular plants and algae (e.g., Deng et al., 2010; Blifernez et al., 2011; Werner-Peterson and Sloboda, 2013). Recently, Arg and Lys methylation was reported for several chloroplast proteins (Alban et al., 2014). We therefore assigned protein methylation sites in the previous and currently analyzed eyespot fractions. In this study, we identify 742 proteins of the extended eyespot fraction, including numerous membrane-associated proteins as intended beside other candidates for eyespot development and signaling. We also assign protein methylation sites on two proteins with already demonstrated important functions for the eyespot (SOUL3 and EYE2; Roberts et al., 2001; Boyd et al., 2011; Schulze et al., 2013) and on 23 other proteins from the eyespot fractions, among them the alpha-, beta- and I-subunits of the chloroplast ATP synthase and a 14-3-3 protein.

## Materials and Methods

### Isolation of the Different Eyespot Fractions

Growth of 20 L *C. reinhardtii* strain cw15 to late log-phase and isolation of fraction 1 (**Figure 1B**) enriched in eyespot fragments was done as previously described (Schmidt et al., 2006). The isolation of a fraction enriched in largely intact eyespots (F2A_e_ in **Figure 1C**) was achieved by reducing the power and duration of the ultrasonic treatment (Bandelin Sonopuls HD2070, Microtip HS 73) to 16% output and seven cycles (15 s each interrupted by 15 s of cooling) during cell rupture. In addition, the submersion depth of the sonotrode tip was reduced to 1.5 cm. Eyespot fragments were separated by discontinuous sucrose gradients buffered with gradient stock solution (GSS) consisting of 12.5 mL sample brought to 42% (w/v) sucrose, 10 mL 31.8% (w/w), and 10 mL 20.5% (w/w) sucrose, overlaid with 5.5 mL GSS. After centrifugation (100,000 *g*, 105 min, 4°C), the orange-green bands (F2_e_ in **Figure 1C**) at the interface of 20.5 and 31.8% sucrose were collected and brought to 35% (w/v) sucrose. Fraction F2_e_ was further purified by flotation centrifugation (100,000 *g*, 60 min, 4°C) on discontinuous sucrose gradients (15 mL sample, 3 mL 31.8% [w/w] sucrose, 18 mL 25% [w/w] sucrose, and 2 mL 20.5% [w/w] sucrose). The yellow–orange 20.5% sucrose fractions (F2A_e_) were collected and brought to 32% (w/v) sucrose. For concentration, 10 ml of this fraction were layered on 26 mL 42% (w/w) sucrose overlaid by 2 mL GSS and centrifuged again (100,000 *g*, 45 min, 4°C). The concentrated fraction F2A_e_ was collected from the top of the gradient, directly extracted with chloroform:methanol:water (4:8:3) and the precipitated proteins were washed several times with methanol:chloroform (2:1, v/v) to remove lipids and pigments before they were dissolved in 2x SDS sample buffer. Cell harvesting, buffers and all other treatments were otherwise done as described by Schmidt et al. (2006).

### Crude Extract Preparation and Electrophoretic Methods

Log phase cultures were supplemented directly prior to cell harvesting with 1 mM phenylmethylsulfonyl fluoride (PMSF). Cells were harvested by centrifugation (2000 *g*, 10 min, 4°C), and pellets were suspended in TNED buffer (20 mM Tris, 80 mM NaCl, 1 mM EDTA, and 1 mM DTT, pH 7.5) supplemented with 1 mM PMSF and complete protease inhibitor cocktail (Roche), according to the instructions of the supplier. In the case of the cell-wall less strain cw15, aliquots (300 μl) of the suspended cells were directly mixed with methanol:chloroform (2:1, v:v; 1200 μL) for parallel protein precipitation and lipid/pigment removal. Cell wall-possessing strains were homogenized by sonication. Protein solubilization, SDS-PAGE and protein staining with silver or colloidal Coomassie were conducted as described by Schmidt et al. (2006). Gel loading based on equal protein content and immunoblot analyses followed standard techniques. Incubations with the monoclonal anti-modified (monomethyl, asymmetric dimethyl, and symmetric dimethyl) methyl-arginine antibody 7E6 (anti-Rm; company Covalab SAS, France, 1:2,500) were done overnight at 4°C.

### Electron Microscopy

For fixation, aliquots of F2A_e_ were diluted 1:1 with ice-cold 40% (w/v) BSA in GSS and mixed with cold glutaraldehyde (final concentration 3.1 or 6.3 %). The BSA/F2A_e_ gel pieces were sliced in small pieces and fixed overnight in 25 mM Hepes/NaOH (pH 7.8) and 3.5% glutaraldehyde at 4°C. Samples were washed three times for 10 min with 25 mM Hepes/NaOH (pH 7.8) prior to a 2 h incubation at 4°C with osmium tetroxide (1% in 25 mM Hepes/NaOH, pH 7.8), followed by four washing steps with Millipore water. Dehydration, embedding in Epon and staining of ultrathin sections with uranyl acetate/lead citrate was done as described (Reynolds, 1963; Renninger et al., 2001). EM negatives were scanned and processed with Photoshop (Adope Systems).

### Mass Spectrometry (MS) Analysis

#### In-Gel Digestion and Nano HPLC Electrospray Ionization Tandem MS (LC-ESI-MS/MS)

The gel was dissected into 50 bands and each gel slice was subjected to a washing procedure followed by an in gel digestion with trypsin as previously described (Schmidt et al., 2006). The pellets containing the tryptic peptides were resuspended in 5 μL 5% (v/v) acetonitrile/0.1% (v/v) formic acid and subjected to nano LC-ESI-MS using an UltiMate^TM^ 3000 nano HPLC (Dionex Corporation) with a flow rate of 300 nL/min coupled on-line with a linear ion trap electrospray-ionization (ESI) mass spectrometer (Finnigian^TM^ LTQ^TM^, Thermo Electron Corp.) as described previously (Schmidt et al., 2006). The mass spectrometer was cycling between one full MS and MS/MS scans of the four most abundant ions. After each cycle, these peptide masses were excluded from analysis for 3 min.

#### Data Analysis

Data analysis was done using the Proteome Discoverer software (Version 1.4) from Thermo Electron Corp. including the SEQUEST algorithm (Link et al., 1999). Searches were done for tryptic peptides allowing two missed cleavages. The software parameters were set to detect lysine modifications (+14.016 for monomethyl, +28.031 for dimethyl, and +42.047 for trimethyl) and arginine modifications (+14.016 for monomethyl and +28.031 for dimethyl) with a maximum of four modifications of one type per peptide. Further, detection of variable methionine oxidation (+15.995) was enabled. Peptide mass tolerance was set to 1.5 Da in MS mode. In MS^2^ mode, fragment ion tolerance was set up to 1 Da. The parameters for all database searches were set to achieve a false discovery rate (FDR) of not more than 1% for each individual analysis (Veith et al., 2009). All spectra used for the assignment of protein methylation were in addition manually validated and checked for the presence of the b- and/or y-type ions representing the methylation sites. In case that a methylation site is present on the first amino acid at the N-terminus of the peptide and therefore not present in the b- and y-type ions, these methylation sites were considered only when all other possible methylation sites in the peptide were validated as methylated or non-methylated, respectively. In case of trimethylated Lys residues, spectra were additionally screened for the presence of neutral loss events of trimethylamine (59 Da), which is a signature for trimethylation (Zhang et al., 2004; Erce et al., 2012). Data were searched against the *C. reinhardtii* database (Vs. 5.3.1), hosted by Phytozome (Vs. 9.1)^1^, the mitochondrial database available from NCBI (NC001638, [gi:11467088]) and the chloroplast database^2^. Data from all runs were combined and further evaluated using an in house developed program. The peptide sequences of the gene models were compared to the NCBI protein database using BLAST (Altschul et al., 1997). For positive identification of both, protein and functional domain prediction, an internal cut-off *E*-value of 1e-05 was used. Transmembrane domain information was based on predictions by the programs TMHMM (Krogh et al., 2001), TMpred (Hofmann and Stoffel, 1993), and TopPred (von Heinjne, 1992). The GRAVY index was determined with ProtParam (Gasteiger et al., 2005). The mass spectrometry (MS) proteomics data have been deposited to the ProteomeXchange Consortium^3^ via the PRIDE partner repository (Vizcaino et al., 2013) under the project description “Extended eyespot proteome and methylated proteins in the eyespot of *C. reinhardtii* strain cw15 (ID: PXD003254)”.

### Miscellaneous

Protein content was determined according to Neuhoff et al. (1979) with BSA as standard. Chlorophyll and carotenoids were determined as described by Lichtenthaler (1987). Spectra were recorded in 90% methanol with an Ultrospec 2100 pro (GE Healtcare Life Science). Images of gradients, gels and blots were captured with a Coolpix 990 (Nikon) and processed with Photoshop (Adobe Systems).

## Results

### Isolation and Characterization of a Fraction Enriched in Intact Eyespots

Based on a previously established method for the isolation of eyespot fragments from *C. reinhardtii* (Schmidt et al., 2006), we developed a procedure that allows the isolation of largely intact eyespot apparatuses. They contain the two layers of lipid rich globules with large parts of the eyespot associated membranes and adjacent PM areas. As a visual marker for enrichment of eyespots within the sucrose gradients, we first used the conspicuous yellow/orange to red color of the EG (**Figures 1B,C**). We noticed a deep orange-red fraction enriched in highly purified eyespot fragments (named F1; Schmidt et al., 2006), as well as an additional weak yellow–orange fraction (F2) apart from the bulk of the cell and chloroplast debris. F2 accumulates at the interface between 20.5 and 31.8% sucrose. At these sucrose concentrations, enrichment of largely intact eyespots with a high proportion of eyespot-associated membranes was described for the green alga *Spermatozopsis similis* (Kreimer et al., 1991). Thus, it seemed possible that the *Chlamydomonas* F2 fraction that was always present only in minute amounts may also bear intact eyespots. We therefore tried to optimize the yield of fraction F2 by variation of the ultrasonic treatment used for cell rupture. Best results were obtained upon a 50% reduction of the output power and cycle number (32 to 16% and 14 to 7 cycles) in combination with a reduction of the submersion depth of the sonotrode tip to 1.5 cm. For a clear differentiation between the different eyespot preparations, all fractions obtained by this modified cell rupture/purification strategy are characterized by the subscripted “e”, which stands for extended eyespot. Although these reductions result in a clear loss in general cell rupture and thereby total yield of F1_e_, the relative amount of F2_e_, as judged by its color, was increased compared to the standard procedure (**Figures 1B,C**). F2_e_ was further purified by an additional gradient centrifugation step to reduce contamination by thylakoids and other cell organelles. In this subsequent gradient, F2_e_ splits up into two fractions: an orange–yellow (F2A_e_) and a greenish-yellow (F2B_e_) fraction (**Figure 1C**, final gradient). F2A_e_ was finally concentrated by a floating centrifugation step. The total protein yields of F2A_e_ varied between 28 and 46 μg when starting with 20 L of culture.

**FIGURE 2:**
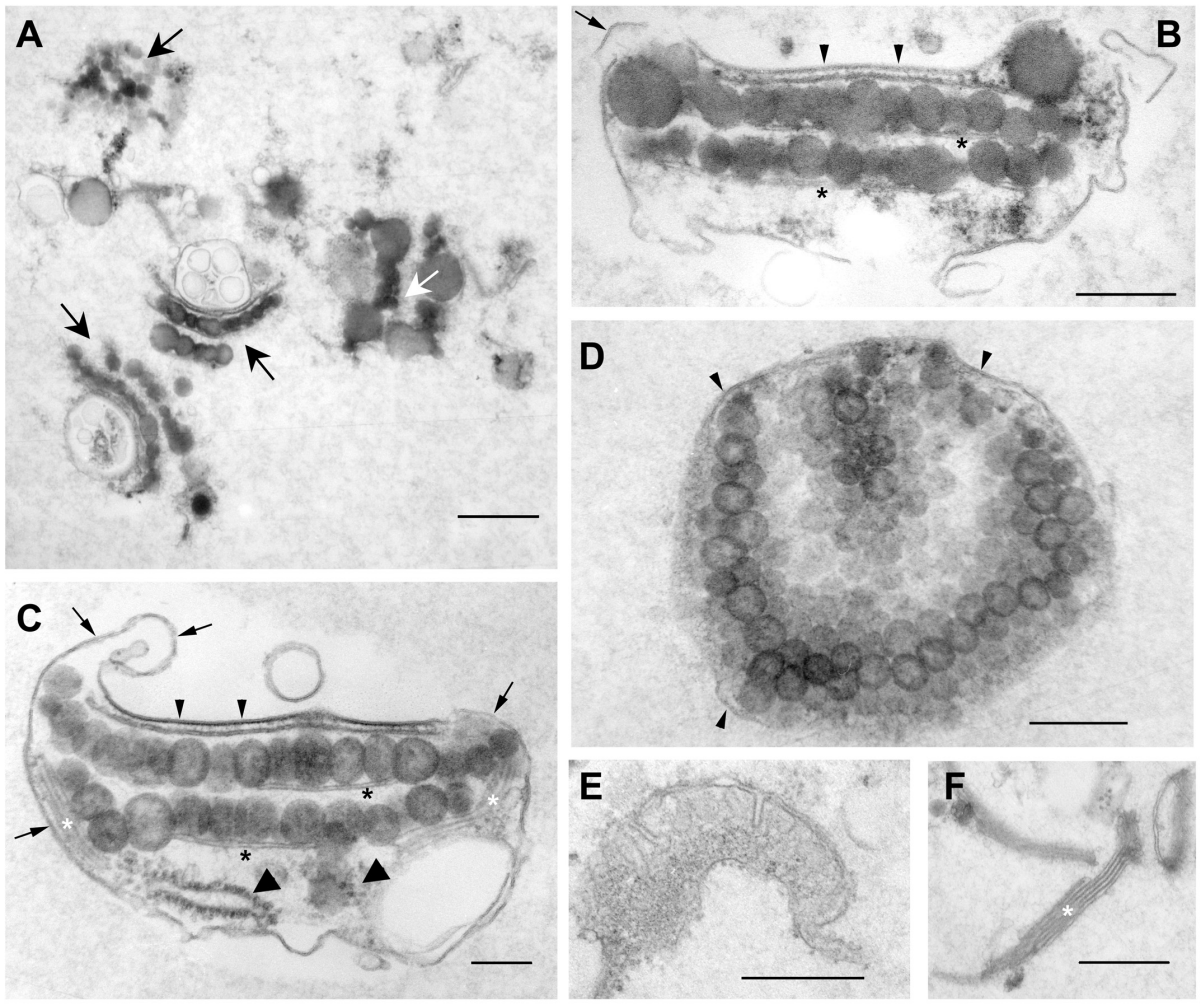
**Characterization of the extended eyespot fraction 2A (F2A_e_) by transmission electron microscopy revealed enrichment of intact eyespots.** Overview **(A)** and details **(B–F)**. **(A)** Black arrows indicate well-preserved eyespots, whereas the white arrow indicates structurally less well-preserved eyespots with fused EG; scale bar: 600 nm. **(B,C)** Cross-sections through isolated, double-layered eyespots associated with eyespot and other membranes. The globule layers are subtended by a thylakoid (black asterisk). Small arrowheads: plasma membrane area overlying the EG. Note the close association with the chloroplast envelope membranes and the thickened, electron-dense appearance of the plasma membrane in this region. In addition, the fuzzy fibrillar material typically observed *in situ* between the plasma membrane and chloroplast envelope in the region of the eyespot apparatus (Kreimer, 2009) is preserved. Small arrows point to normal plasma membrane regions tending to enwrap the globule layers and not eyespot-associated structures like a thylakoid stack (white asterisk) or ribosomes (large arrowheads); scale bars: 150 nm. **(D)** Tangential section through a globule layer. Arrowheads indicate membranes enclosing the eyespot; scale bar: 250 nm. **(E,F)** Co-isolated free mitochondrial fragment **(E)** and free thylakoid stack **(F)**; scale bars: 400 nm.

To verify enrichment of eyespots and to judge their intactness and purity, F2A_e_ was analyzed by transmission electron microscopy (**Figure 2**). Indeed, a significant number of well-defined eyespots with intact globule layers are enriched in this fraction (**Figures 2A–D**). The close packing of the globules is largely preserved and the layers are still subtended by a thylakoid. The average size of the globules in these layers is well in the *in situ* range of 80–130 nm (Melkonian and Robenek, 1984). Analyses of 100 globules from different eyespots and sections yielded values of 114 ± 28 nm for the width and 121 ± 30 nm for the height, respectively. Only a few EG were enlarged, probably by fusion. In addition, large parts of the chloroplast envelope, the PM and a fuzzy appearing material observed between the chloroplast envelope and the PM in the eyespot region *in situ* were frequently present (**Figures 2B,C**; Melkonian and Robenek, 1984; Kreimer, 2009). Additionally, less well-preserved eyespots predominantly with enlarged, possibly fused, globules were also observed (**Figure 2A**). Along with the intact eyespots, still possessing all eyespot membranes, varying amounts of the adjacent PM and chloroplast envelope were co-isolated (**Figures 2B,C**). Depending on their length, these membranes partially enwrapped the isolated eyespots as well as cell components not belonging to the functional eyespot, but located *in situ* in its close proximity like, e.g., grana thylakoids and ribosomes (**Figure 2C**). Major free contaminants were individualized fused globules associated with membranes, membrane vesicles of unknown origin as well as stroma and grana thylakoids (**Figures 2A,F**). Occasionally, also mitochondrial fragments were detected (**Figure 2E**). In summary, transmission electron microscopy confirmed enrichment of intact eyespots associated with all eyespot membranes. The gain in intactness of eyespots seems, however, accompanied by an inevitable increase of non-eyespot contaminants.

**FIGURE 3:**
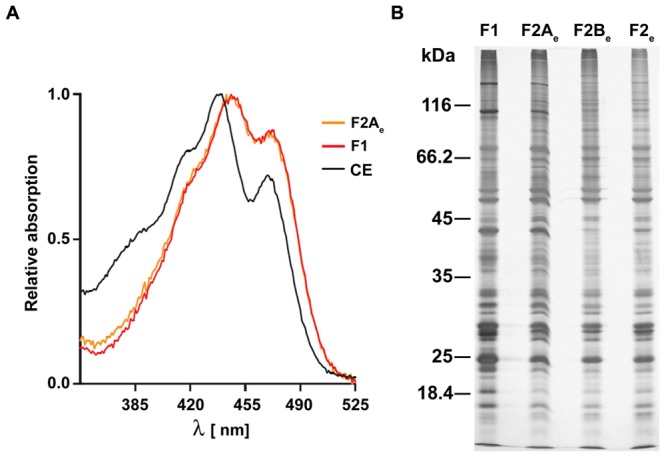
**Spectral analysis and SDS-PAGE of the different eyespot fractions. (A)** Normalized absorption spectra of fractions F1 and F2A_e_ in 90% methanol in comparison to that of a crude cell extract (CE). **(B)** Comparison of the protein patterns of fractions F1, F2A_e_, F2B_e_, and F2_e_. Total proteins (10 μg) of the different gradient fractions were separated by SDS-PAGE (11%) and stained with silver. The positions of molecular mass markers are indicated on the left in kilodaltons.

Spectral analysis of the pigments in fraction F2A_e_ was performed for further characterization. The visual appearance of F2A_e_ already indicates that carotenoids are strongly enriched. In vegetative cells of *C. reinhardtii*, carotenoids are found in the thylakoids, the chloroplast envelope and EG, whereas chlorophyll is solely present within thylakoids. A comparison of the amount of chlorophyll present in the crude extract (CE) with the amount of chlorophyll in fraction F2A_e_ revealed that 0.015% of the total chlorophyll applied to the gradients remained there. The average carotenoid:chlorophyll ratio measured as the absorbance ratio 478 to 680 nm was 40 ± 12.6. These values indicate effective removal of the majority of free thylakoids not associated with the EG layers. Compared to fraction 1, however, these values are significantly higher (<0.0005% and 60–70; Schmidt et al., 2006). Spectra of methanol extracts of fraction F2A_e_ were identical to those of fraction F1 in the range between 350 and 525 nm and revealed a typical carotenoid spectrum (**Figure 3A**). Major absorption peaks where at 446 and 470 nm and a shoulder at 423 nm.

The above analyses clearly shows that eyespot purity of the extended eyespot fraction F2A_e_ was reduced compared to that of fraction F1 (Schmidt et al., 2006). Nonetheless, the significantly increased amount of eyespot associated membranes as well as of membrane areas adjacent to them in fraction F2A_e_ offers the possibility to identify novel putative eyespot proteins and those present in close vicinity to this visual system via a proteomic approach. To get a first impression of possible differences in the protein patterns of the eyespot fractions, a comparative SDS-PAGE analysis was conducted (**Figure 3B**). As expected, the general protein pattern of the fractions enriched in eyespot fragments (F1) and intact eyespots (F2A_e_) were similar to some extent indicating an overlap of a majority of proteins. In the complex protein patterns, however, also subtle but clear differences were resolved (see, e.g., the molecular mass ranges between 35 and 45 kDa and below 25 kDa). Their protein patterns clearly differed also from those fractions with higher chlorophyll content (F2_e_ and F2B_e_). The protein pattern of fraction F2A_e_ was highly reproducible in independent purifications [Supplemental Figure S1A (Data Sheet 1)].

### The Proteome of the Extended Eyespot

To identify proteins of the enriched F2A_e_ eyespot fraction by MS/MS, proteins from two independent eyespot isolations were combined, separated by SDS-PAGE and the gel was sliced into 50 pieces [Supplemental Figure S1B (Data Sheet 1)]. Following in-gel digestion with trypsin, the generated peptide fragments were subjected to LC-ESI-MS/MS analyses using a linear ion trap mass spectrometer (see Materials and Methods). In total, 742 proteins were identified with at least two different peptides and a FDR of ≤1. The majority of the 203 proteins identified in the former fraction F1 with at least two different peptides (Table 1 in Schmidt et al., 2006) were positively confirmed to be present in the extended eyespot of F2A_e_ [Supplemental Table S1 (Data Sheet 3) along with the currently identified peptides and values]. Supplemental Table S2 (Data Sheet 4) lists the 27 missing candidates, 10 of them (category ribosomes, translation, and DNA related) being obvious contaminants. Reasons for the differences are discussed later.

A selection of promising novel candidates with regard to eyespot development, signal transduction as well as membrane association and transporters is presented in **Table 1**. This selection is based on current knowledge about the eyespot apparatus, including its different complex associated subcellular structures as well as signaling pathways important for photoorientation, its development including mutant analysis as well as information on PG. Detailed information for all identified peptides of these selected candidates is given in Supplemental Table S3 (Data Sheet 5). We think that these categories enclose the primarily most interesting candidates to be studied functionally in the future. Supplemental Table S4 (Data Sheet 6) lists all further identified novel proteins along with their peptides from F2A_e_ including also the putative contaminants. Among the novel candidates (**Table 1**), a protein involved in eyespot assembly, the Ser/Thr kinase EYE3 (Boyd et al., 2011), a retinal pigment epithelial membrane receptor as well as a protein with homologies to the eye pigment precursor transporter protein family (Vopalensky et al., 2012) were found. Moreover, two SOUL heme-binding proteins (SOUL2 and SOUL5) were identified. The so far known SOUL3 eyespot protein influences size and position of the eyespot (Schulze et al., 2013). Excitation of the ChR photoreceptors initiates a Ca^2+^-based signaling cascade toward the flagella in the eyespot (reviewed by Hegemann and Berthold, 2009). Further, rapid reversible protein phosphorylation in isolated eyespots is strongly affected by free Ca^2+^ concentrations between 10^-8^ and 10^-7^ M (Linden and Kreimer, 1995) and both ChRs as well as SOUL3 are targets of kinases (Wagner et al., 2008). Therefore, the identification of six novel calcium-sensing and binding proteins as well as 11 different kinases and four phosphatases could be of high interest for understanding the signaling cascade network in the eyespot. Notably, a Ca^2+^-dependent protein kinase 1 was identified with 11 different peptides. Moreover, two 14-3-3 proteins that interact with phosphorylated proteins and are key regulators in many vital cellular processes including signal transduction (Denison et al., 2011) were identified with eight and ten different peptides, respectively. Of note is also the fact that some of the kinases belong to the ABC1 kinase family such as EYE3 (**Table 1**). Homologs of two members of this family identified in the proteome of eyespot fraction F1 are present in PG of *Arabidopsis thaliana* and the beta-carotene PG of *Dunaliella bardawil* (Ytterberg et al., 2006; Lundquist et al., 2012; Davidi et al., 2015). Two of the newly identified ABC1 kinases with an AarF domain from the F2A_e_ fraction [Cre09.g407800.t1.3 (AKC1), Cre13.g570350.t1.3 (AKC4)] are also present in *Arabidopsis* PG (Lundquist et al., 2012). Additionally, AKC1 has a homolog in the plastoglobule proteome of *D. bardawil* (Davidi et al., 2015).

**Table 1:** Functional categorization of newly identified proteins from the extended eyespot fraction F2A_e_.

Transcript name (Phytozome)	No. of different peptides	Function and/or homologies	TMDs^a^
**Proteins important for eyespot development/retina related proteins**			
Cre02.g105600.t1.3	4	Eyespot assembly protein EYE3, ABC1 kinase family	(+)
Cre12.g547300.t1.3	3	Eye Pigment Precursor Transporter (EPP) family protein	+
Cre12.g488350.t1.3	2	Retinal pigment epithelial membrane receptor	(+)
**SOUL heme-binding proteins**			
Cre13.g566850.t1.2	2	Similar to SOUL2	(+)
Cre06.g292400.t1.3	2	Similar to SOUL5	(+)
**14-3-3 proteins**			
Cre12.g559250.t1.2	10	14-3-3 protein	-
Cre06.g257500.t1.2	8	14-3-3 protein	-
**Kinases^b^**			
Cre17.g705000.t1.2	11	Calcium-dependent protein kinase 1	(+)
Cre09.g407800.t1.3	10	ABC1/COQ8 ser/thr kinase with an AarF (predicted unusual kinase) domain	(+)
Cre16.g663200.t1.3	10	Cyclic nucleotide dependent protein kinase	(+)
g8097.t1	9	Predicted protein with AarF (predicted unusual protein kinase) domain	(+)
g8097.t2^c^	5	Predicted protein with AarF (predicted unusual protein kinase) domain	(+)
Cre13.g570350.t1.3; Cre13.g570350.t2.1	6	ABC-1-like kinase with an AarF (predicted unusual protein kinase) domain	+
g13907.t1	4	Predicted protein with AarF (predicted unusual protein kinase) domain	(+)
Cre06.g307100.t1.3	3	ABC1/COQ8 ser/thr kinase with an AarF domain	(+)
Cre16.g657350.t1.2	3	Protein with catalytic domain of serine/threonine protein kinases	(+)
Cre03.g168150.t1.2	2	Protein with catalytic domain of tyrosine kinase	(+)
Cre13.g571700.t1.3	2	Protein with catalytic domain of serine/threonine protein kinases	(+)
g17359.t1	2	Protein with catalytic domain of serine/threonine protein kinases	(+)
**Phosphatases^b^**			
Cre06.g292550.t1.2	3	Protein phosphatase 1	-
Cre09.g388750.t1.2	3	Phosphoinositide phosphatase	+
Cre06.g257850.t1.2	2	Serine/threonine protein phosphatase	-
Cre01.g030200.t1.2	2	Protein phosphatase 2C-like protein	-
**Calcium-sensing and binding proteins**			
Cre11.g468450.t1.2	6	Similar to centrin	-
Cre14.g615750.t1.1	4	Protein with EF-hand, calcium binding motif	(+)
Cre12.g559450.t1.3	4	Protein with a C2 domain (found in kinases and membrane trafficking proteins)	+
Cre03.g150300.t1.2	3	Protein with EF-hand, calcium binding motif	-
Cre03.g178150.t1.1	2	Similar to calmodulin	-
Cre15.g641250.t1.2	2	Protein with EF-hand, calcium binding motif	-
**Membrane-associated/structural proteins, proteins with PAP-fibrillin domain**			
Cre13.g583550.t1.2	11	VIPP1, Vesicle inducing protein in plastids 1	-
Cre03.g197650.t1.2	8	Protein with PAP-fibrillin domain	(+)
Cre12.g502250.t1.2	4	Protein with PAP-fibrillin domain	+
Cre12.g492600.t1.2	3	Fasciclin-like protein	+
Cre12.g492650.t1.2	2	Fasciclin-like protein	+
Cre11.g478850.t1.2	2	Protein with PAP-fibrillin domain	(+)
**Transporter**			
Cre10.g459200.t1.2	11	Plasma membrane-type proton ATPase	+
Cre04.g220200.t2.1; Cre04.g220200.t1.2; Cre04.g220200.t3.1	11	K^+^/H^+^-eﬄux antiporter 2 (KEA2, chloroplast inner envelope)	+
Cre09.g388850.t1.1	4	Calmodulin binding calcium-transporting ATPase (P-type/plasma membrane)	+
Cre03.g164600.t1.2;	4	Plasma membrane hydrogen ATPase	+
Cre03.g165050.t1.2	2	Plasma-membrane proton-eﬄux P-type ATPase	+

*^a^transmembrane domains, predictions done with TMHMM, TMpred, and TopPred (+, TMDs predicted by all three programs; (+), TMDs predicted by two programs; –, TMDs predicted by only one or no program); ^b^Kinases and phosphatases that are putative signaling related; ^c^Splice variant of g8097.t1. Function and/or homologies of depicted proteins were determined by NCBI BLASTp.*

Among the novel candidates in the group of membrane associated/structural proteins are three PAP-fibrillin domain-containing proteins. Eight proteins of this group important for stabilization of PG and EG (Renninger et al., 2001; Ytterberg et al., 2006) were already identified in our previous analysis (Schmidt et al., 2006). In addition, Vesicle inducing protein in plastids (VIPP1) that is involved in the biogenesis of thylakoids (Rütgers and Schroda, 2013) has been also found with 11 different peptides (**Table 1**). VIPP1 was detected recently in an algal plastoglobule proteome and it was postulated that PG evolved from eyespot lipid droplets (Davidi et al., 2015). In addition, two fasciclin-like proteins with three and four FAS1 domains, respectively, might be of interest. FAS1 domains are present in many secreted, membrane-anchored proteins. Five transporters were found as well, underlining the enrichment of eyespot associated membranes (**Table 1**). In this group, identification of three P-type PM ATPases and a PM type Calmodulin-binding Ca^2+^-transporting ATPase is of special interest. These types of proteins may be important for the resting membrane potential at the PM and thereby eventually also affect the excitation of the cell through the ChRs. As the currents of these photoreceptors are carried by Ca^2+^, its extrusion in the eyespot region following ChR excitation will be important for signaling and adaptation. Due to the extreme close vicinity of the PM and the chloroplast envelope in the eyespot, also the chloroplast K^+^/H^+^-eﬄux antiporter 2 (KEA2) might affect the resting membrane potential in the eyespot region. KEA2, as one of the PM-type H^+^-ATPases, was found with 11 different peptides. These different types of transporters thus might probably have important indirect functions in the context of eyespot related signaling.

### Assignment of Protein Methylation

**FIGURE 4:**
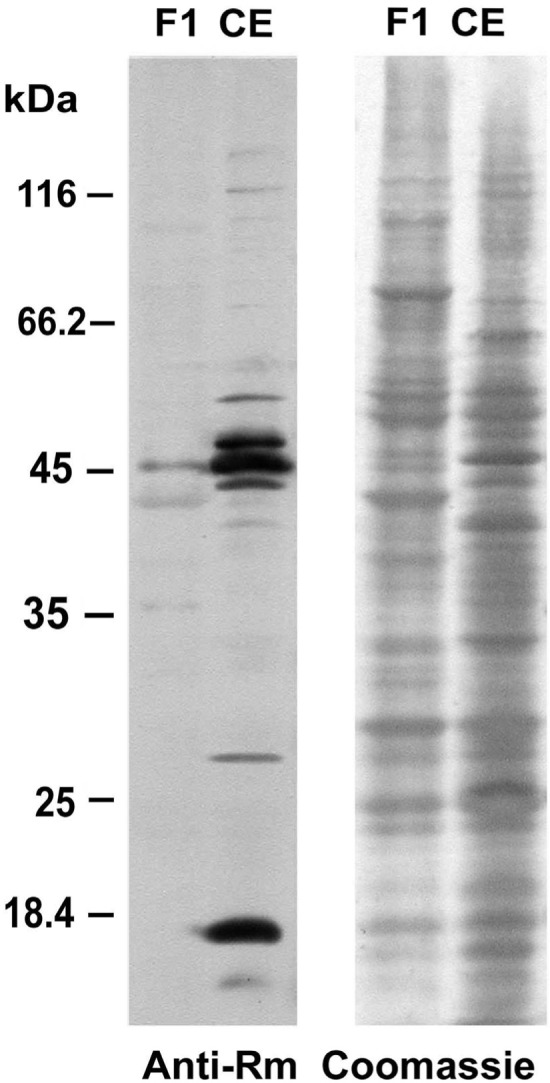
**Western blot analysis of proteins from the eyespot fraction F1 and a CE with the anti-methyl-arginine specific antibody 7E6.** Proteins (8 μg) were separated by 11% SDS-PAGE, transferred to a PVDF membrane, and either analyzed with a monoclonal anti-methyl-arginine antibody (Anti-Rm; 1:2,500) or stained with Coomassie Brilliant Blue R250 (Coomassie).

In recent years, increasing evidence points to the importance of diverse posttranslational protein modifications beside reversible phosphorylation in the regulation of protein stability, localization, activity and protein–protein interactions in cell organelles (e.g., Lehtimäki et al., 2015). One of them is methylation. In a recent study, 23 chloroplast proteins have been shown to be methylated at Lys and/or Arg residues (Alban et al., 2014). Methylation of these amino acids increases their basicity and hydrophobicity without altering their charge (Rice and Allis, 2001). As a part of the functional eyespot involves local specializations of the chloroplast (**Figure 1A**), it was of interest to find out whether methylated proteins are present in the eyespot fractions. Prior to an analysis via MS, we checked with a commercially available anti-methyl-Arg specific antibody recognizing monomethyl, asymmetric dimethyl, and symmetric dimethyl-Arg whether methylated proteins are still detectable after the isolation of eyespots (**Figure 4**). Immunoblot analysis of the proteins from fraction F1 with this antibody revealed four clearly and several weakly labeled protein bands. This result demonstrates that methylated proteins are still present in the eyespot fraction after the isolation procedure. Position, number, and intensity of labeled bands in a CE clearly differed, indicating that a specific subset of methylated proteins may occur in the eyespot fractions.

Mass spectrometry analysis of the peptides from both eyespot fractions resulted in the identification of 25 methylated proteins (**Tables 2** and **3**) where the methylation sites could be specified after additional manual evaluation of the spectra [Supplemental Figure S2 (Data Sheet 2), Supplemental Table S5 (Data Sheet 7)]. These proteins were characterized by 36 different methylated peptides and nine additional overlapping peptides having variations in the methylation status and/or sites and/or in oxidized versus non-oxidized Met. Six proteins are methylated at Lys and Arg residues, 17 proteins only at Lys residues, and two only at Arg residues. In total, we detected 10 Arg sites (nine mono and one dimethyl) and 42 Lys methylation sites (23 mono-, 14 di-, and 8 trimethylations; two of the residues were found mono- as well as dimethylated and one further residue di- as well as trimethylated; **Tables 2** and **3**). It should be mentioned that the identification of trimethylated Lys sides by LC-ESI-MS/MS is hampered by the fact that modification by acetylation is very close in mass (42.04695 vs. 42.01056 Da; Alban et al., 2014). Identification of neutral loss of trimethylamine (59 Da) being specific for trimethylation allows to discriminate (Zhang et al., 2004; Erce et al., 2012; Alban et al., 2014). All seven peptides (**Tables 2** and **3**) with potential trimethylated sites were therefore screened for neutral loss events by analyzing the spectra manually. In all corresponding spectra, b- and y-type ions of neutral loss fragments were found [Supplemental Figure S2 (Data Sheet 2)], indicating that there is trimethylation and not acetylation. Moreover, 14 methylated proteins were identified that are nucleic acid-related or considered as obvious contaminants. They are listed in Supplemental Table S6 (Data Sheet 8).

**Table 2:** Functional categorization of identified methylated proteins from the eyespot fractions F2A_e_ and F1 (Schmidt et al., 2006).

Transcript name (Phytozome)	Function and/or homologies	Methylated peptide	*z*	Xcorr	*x*-times found
**Proteins important for eyespot development**					
Cre16.g666550.t1.2	SOUL3	QRQAFIMNDTCRmFLATDLKm^2a^	3	3.80	2
		–>QRQAFIMoNDTCRmFLATDLKm^2b^	3	3.51	1
Cre12.g509250.t1.1	EYE2, no eyespot	LTDDELIALVNSDPDLDKm^b^	2	4.40	1
**14-3-3 proteins**					
Cre12.g559250.t1.2	14-3-3 protein	DNLTLWTSDMoQDPAAGDDRmEGADMo Km^2^VEDAEP^a^	3	4.37	2
**Photosynthesis/electron transport/light harvesting**					
Cp genome	AtpB, ATP synthase subunit beta	FLSQPFFVAEVFTGSPGKm^2^YVSLAETIEG FGK^a,b^	3	5.97	6
		–>FLSQPFFVAEVFTGSPGKmYVSLAETIE GFGKm^a^	3	3.81	1
		ELQDIIAILGLDELSEEDRm^b^	2	3.51	2
		GMEVVDTGKm^2^PLSVPVGK^a^	2	2.91	1
		–>GMoEVVDTGKPLSVPVGKm^b^	2	3.09	1
		–>GMoEVVDTGKmPLSVPVGK^b^	2	2.81	1
		TVLIMoELINNIAKm^b^	2	2.73	1
Cp genome	AtpA, ATP synthase subunit alpha	SYLANSYPKm^2^YGEILR^a^	2	2.93	1
		SVYEPLATGLVAVDAMoIPVGRm^b^	2	3.38	1
Cp genome	Atpl, CF_0_ ATP synthase subunit I	YVEPAAFLLPINVLEDFTKm^3^PLSLSFR^a^	3	3.11	1
Cre06.g261000.t1.2	10 kDa PS II polypeptide	GKm^2^GYGVYR^b^	1	2.26	2
		YEDKmYGANVDGYSPIYTPDLWTESGDSYTLGTK^a^	3	7.16	1
Cp genome	PetA, Cytochrome f	KmYSEMVVPILSPDPAKm^b^	2	2.60	1
		–>YSEMoVVPILSPDPAKm^b^	2	2.60	1
Cre16.g687900.t1.2	Lhca7, light-harvesting protein of PS I	NPGSQADGSFLGFTEEFKm^a^	2	3.48	5
Cp genome	PsbD, PS II D2 protein	AAEDPEFETFYTKm^a^	2	3.40	1
Cp genome	PsaB, PS I P700 chlorophyll a apoprotein A2	GYWQELIETLVWAHEKmTPLANLVYWKm^a^	3	3.40	1
Cp genome	Ycf4, PS I assembly protein	EIEKmQASELANFLQVSLEA^b^	2	2.77	1
**Transporter**					
Cp genome	CemA (Ycf10), inner envelope protein	FLKm^3^QLFSDVDNLVIQEYR^a^	3	3.10	1
		GSLDSIKmNKm^3^DISK^a^	3	3.00	1
g11711.t1	Similar to ATPase components of ABC transporters	LQTTKIGMLSEGQKm^2^SR^a^	2	3.28	1
**Ferredoxin and thioredoxin-related proteins**					
Cre11.g476750.t1.2	Ferredoxin-NADP reductase	KmGLCSNFLCDATPGTEISMoTGPTGK^a^	2	3.61	1
		–>KmGLCSNFLCDATPGTEISMTGPTGK^a^	2	3.42	1
		IPFWEGQSYGVIPPGTKmINSKm^2a^	3	3.82	1
		–>IPFWEGQSYGVIPPGTKINSKm^3a^	3	3.84	1
**(Lipid) Metabolism**					
g11946.t1, g11946.t2	Similar to Cytochrome b5 reductase	APDYSQGEVSGLLKm^2a^	2	3.32	3
Cre07.g349700.t1.2	Similar to 3-beta hydroxysteroid dehydrogenase/isomerase	ALVRDVSKmATSGSGLLAGVGSTTEVVR^b^	3	3.93	2
		–>ALVRmDVSKATSGSGLLAGVGSTTEVVR^b^	3	3.70	1
Cre01.g017100.t1.3	Similar to proteins with a acylglycerol/acyl-transferase domain	WFESFGAVKASPMAAFRm^2^LLR^a^	3	3.87	1

*Km, methylated Lys; Km^*2*^, dimethylated Lys; Km^*3*^, trimethylated Lys (b and y ions of trimethylamin neutral loss events were found in the corresponding spectra indicating that trimethylation rather than acetylation is present); Rm, methylated Arg; Rm^*2*^, dimethylated Arg; Mo, oxidized Met. If a peptide is marked with an arrow, the peptide above shows overlapping or identical sequences, but the number and/or status of methylation sites may be different or an oxidized Met may be present. ^a^Peptide was identified in an analysis for methylated proteins from the extended eyespot fraction F2A_*e*_. ^b^Peptide was identified in an analysis for methylated proteins from the published eyespot proteome (Schmidt et al., 2006). Function and/or homologies of depicted proteins were determined by NCBI BLASTp.*

**Table 3:** Methylated proteins of unknown function from the eyespot fractions F2A_e_ and F1 (Schmidt et al., 2006).

Transcript name (Phytozome)	Function and/or homologies	Methylated peptide	*z*	Xcorr	*x*-times found
Cre06.g263250.t1.3	No significant hit in NCBI BLASTp	AAVADATGAASSAAADAKm^2a^	2	5.71	7
		AAVADATGAASSAATDAKm^2a^	2	4.20	3
Cp genome	ORF1995 unknown protein	MALEDLSKm^3^WKm^3a,b^	2	2.71	5
		SFDITSMTTTLPFYAGWDESLKm^2a^	2	4.61	3
		–>SFDITSMoTTTLPFYAGWDESLKm^2a^	2	3.59	1
Cre01.g000900.t1.2	Similar to conserved plant/cyanobacterial proteins of unknown functions, contains two DUF1350 domains	LATVAGQLGVSAATAPLEELSRm^a,b^	2	4.03	4
		FKDDSLDDTNNLVQLLQGSSSVGEVLDLTVRm^b^	3	4.35	1
Cp genome	ORF2971 unknown protein	VAMoLAELSLSNLSAKm^3^LDMITDLLVIIDSVRm^a^	3	3.31	1
		MoGQRmKmSQITLLEKm^a^	2	2.52	1
g2947.t1	No significant hit in NCBI BLASTp	ADGAAATATTAATGVLGAGFAKmADEAAASATTAATGVLGAGFAKm^2a^	3	3.67	1
g14174.t1	No significant hit in NCBI BLASTp	GLGDVVGMKm^3^GPAAEINNGR^a^	2	3.33	1
Cre10.g438450.t1.3	No significant hit in NCBI BLASTp	GWGKm^2^LPDSGAALPAFLYKmHVLKm^a^	2	3.13	1

*Km, methylated Lys; Km^*2*^, dimethylated Lys; Km^*3*^, trimethylated Lys (b and y ions of trimethylamin neutral loss events were found in the corresponding spectra indicating that trimethylation rather than acetylation is present); Rm, methylated Arg; Mo, oxidized Met. The peptide that is marked with an arrow shows an identical sequence, but an oxidized Met is present. ^a^Peptide was identified in an analysis for methylated proteins from the extended eyespot fraction F2A_*e*_.^b^Peptide was identified in an analysis for methylated proteins from the published eyespot proteome (Schmidt et al., 2006). Function and/or homologies of depicted proteins were determined by NCBI BLASTp.*

Interestingly, two proteins connected to eyespot development and positioning, EYE2 and SOUL3, as well as one of the 14-3-3 proteins were detected among the methylated proteins. In the 14-3-3 protein, a methylated Arg and a dimethylated Lys residue were detected. As in the chloroplast of *Arabidopsis* (Alban et al., 2014), the *Chlamydomonas* chloroplast ATP synthase subunits alpha and beta both contain methylation sites. In addition, subunit I of the CF_0_ membrane part of ATP synthase bears a trimethylation site. Also, other TM associated components bear methylation sites such as photosystem I and II components or the light harvesting complex protein Lhca7. N-terminal methylation of core light-harvesting complex proteins was shown before in purple photosynthetic bacteria (Wang et al., 2002). Furthermore, the photosystem I assembly protein Ycf4 was methylated at one Lys residue. In addition, methylated peptides of Ycf10, a transporter of the inner chloroplast envelope possibly involved in H^+^ extrusion into the cytosol in algae (Spalding, 2008), and the Ferredoxin-NADP reductase were found. This enzyme of *C. reinhardtii* was already characterized earlier as a methylated protein (Decottignies et al., 1995). It possesses three methylated Lys residues (Lys109, 115, and 161; as the first 26 amino acids were missing in the study from 1995, position of the Lys residues were changed according to the actual sequence of the protein). These positions are remarkably similar to those we obtained (Lys113, Lys162) and coincide in one case (Lys109). Additionally, several proteins of yet unknown functions bear methylation sites (**Table 3**). One protein, Cre01.g000900.t1.2 with two DUF1350 domains, was methylated only at Arg residues.

## Discussion

The functional eyespot apparatus involves parts of numerous subcellular compartments including different membranes, proteins for its development and positioning, as well as proteins known from PG that seem to be involved in its structural organization and preservation. In addition, proteins involved in signaling, adaptational responses, and biochemical pathways, like, e.g., retinal biosynthesis, are part of this light sensitive organelle. Due to its high ultrastructural complexity and its diverse associated processes, selection criteria for proteins associated specifically with the eyespot cannot be derived on the basis of a simple routine workflow procedure. Moreover, conserved targeting sequences for the eyespot are not evident so far and some proteins in the eyespot also occur in additional compartments such as Casein kinase 1 or Phototropin (Schmidt et al., 2006; Trippens et al., 2012). Comparable green algal eyespot proteomes (beside the one in Schmidt et al., 2006) are still missing, but algal lipid droplets and higher plant plastoglobule proteomes are known and can be used for comparison (e.g., Lundquist et al., 2012; Davidi et al., 2015). Moreover, mutant screens for the eyespot are available (e.g., Boyd et al., 2011). All these literature based information has been used for the selection of highly favorable candidates for the extended eyespot proteome listed in **Table 1**. Thereby, we cannot exclude that we have missed certain components or that some false positives were included. For example, light signaling includes reversible phosphorylation, but it is hard to judge if a predicted kinase or phosphatase (without any further information) may be due to a contamination. Thus, additional functional studies as they were done for Casein kinase 1 (Schmidt et al., 2006) and Phototropin (Trippens et al., 2012) that is also present in the cytosol and flagella of *C. reinhardtii*, or the eyespot-specific SOUL3 (Schulze et al., 2013) and the newly identified EYE3 (**Table 1**; Boyd et al., 2011) are crucial. The present listed candidates in **Table 1** provide, however, a good basis for starting such functional tests.

Eyespot components involved in signal transduction are mainly in connection with the light signaling pathway. In the phototactic response of *C. reinhardtii* extracellular Ca^2+^ and Ca^2+^ fluxes are intricately involved. Both ChRs are directly light gated ion channels, which conduct Ca^2+^ under physiological conditions. Their excitation initiates fast inward-directed complex currents in the eyespot region, which finally produce a Ca^2+^ dependent alteration of the flagella beating and thereby leads to the steering of the cell toward or away from the light source (for reviews, see Witman, 1993; Hegemann and Berthold, 2009; Kreimer, 2009). Both ChRs as well as SOUL3, a protein important for the size and position of the eyespot, are targets of kinases (Wagner et al., 2008). Furthermore, in isolated green algal eyespots an increase in the free Ca^2+^ concentration from 10^-8^ to 10^-7^M is known to affect protein phosphorylation and two protein phosphatases with PP2Cc domains are among the dominant proteins in the eyespot proteome (Linden and Kreimer, 1995; Schmidt et al., 2006). Thereby, reversible phosphorylation as well as Ca^2+^ signaling in the eyespot region are primary events after excitation of the ChRs. In this context, the increase in kinases and phosphatases as well as in calcium sensing components in the extended eyespot proteome is of high interest for future studies. Especially the identification of the Calcium dependent protein kinase 1 (CDPK 1) is noteworthy, as no CDPKs have been detected in the core eyespot proteome (Schmidt et al., 2006). The *Chlamydomonas* genome encodes 14 CDPKs; three of them (1, 3, and 11) are found in the flagellar proteome (Pazour et al., 2005; Liang and Pan, 2013). Although CDPK1 has important functions in the flagella, it is also strongly present in the cell body (Liang et al., 2014; Motiwalla et al., 2014). A similar situation is found for Casein kinase 1. This kinase is specifically enriched in both, flagella and the eyespot, and has been shown to be involved in circadian phototaxis as well as in different flagella functions (Schmidt et al., 2006; Wirschell et al., 2011; Boesger et al., 2012). Similarly, the identification of additional members of the ABC kinase family in the extended eyespot proteome could be of interest for eyespot function and development. One of them, EYE3, is already known to be important for eyespot development. Mutants in EYE3 lack a visible eyespot (Lamb et al., 1999; Boyd et al., 2011). Four members of this group found in the eyespot proteomes (Cre13.g581850.t1.2; Cre03.g158500.t1.1; Cre09.g407800.t1.3; Cre13.g570350.t1.3) have homologs in PG of *A. thaliana*. Two of them, which were newly identified in the extended eyespot proteome, are known as AKC1 and AKC4. In higher plants, six of in total eight members of the ABC1-like kinases found in chloroplasts are associated with PG. They play a role in regulation of phylloquinone biosynthesis, redox recycling under high light and probably have other not yet known regulatory functions (Ytterberg et al., 2006; Lundquist et al., 2012; Martinis et al., 2013; Spicher and Kessler, 2015). With two 14-3-3 proteins, which interact with phosphoproteins, members of another protein group often involved in diverse signaling networks including those related to light perception were identified. For example, in higher plants 14-3-3 proteins interact with the blue light receptor Phototropin 1 (Sullivan et al., 2009). In this context, it is interesting that Phototropin in *C. reinhardtii* has recently been shown to affect eyespot size, the content of ChR1 as well as the sign of phototaxis (Trippens et al., 2012).

The procedure for purifying intact eyespots resulted also in an increase in transporters and membrane-associated proteins. Especially the presence of three P-type PM ATPases and a PM type Calmodulin-binding Ca^2+^-transporting ATPase is of special interest, as, e.g., activation of PM ATPases could affect the speed of recovery of the resting membrane potential following excitation of the ChRs. This might be important for, e.g., desensitization and dark recovery of the cell (Govorunova et al., 1997). Also lowering of the cytosolic free Ca^2+^ concentration in the eyespot region following activation of the ChRs is important for signaling and adaptation. The function of the Ca^2+^-ATPase might be complemented by light-induced Ca^2+^ uptake into the chloroplast, which has been demonstrated for isolated chloroplasts and appears to be important for the regulation of the sign of phototaxis in *C. reinhardtii* (Kreimer et al., 1985; Takahashi and Watanabe, 1993). Additionally, the discovery of two fascilin-like proteins with four (Cre12.g492650.t1.2) and three (Cre12.g492600.t1.2) FAS1 domains might be functionally relevant. These are thought to represent ancient cell adhesion domains. As the MORN-repeat motif, which has a critical role in several proteins with functions in the organization of membranous and cytoskeletal structures, these domains may be important for the close contact of the different membrane types in the eyespot region. A MORN-repeat protein is present in the proteomes of both eyespot fractions (Schmidt et al., 2006). It should, however, be noted that for all proteins discussed so far, a role in eyespot development, ultrastructure, positioning or eyespot related signaling needs to be proven experimentally in the future. While the extended eyespot proteome revealed numerous novel eyespot proteins, some were also “lost” compared to the already published one (Schmidt et al., 2006). Thereby, one should consider that the former bioinformatics analysis of the proteome data was based on a so-called probability score. In the current analysis, we are applying a FDR of ≤1%, which is more restrictive. On the other hand, several of the missing candidates (10 out of 27) are putative contaminants. It may also be that the different cell rupture and eyespot isolation methods yielding either eyespot fragments (Schmidt et al., 2006) or the intact structure (present study), have different contaminants associated.

In the past years, it turned out that cellular signal transduction is not only frequently mediated by phosphorylation at Tyr, Ser and Thr but also by methylation at Arg and Lys (Biggar and Li, 2015). Protein methylation is meanwhile considered as an integral part of cellular biology. Protein Lys methyltransferases (PKMTs, often containing a so-called SET domain) and protein Arg methyltransferases (PRMTs) have been well characterized, while Lys and Arg demethylases are less well studied so far or still under debate in case of Arg demethylases (Alban et al., 2014). In *A. thaliana*, six of the SET-domain containing PKMT’s are known or predicted to be targeted to the chloroplast (Table 1 in Alban et al., 2014). A search for homologous PKMTs in the *C. reinhardtii* genome, which was based on the *Arabidopsis* candidates, revealed five putative protein Lys methyltransferases. They have either a Rubisco-lysine *N*-methyltransferase domain [Cre16.g661350.t1.2 (RMT1), Cre12.g524500.t.1.2 (RMT2), and Cre12.g503800.t1.1 (RMT5)], a SET domain (Cre16.g649700.t1.1) or both (Cre12.g541777.t1.1). In case of the ATXR5 protein in *Arabidopsis*, which is supposed to be a histone-lysine methyltransferase, only a limited number of proteins with a very weak homology was found in the *C. reinhardtii* genome. None of them has a methyltransferase domain or a SET domain. Another distantly related group of PKMT’s predicted for *Arabidopsis* chloroplasts (Table 1 in Alban et al., 2014) belongs to the seven-beta strand methyltransferases. One is a ribosomal protein L11 methyltransferase-like protein (PrmA-like) and is also found in *Chlamydomonas* (Cre09.g396735.t2.1). The other is a *S*-adenosyl-L-methionine-dependent methyltransferase (CaMKMT-like) and has only very limited homology (*E*-value: 5.6E^-9^) to a putative *N^2^,N*^2^-dimethylguanosine tRNA methyltransferase (Cre07.g347800.t1.1) in *Chlamydomonas*. In the case of the PRMT’s, five enzymes are known in *C. reinhardtii* (summarized in Werner-Peterson and Sloboda, 2013). PRMTs 1, 2, 3, and 6 are Type I enzymes, which produce asymmetric dimethyl Arg residues, whereas PRMT5 is a Type II enzyme and produce symmetric dimethyl Arg’s. While PRMT1 is known to be localized in flagella (Werner-Peterson and Sloboda, 2013), nothing is known about the cellular localization of the other proteins. In *Arabidopsis* (Alban et al., 2014), only PRMT7 is predicted to be localized in the chloroplast. In *C. reinhardtii*, the closest two homologs to this protein are an uncharacterized PRMT (Cre02.g14626.t1.1) and a Protein/Histone-arginine *N*-methyltransferase [Cre.g558100.t1.2 (PRMT2)]. It seems likely that the methylation events of eyespot proteins located within the chloroplast part of this structure are catalyzed by chloroplast localized methyltransferases. This may even hold true for the methylated 14-3-3 protein, since members of this protein group are also reported to be present in the chloroplast (Sehnke et al., 2000, 2002).

In this context, methylation of known eyespot proteins and proteins forming complexes with eyespot proteins is of interest. Remarkably all of these proteins are localized in chloroplast parts of the eyespot. EYE2 is localized in the chloroplast envelope and important for the formation of the EG layer (Boyd et al., 2011). The photosystem I assembly protein Ycf4, which was found to be methylated at one Lys residue, is known to be in a complex with the eyespot protein COP2 (Ozawa et al., 2009) as well as certain PSI subunits including PsaB, which was also identified as a methylated protein in our analysis. Also the ATP synthase subunits alpha and beta, which are part of the soluble CF1 subunit, are of interest. Both subunits were found as methylated proteins in the chloroplast of *Arabidopsis* (Alban et al., 2014) and they are also present as methylated proteins in the *Chlamydomonas* eyespot. Former studies (Schmidt et al., 2007) showed that the alpha and beta subunits present in the eyespot fraction of *Chlamydomonas* are resistant against thermolysin treatment, which is not the case for the thylakoid-localized alpha and beta subunits of the ATP synthase and other membrane-associated proteins in the eyespot fraction. Therefore, it was assumed that a significant proportion of these ATP-synthase subunits have a specialized localization and function within the eyespot, possibly between the EG. Blue native PAGE of thermolysin-treated eyespots followed by SDS-PAGE even revealed that the alpha and beta subunits are present in conjunction with the gamma-subunit in a thermolysin resistant complex (Schmidt et al., 2007). Thus, methylation of these subunits is of special interest with regard to the conservation of methylation sites compared to *Arabidopsis* chloroplasts but also with regard to potential special functional features of methylation within the eyespot. In *Arabidopsis*, the alpha subunit reveals a methylation site on Arg141. In *Chlamydomonas* eyespots, we detected two methylation sites in this highly conserved protein at Arg161 and Lys470. Although an Arg is present at position 161 in *Arabidopsis*, it is not methylated there. On the other hand, at position 141 in *C. reinhardtii* also a non-methylated Arg is present. The methylated Lys at position 470 is not conserved in *Arabidopsis*. For the beta subunit, we identified six methylated residues in *C. reinhardtii* (Lys96, Lys104, Lys191, Arg418, Lys447, and Lys460) compared to two sites in the *A. thaliana* chloroplast (Alban et al., 2014). For three positions (Lys96, Lys104, Lys460) the corresponding Lys residues are not conserved in *A. thaliana*. Lys191 and Arg418 are conserved, but not methylated in *Arabidopsis*. One of the methylation sites (Lys447), however, fits exactly to one of the two methylation sites that were identified in the ATP synthase subunit beta in the chloroplast of *A. thaliana* (Lys447, Arg52; Alban et al., 2014). It remains to be studied in the future whether the higher methylation status and rather distinct methylation pattern (with one exception) of these two subunits in the eyespot compared to the thylakoids is possibly related to their specialized location in the eyespot. The increased hydrophobicity without altering the charge of the methylated amino acid residues (Rice and Allis, 2001) might be beneficial for their localization between the highly hydrophobic EG.

Among the methylated proteins found in the eyespot fractions is also the SOUL heme binding protein SOUL3 that is localized at the EG and important for a correct positioning of the eyespot and its size (Schulze et al., 2013). It was shown before that SOUL3 is phosphorylated at two sites (Wagner et al., 2008). A crosstalk between methylation–phosphorylation events has been recently found (reviewed in Biggar and Li, 2015). If the methylation and phosphorylation sites are neighbored, this is called “methylation–phosphorylation switch”. Moreover, a methylation–methylation crosstalk has been described. In case of SOUL3, a “methylation–phosphorylation switch” seems rather unlikely as the relevant sites are not directly neighbored. A methylation-methylation crosstalk, however, may exist. SOUL3 methylation sites are at Arg95 and Lys102 while the phosphorylation sites are at Thr42 and Ser44. Notably, both posttranslational modifications are situated at the N-terminal part of SOUL3 while the SOUL/HBP domain is situated at the C-terminus. Methylation of SOUL3 may be involved in regulation of protein–protein interactions. For example, dimethylation of p53 promotes its association with the co-activator protein p53-binding protein 1. Lys methylation can, however, also block protein–protein interactions as demonstrated for the MAPK kinase kinase 2 and its interaction with the serine/threonine protein phosphatase 2A complex (reviewed in Hamamoto et al., 2015). SOUL3 (approximately 45 kDa) has been found to be present in complexes during the day (approximately 100 kDa) and the night phase (approximately 270 kDa) over a circadian cycle (Schulze et al., 2013). It has to be investigated whether methylation affects complex formation of SOUL3 and whether its methylation status changes over a circadian cycle.

The knowledge about the protein composition of the eyespot proteomes as well as about posttranslational modifications such as methylation and phosphorylation provides now an efficient basis for further functional studies. For example, potential effects of protein methylation on protein–protein interactions, protein stability, or subcellular localization can now be put under focus. Signaling components such as EYE2, a protein of the thioredoxin superfamily, or the 14-3-3 protein might recruit interaction partners or induce/inhibit signaling pathways upon methylation. As mentioned before for the ATPase, it is also well visible that eyespot proteins may rely on methylation to increase their hydrophobicity profile especially when situated in between the EG. These emerging functional possibilities will need further attention in future approaches in order to understand this highly complex primordial visual system of a unicellular organism in depth with regard to its structural properties and signaling pathways.

## Author Contributions

GK and MM designed the research. DB, NE, VW, and WW performed the experiments. NE, SG, and GK did the EM analyses; GK, MM, and VW wrote the article with contributions and edits from all other authors.

## Conflict of Interest Statement

The authors declare that the research was conducted in the absence of any commercial or financial relationships that could be construed as a potential conflict of interest.

## Footnotes

1 http://www.phytozome.net/chlamy.php

2 http://www.chlamy.org/chloro/default.html

3 http://proteomecentral.proteomexchange.org

## References

[B1] Alban C. Tardif M. Mininno M. Brugiere S. Gilgen A. Ma S. (2014). Uncovering the protein lysine and arginine methylation network in *Arabidopsis* chloroplasts. *PLoS ONE* 9:e95512 10.1371/journal.pone.0095512 PMC3991674 24748391

[B2] Altschul S. F. Madden T. L. Schäffer A. A. Zhang J. Zhang Z. Miller W. (1997). Gapped BLAST and PSI-BLAST: a new generation of protein database search programs. *Nucleic Acids Res.* 25 3389–3402. 10.1093/nar/25.17.3389 9254694 PMC146917

[B3] Austin J. R. II Frost E. Vidi P.-A. Kessler F. Staehlin L. A. (2006). Plastoglobules are lipoprotein subcompartments of the chloroplast that are permanently coupled to thylakoid membranes and contain biosynthetic enzymes. *Plant Cell* 18 1693–1703. 10.1105/tpc.105.039859 16731586 PMC1488921

[B4] Biggar K. K. Li S. S.-C. (2015). Non-histone protein methylation as a regulator of cellular signaling and function. *Nat. Rev. Mol. Cell Biol.* 16 5–17. 10.1038/nrm3915 25491103

[B5] Blifernez O. Wobbe L. Niehaus K. Kruse O. (2011). Protein arginine methylation modulates light-harvesting antenna translation in *Chlamydomonas reinhardtii*. *Plant J.* 65 119–130. 10.1111/j.1365-313X.2010.04406.x 21175895

[B6] Boesger J. Wagner V. Weisheit W. Mittag M. (2012). Application of phosphoproteomics to find targets of casein kinase 1 in the flagellum of *Chlamydomonas*. *Int. J. Plant Genomics* 2012 581460 10.1155/2012/581460 PMC3536430 23316220

[B7] Boyd J. S. Mittelmeier T. M. Lamb M. R. Dieckmann C. L. (2011). Thioredoxin-family protein EYE2 and Ser/Thr kinase EYE3 play interdependent roles in eyespot assembly. *Mol. Biol. Cell* 22 1421–1429. 10.1091/mbc.E10-11-0918 21372178 PMC3084665

[B8] Davidi L. Levin Y. Ben-Dor S. Pick U. (2015). Proteome analysis of cytoplasmatic and plastidic β-Carotene lipid droplets in *Dunaliella* bardawil. *Plant Physiol.* 167 60–79. 10.1104/pp.114.248450 25404729 PMC4281002

[B9] Decottignies P. Le Maréchal P. Jacquot J. P. Schmitter J. M. Gadal P. (1995). Primary structure and post-translational modification of ferredoxin-NADP reductase from *Chlamydomonas reinhardtii*. *Arch. Biochem. Biophys.* 316 249–259. 10.1006/abbi.1995.1035 7840625

[B10] Deininger W. Kröger P. Hegemann U. Lottspeich F. Hegemann P. (1995). Chlamyrhodopsin represents a new type of sensory photoreceptor. *EMBO J.* 14 5849–5858. 8846778 10.1002/j.1460-2075.1995.tb00273.x PMC394703

[B11] Deng X. Gu L. Liu C. Lu T. Lu F. Lu Z. (2010). Arginine methylation mediated by the *Arabidopsis* homolog of PRMT5 is essential for proper pre-mRNA splicing. *Proc. Natl. Acad. Sci. U.S.A.* 107 19114–19119. 10.1073/pnas.1009669107 20956294 PMC2973915

[B12] Denison F. C. Paul A.-L. Zupanska A. K. Ferl R. J. (2011). 14-3-3 proteins in plant physiology. *Semin. Cell Dev. Biol.* 22 720–727. 10.1016/j.semcdb.2011.08.006 21907297

[B13] Dieckmann C. L. (2003). Eyespot placement and assembly in the green alga *Chlamydomonas*. *Bioessays* 25 410–416. 10.1002/bies.10259 12655648

[B14] Dodge J. D. (1984). The functional and phylogenetic significance of dinoflagellate eyespots. *Biosystems* 16 259–267. 10.1016/0303-2647(83)90009-6 6687045

[B15] Erce M. A. Pang C. N. Hart-Smith G. Wilkins M. R. (2012). The methylproteome and the intracellular methylation network. *Proteomics* 12 564–586. 10.1002/pmic.201100397 22246820

[B16] Foster K. W. Smyth R. D. (1980). Light antennas in phototactic alga. *Microbiol. Rev.* 44 572–630. 7010112 10.1128/mr.44.4.572-630.1980 PMC373196

[B17] Fuhrmann M. Stahlberg A. Govorunova E. Rank S. Hegemann P. (2001). The abundant retinal protein of the *Chlamydomonas* eye is not the photoreceptor for phototaxis and photophobic responses. *J. Cell Sci.* 114 3857–3863. 11719552 10.1242/jcs.114.21.3857

[B18] Gasteiger E. Hoogland C. Gattiker A. Duvaud S. Wilkins M. R. Appel R. D. (2005). “Protein identification and analysis tools on the ExPASy server,” in *The Proteomics Protocols Handbook* ed. Walker J. M. (Totowa, StateNJ: Humana Press) 571–607.

[B19] Gavelis G. S. Hayakawa S. R. A. III Gojobori T. Suttle C. A. Keeling P. J. (2015). Eye-like ocelloids are built from different endosymbiotically acquired components. *Nature* 523 204 10.1038/nature14593 26131935

[B20] Govorunova E. G. Sineshchekov O. A. Hegemann P. (1997). Desensitization and dark recovery of the photoreceptor current in *Chlamydomonas reinhardtii*. *Plant Physiol.* 115 633–642. 12223832 10.1104/pp.115.2.633 PMC158524

[B21] Hamamoto R. Saloura V. Nakamuta Y. (2015). Critical roles of non-histone protein lysine methylation in human tumorigenesis. *Nat. Rev.* 15 110–124. 10.1038/nrc3884 25614009

[B22] Harz H. Nonnengässer C. Hegemann P. (1992). The photoreceptor current of the green alga *Chlamydomonas*. *Philos. Trans. R. Soc. Lond. B Biol. Sci.* 338 39–52. 10.1098/rstb.1992.0127

[B23] Hayakawa S. Takaku Y. Hwang J. S. Horiguchi T. Suga H. Gehring W. (2015). Function and evolutionary origin of unicellular camera-type eye structure. *PLoS ONE* 10:e0118415 10.1371/journal.pone.0118415 PMC4348419 25734540

[B24] Hegemann P. Berthold P. (2009). “Sensory photoreceptors and light control of flagellar activity,” in *The Chlamydomonas Sourcebook* Vol. 3 ed. Witman G. B. (San Diego, CA: Academic Press) 395–429.

[B25] Hegemann P. Nagel G. (2013). From channelrhodopsins to optogenetics. *EMBO Mol. Med.* 5 173–176. 10.1002/emmm.201202387 23339069 PMC3569634

[B26] Hofmann K. Stoffel W. (1993). TMbase – A database of membrane spanning proteins segments. *Biol. Chem. Hoppe Seyler* 374 166.

[B27] Kawai H. Kreimer G. (2000). “Sensory mechanisms: light perception and taxis in algae,” in *The Flagellates: Unity, Diversity and Evolution* eds Leadbeater B. Green J. (London: Taylor & Francis) 124–146.

[B28] Kreimer G. (1994). Cell biology of phototaxis in flagellated algae. *Int. Rev. Cytol.* 148 229–310. 10.1016/S0074-7696(08)62409-2

[B29] Kreimer G. (2009). The green algal eyespot apparatus: a primordial visual system and more? *Curr. Genet.* 55 19–43. 10.1007/s00294-008-0224-8 19107486

[B30] Kreimer G. Brohsonn U. Melkonian M. (1991). Isolation and partial characterization of the photoreceptive organelle for phototaxis of a flagellate green alga. *Eur. J. Cell Biol.* 55 318–327. 1935995

[B31] Kreimer G. Melkonian M. (1990). Reflection confocal laser scanning microscopy of eyespots in flagellate green alga. *Eur. J. Cell Biol.* 53 101–111. 2076697

[B32] Kreimer G. Melkonian M. Latzko E. (1985). An electrogenic uniport mediates light-dependent Ca2+ influx into intact spinach chloroplasts. *FEBS Lett.* 180 253–258. 10.1016/0014-5793(85)81081-4

[B33] Kreimer G. Overländer C. Sineshchekov O. A. Stolzis H. Nultsch W. Melkonian M. (1992). Functional analysis of the eyespot in *Chlamydomonas reinhardtii* mutant ey 627, mt-. *Planta* 188 513–521. 10.1007/BF00197043 24178383

[B34] Krogh A. Larsson B. von Heijne G. Sonnhammer E. L. L. (2001). Predicting transmembrane protein topology with a hidden Markov model: application to complete genomes. *J. Mol. Biol.* 305 567–580. 10.1006/jmbi.2000.4315 11152613

[B35] Lamb M. R. Dutcher S. K. Worley C. K. Dieckmann C. L. (1999). Eyespot-assembly mutants in *Chlamydomonas reinhardtii*. *Genetics* 153 721–729. 10511552 10.1093/genetics/153.2.721 PMC1460774

[B36] Lehtimäki N. Koskela M. M. Mulo P. (2015). Post-translational modifications of chloroplast proteins: an emerging field. *Plant Physiol.* 168 768–775. 10.1104/pp.15.00117 25911530 PMC4741338

[B37] Liang Y. Pan J. (2013). Regulation of flagellar biogenesis by a calcium dependent protein kinase in *Chlamydomonas reinhardtii*. *PLoS ONE* 8:e69902 10.1371/journal.pone.0069902 PMC3723818 23936117

[B38] Liang Y. Pang Y. Wu Q. Hu Z. Han X. Xu Y. (2014). FLA8/KIF3B phosphorylation regulates kinesin-II interaction with IFT-B to control IFT entry and turnaround. *Dev. Cell* 30 585–597. 10.1016/j.devcel.2014.07.019 25175706

[B39] Lichtenthaler H. K. (1987). Chlorophylls and carotenoids: pigments of photosynthetic biomembranes. *Methods Enzymol.* 148 350–382. 10.1016/0076-6879(87)48036-1

[B40] Linden L. Kreimer G. (1995). Calcium modulates rapid protein phosphorylation/de-phosphorylation in isolated eyespot apparatuses of the green alga *Spermatozopsis similis*. *Planta* 197 343–351. 10.1007/BF00202656

[B41] Link A. J. Eng J. Schieltz D. M. Carmack E. Mize G. J. Morris D. R. (1999). Direct analysis of protein complexes using mass spectrometry. *Nat. Biotechnol.* 17 676–682. 10.1038/10890 10404161

[B42] Lundquist P. K. Poliakov A. Bhuiyan N. H. Zybailov B. Sun Q. van Wijk K. J. (2012). The functional network of the *Arabidopsis plastoglobule* proteome based on quantitative proteomics and genome-wide coexpression analysis. *Plant Physiol.* 158 1172–1192. 10.1104/pp.111.193144 22274653 PMC3291262

[B43] Martinis J. Glauser G. Valimareanu S. Kessler F. (2013). A chloroplast ABC1-like kinase regulates vitamin E metabolism in *Arabidopsis*. *Plant Physiol.* 162 652–662. 10.1104/pp.113.218644 23632854 PMC3668060

[B44] Melkonian M. Robenek H. (1984). The eyespot apparatus of green algae: a critical review. *Prog. Phycol. Res.* 3 193–268.

[B45] Merchant S. S. Prochnik S. E. Vallon O. Harris E. H. Karpowicz S. J. Witman G. B. (2007). The evolution of key animal and plant functions is revealed by analysis of the *Chlamydomonas* genome. *Science* 318 245–251. 10.1126/science.1143609 17932292 PMC2875087

[B46] Motiwalla M. J. Sequeira M. P. D’Souza J. S. (2014). Two calcium-dependent protein kinases from *Chlamydomonas reinhardtii* are transcriptionally regulated by nutrient starvation. *Plant Signal. Behav.* 9 e27969 10.4161/psb.27969 PMC4091517 24514873

[B47] Nacir H. Bréhélin C. (2013). When proteomics reveals unsuspected roles: the plastoglobule example. *Front. Plant Sci.* 4:114 10.3389/fpls.2013.00114 PMC3635846 23630540

[B48] Nagel G. Ollig D. Fuhrmann M. Kateriya S. Musti A. M. Bamberg E. (2002). Channelrhodopsin-1: a light-gated proton channel in green algae. *Science* 296 2395–2398. 10.1126/science.1072068 12089443

[B49] Neuhoff V. Phillip K. Zimmer H. G. Mesecke S. (1979). A simple versatile sensitive and volume-independent method for quantitative protein determination which is independent of other external influences. *Hoppe Seylers Z. Physiol. Chem.* 360 1657–1670. 10.1515/bchm2.1979.360.2.1657 92445

[B50] Ozawa S. Nield J. Terao A. Stauber E. J. Hippler M. Koike H. (2009). Biochemical and structural studies of the large Ycf4-photosystem I assembly complex of the green alga *Chlamydomonas reinhardtii*. *Plant Cell* 8 2424–2442. 10.1105/tpc.108.063313 19700633 PMC2751955

[B51] Pazour G. J. Agrin N. Leszyk J. Witman G. B. (2005). Proteomic analysis of a eukaryotic cilium. *J. Cell Biol.* 170 103–113. 10.1083/jcb.200504008 15998802 PMC2171396

[B52] Reinders J. Sickmann A. (2007). Modificomics: posttranslational modifications beyond protein phosphorylation and glycosylation. *Biomol. Eng.* 24 169–177. 10.1016/j.bioeng.2007.03.002 17419095

[B53] Renninger S. Backendorf E. Kreimer G. (2001). Subfractionation of eyespot apparatuses from the green alga *Spermatozopsis similis*: isolation and characterization of eyespot globules. *Planta* 213 51–63. 10.1007/s004250000473 11523656

[B54] Reynolds E. (1963). The use of lead citrate at high pH as an electron-opaque stain in electron microscopy. *J. Cell Biol.* 17 208–212. 10.1083/jcb.17.1.208 13986422 PMC2106263

[B55] Rice J. C. Allis C. D. (2001). Histone methylation versus histone acetylation: new insights into epigenetic regulation. *Curr. Opin. Cell Biol.* 13 263–273. 10.1016/S0955-0674(00)00208-8 11343896

[B56] Roberts D. G. W. Lamb M. R. Dieckmann C. L. (2001). Characterization of the eye2 gene required for eyespot assembly in *Chlamydomonas reinhardtii*. *Genetics* 158 1037–1049. 11454753 10.1093/genetics/158.3.1037 PMC1461727

[B57] Rütgers M. Schroda M. (2013). A role of VIPP1 as a dynamic structure within thylakoid centers as sites of photosystem biogenesis? *Plant Signal. Behav.* 8 e27037 10.4161/psb.27037 PMC4091218 24300099

[B58] Schmidt M. Geßner G. Luff M. Heiland I. Wagner V. Kaminski M. (2006). Proteomic analysis of the eyespot of *Chlamydomonas reinhardtii* provides novel insights into its components and tactic movements. *Plant Cell* 18 1908–1930. 10.1105/tpc.106.041749 16798888 PMC1533972

[B59] Schmidt M. Luff M. Mollwo A. Kaminski M. Mittag M. Kreimer G. (2007). Evidence for a specialized localization of the chloroplast ATP-synthase subunits α, ß and γ in the eyespot apparatus of *Chlamydomonas reinhardtii* (Chlorophyceae). *J. Phycol.* 43 284–294. 10.1111/j.1529-8817.2007.00331.x

[B60] Schulze T. Schreiber S. Iliev D. Boesger J. Trippens J. Kreimer G. (2013). The SOUL heme-binding protein 3 of *Chlamydomonas reinhardtii* influences size and position of the eyespot. *Mol. Plant* 6 931–944. 10.1093/mp/sss137 23180671

[B61] Sehnke P. C. Henry R. Cline K. Ferl R. J. (2000). Interaction of plant 14-3-3- protein with the signal peptide of a thylakoid-targeted chloroplast precursor protein and the presence of 14-3-3 isoforms in the chloroplast stroma. *Plant Physiol.* 122 235–241. 10.1104/pp.122.1.235 10631267 PMC58862

[B62] Sehnke P. C. Rosenquist M. Alsterfjord M. DeLille J. Sommarin M. Larsson C. (2002). Evolution and isoform specificity of plant 14-3-3 proteins. *Plant Mol. Biol.* 50 1011–1018. 10.1023/A:1021289127519 12516868

[B63] Sineshchekov O. A. Jung K.-H. Spudich J. L. (2002). Two rhodopsins mediate phototaxis to low- and high-intensity light in *Chlamydomonas reinhardtii*. *Proc. Natl. Acad. Sci. U.S.A.* 99 8689–8694. 10.1073/pnas.122243399 12060707 PMC124360

[B64] Spalding M. H. (2008). Microalgal carbon-dioxide-concentrating mechanisms: *Chlamydomonas* inorganic carbon transporters. *J. Exp. Bot.* 59 1463–1473. 10.1093/jxb/erm128 17597098

[B65] Spicher L. Kessler F. (2015). Unexpected roles of plastoglobules (plastid lipid droplets) in vitamin K1 and E metabolism. *Curr. Opin. Plant Biol.* 25 123–129. 10.1016/j.pbi.2015.05.005 26037391

[B66] Sullivan S. Thomson C. E. Kaiserli E. Christie J. M. (2009). Interaction specificity of *Arabidopsis* 14-3-3 proteins with phototropin receptor kinases. *FEBS Lett.* 583 2187–2193. 10.1016/j.febslet.2009.06.011 19524572

[B67] Suzuki T. Yamasaki K. Fujita S. Oda K. Iseki M. Yoshida K. (2003). Archaeal-type rhodopsins in *Chlamydomonas*: model structure and intracellular localization. *Biochem. Biophys. Res. Commun.* 301 711–717. 10.1016/S0006-291X(02)03079-6 12565839

[B68] Takahashi T. Watanabe M. (1993). Photosynthesis modulates the sign of phototaxis of wild-type *Chlamydomonas reinhardtii*. Effects of red background illumination and 3-(3’,4’-dichlorophenyl)-1,1-dimethylurea. *FEBS Lett.* 336 516–520. 10.1016/0014-5793(93)80867-T 8282120

[B69] Trippens J. Greiner A. Schellwat J. Neukam M. Rottmann T. Lu Y. (2012). Phototropin influence on eyespot development and regulation of phototactic behavior in *Chlamydomonas reinhardtii*. *Plant Cell* 24 4687–4702. 10.1105/tpc.112.103523 23204408 PMC3531860

[B70] Veith T. Brauns J. Weisheit W. Mittag M. Büchel C. (2009). Identification of a specific fucoxanthin-chlorophyll protein in the light harvesting complex of photosystem I in the diatom *Cyclotella* meneghiniana. *Biochim. Biophys. Acta* 7 905–912. 10.1016/j.bbabio.2009.04.006 19397889

[B71] Vizcaino J. A. Cote R. G. Csordas A. Dianes J. A. Fabregat A. Foster J. M. (2013). The proteomics identifications (PRIDE) database and associated tools: status in 2013. *Nucleic Acids Res.* 41 D1063–D1069. 10.1093/nar/gks1262 23203882 PMC3531176

[B72] von Heinjne G. (1992). Membrane protein structure prediction: hydrophobicity analysis and the ‘positive inside’ rule. *J. Mol. Biol.* 225 487–494. 10.1016/0022-2836(92)90934-C 1593632

[B73] Vopalensky P. Pergner J. Liegertova M. Benito-Gutierrez E. Arendt D. Kozmik Z. (2012). Molecular analysis of the amphioxus frontal eye unravels the evolutionary origin of the retina and pigment cells of the vertebrate eye. *Proc. Natl. Acad. Sci. U.S.A.* 109 15383–15388. 10.1073/pnas.1207580109 22949670 PMC3458357

[B74] Wagner V. Ullmann K. Mollwo A. Kaminski M. Mittag M. Kreimer G. (2008). The phosphoproteome of a *Chlamydomonas reinhardtii* eyespot fraction includes key proteins of the light signaling pathway. *Plant Physiol.* 146 772–788. 10.1104/pp.107.109645 18065559 PMC2245826

[B75] Wang Z. Y. Shimonaga M. Kobayashi M. Nozawa T. (2002). N-terminal methylation of the core light-harvesting complex in purple photosynthetic bacteria. *FEBS Lett.* 519 164–168. 10.1016/S0014-5793(02)02744-8 12023037

[B76] Werner-Peterson R. Sloboda R. D. (2013). Methylation of structural components of the axoneme occurs during flagellar disassembly. *Biochemistry* 52 8501–8509. 10.1021/bi4011623 24152136

[B77] Wirschell M. Yamamoto R. Alford L. Gokhale A. Gaillard A. Sale W. S. (2011). Regulation of ciliary motility: conserved protein kinases and phosphatases are targeted and anchored in the ciliary axoneme. *Arch. Biochem. Biophys.* 510 93–100. 10.1016/j.abb.2011.04.003 21513695 PMC3114296

[B78] Witman G. B. (1993). *Chlamydomonas* phototaxis. *Trends Cell Biol.* 3 403–408. 10.1016/0962-8924(93)90091-E 14731659

[B79] Ytterberg A. J. Peltier J. van Wijk K. J. (2006). Protein profiling of plastoglobules in chloroplasts and chromoplasts. A surprising site for differential accumulation of metabolic enzymes. *Plant Physiol.* 140 984–997. 10.1104/pp.105.076083 16461379 PMC1400577

[B80] Zhang K. Yau P. M. Chandrasekhar B. New R. Kondrat R. Imai B. S. (2004). Differentiation between peptides containing acetylated or tri-methylated lysines by mass spectrometry: an application for determinin lysine 9 acetylation and methylation of histone H3. *Proteomics* 4 1–10. 10.1007/s12014-008-9020-1 14730666

