# Supplementary material for: Proteomic Analysis of a Fraction with Intact Eyespots of Chlamydomonas reinhardtii and Assignment of Protein Methylation
Source: Front Plant Sci. 2015 Dec 15;6:1085. doi: 10.3389/fpls.2015.01085 (PMC4678213; doi:10.3389/fpls.2015.01085)
Supplement: Supplementary file 1 [file Data_Sheet_1.PDF]

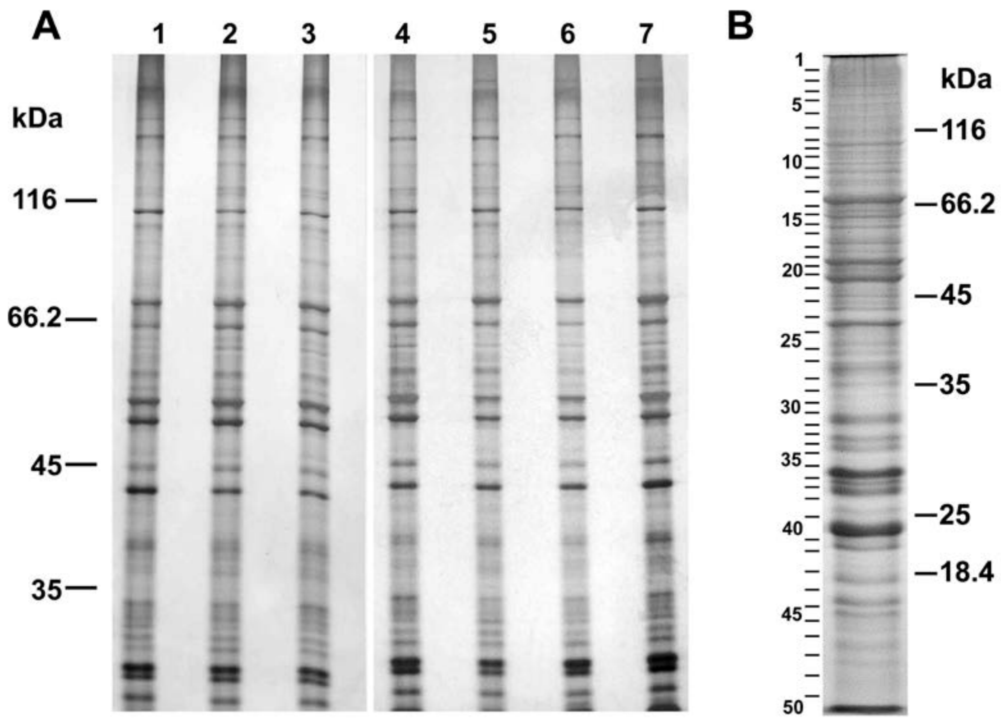

Supplemental Figure 1: The protein pattern of fraction F2A$_e$ is reproducible (A) and was cut into 50 slices for proteomic analysis (B). (A) Total proteins (7 µg) of independent isolations were separated by SDS-PAGE (9%) and stained with silver. **(B)** Proteins (~ 40 µg) from two independent eyespot isolations were combined and separated by 11% SDS-PAGE. Following gel staining with colloidal Coomassie, it was cut into 50 slices and subjected to in-gel trypsin digestion as described in Material and Methods.
