# Supplementary material for: Proteomic Analysis of a Fraction with Intact Eyespots of Chlamydomonas reinhardtii and Assignment of Protein Methylation
Source: Front Plant Sci. 2015 Dec 15;6:1085. doi: 10.3389/fpls.2015.01085 (PMC4678213; doi:10.3389/fpls.2015.01085)
Supplement: Supplementary file 2 [file Data_Sheet_2.PDF]

Supplemental Figure S2. (Peptides are listed in the order of Tables 2 and 3)

Transcript name (Phytozome *C. reinhardtii* database (Vs. 5.3.1)) or chloroplast (Cp) genome database	Function and/or homologies	Methylated peptide	z	Xcorr	x-times found
**Proteins important for eyespot development**					
Cre16.g666550.t1.2	Soul3	QRQAFIMNDTCRmFLATDLKm[2]	3	3,8	2

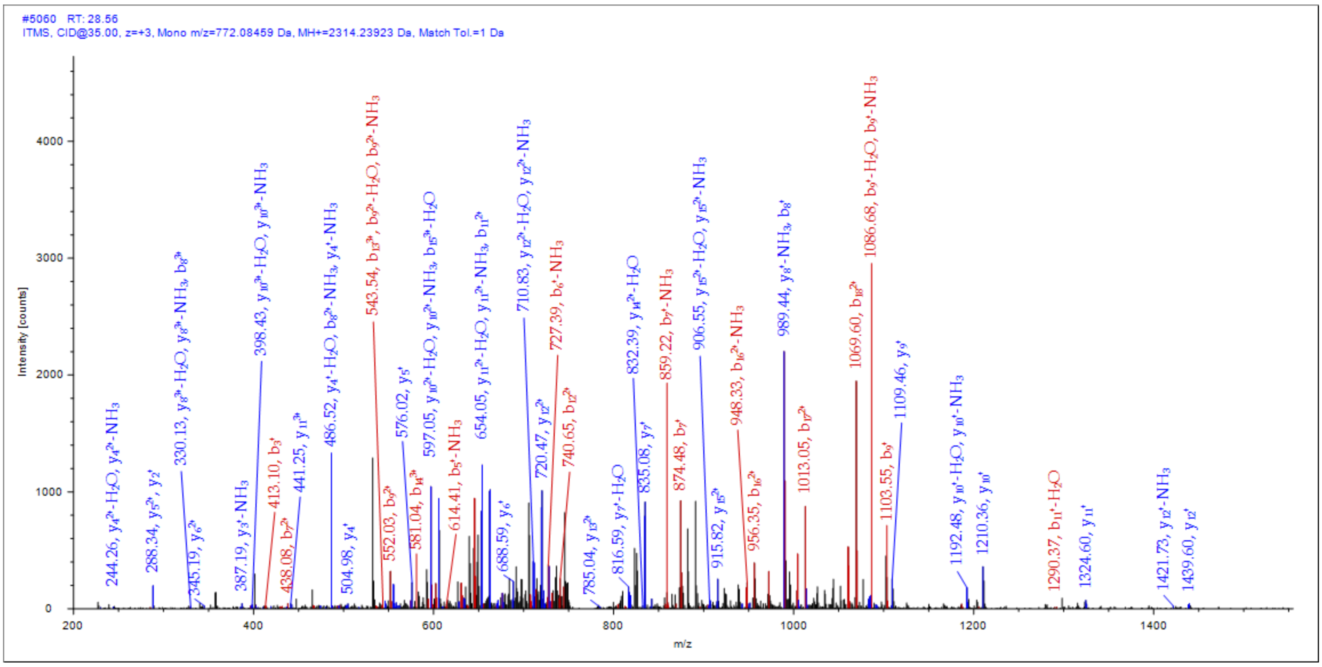

Cre16.g666550.t1.2	Soul3	->QRQAFIMoNDTCRmFLATDLKm[2] [b]	3	3.51	1

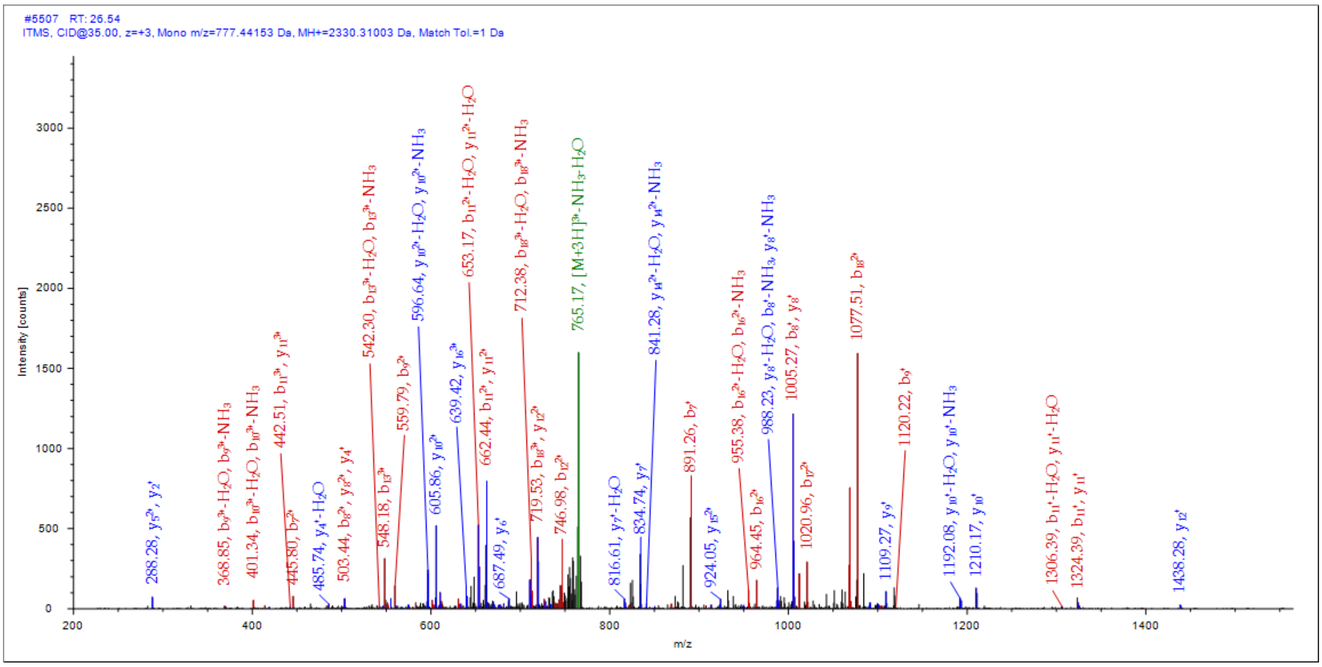

Transcript name (Phytozome *C. reinhardtii* database (Vs. 5.3.1)) or chloroplast (Cp) genome database	Function and/or homologies	Methylated peptide	z	Xcorr	x-times found
Cre12.g509250.t1.1	EYE2, no eyespot	LTDDELIALVNSDPDLDKm[b]	2	4.40	1

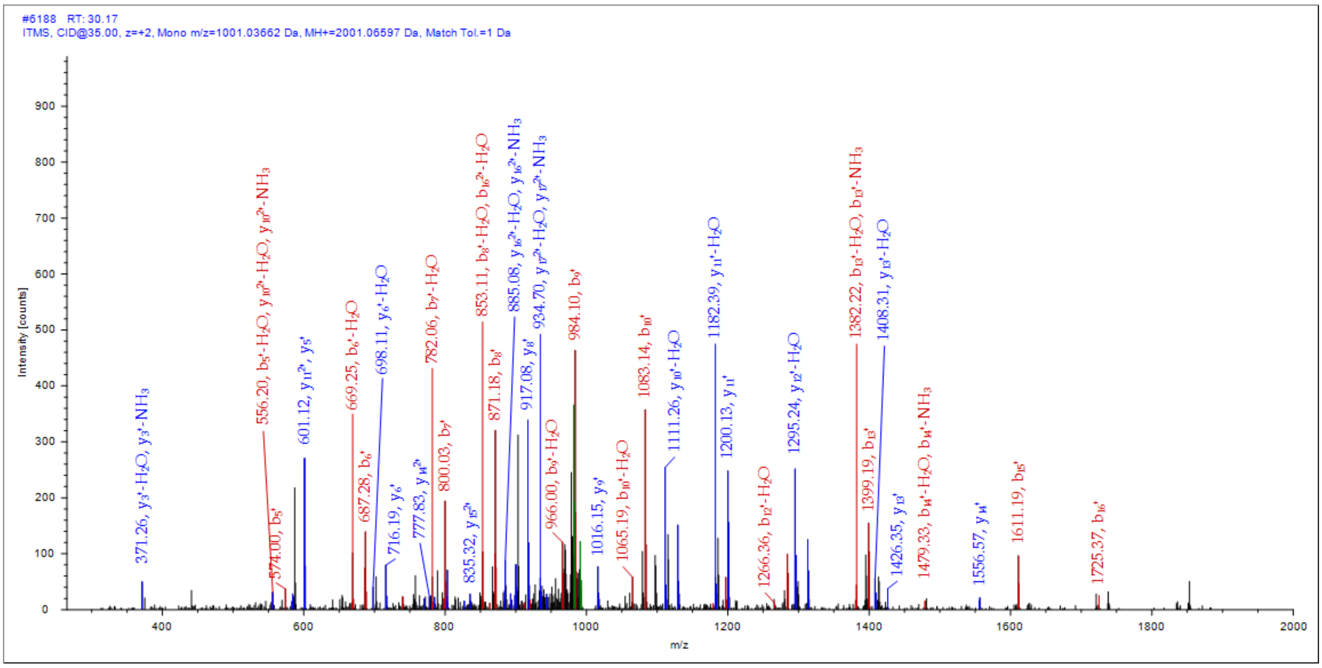

14-3-3 proteins

Transcript name	Function and/or homologies	Methylated peptide	z	Xcorr	x-times found
Cre12.g559250.t1.2	14-3-3 protein	DNLTLWTSDMoQDPAAGDDRmEGADMo Km^{2}VEDAEP[a]	3	4.37	2

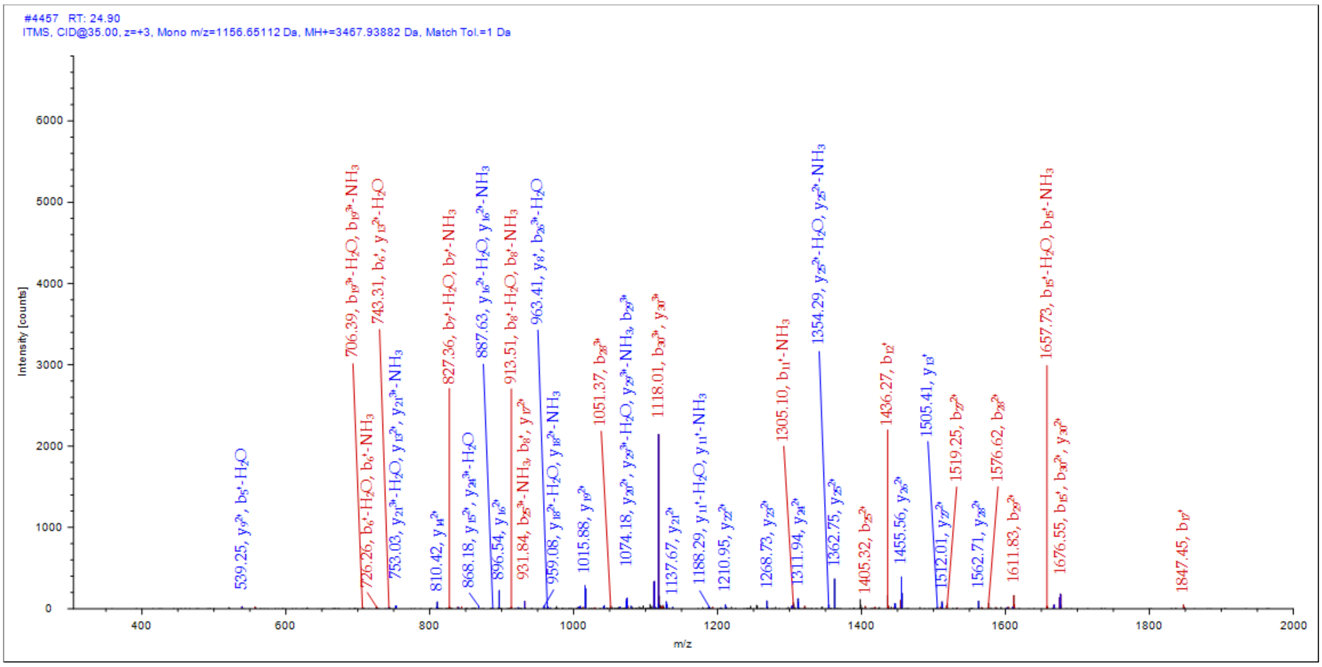

Transcript name (Phytozome *C. reinhardtii* database (Vs. 5.3.1)) or chloroplast (Cp) genome database	Function and/or homologies	Methylated peptide	z	Xcorr	x-times found
**Photosynthesis / electron transport / light harvesting**					
Cp genome	atpB, ATP synthase subunit beta	FLSQPFFVAEVFTGSPGKm^{2}YVSLAETIEG FGK[a,b]	3	5.97	6

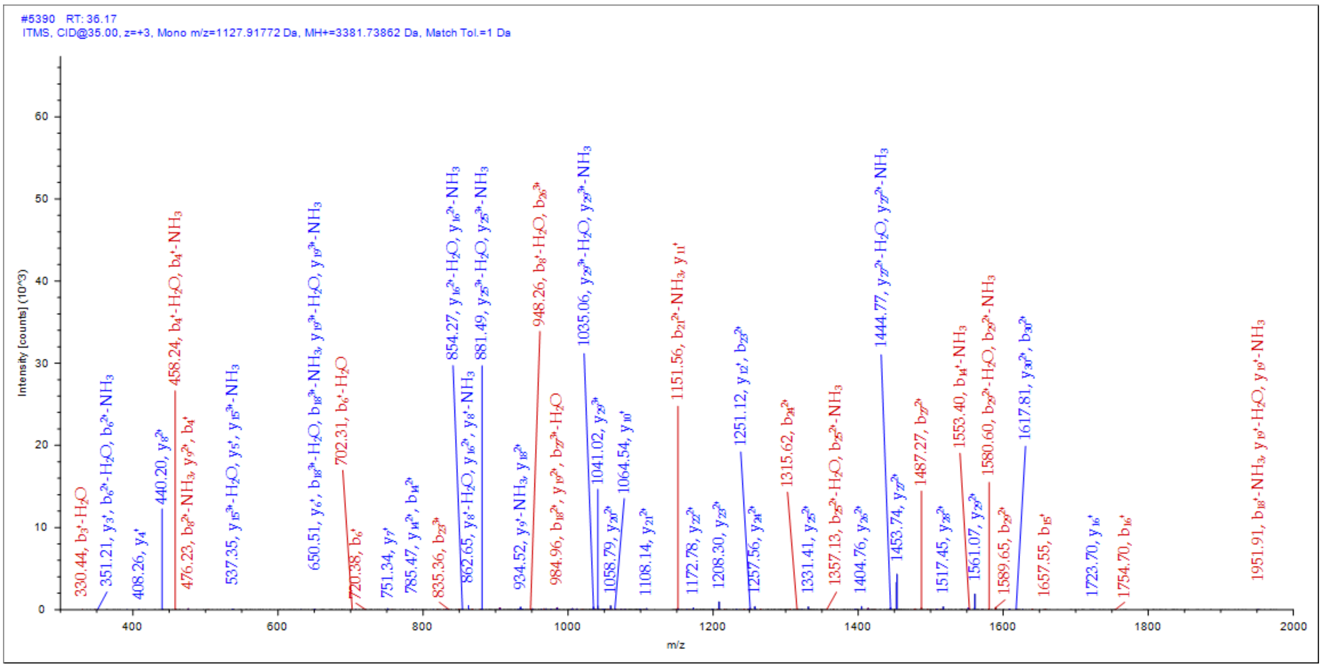

Cp genome	atpB, ATP synthase subunit beta	->FLSQPFFVAEVFTGSPGKmYVSLAETIE GFGKm[a]	3	3.81	1

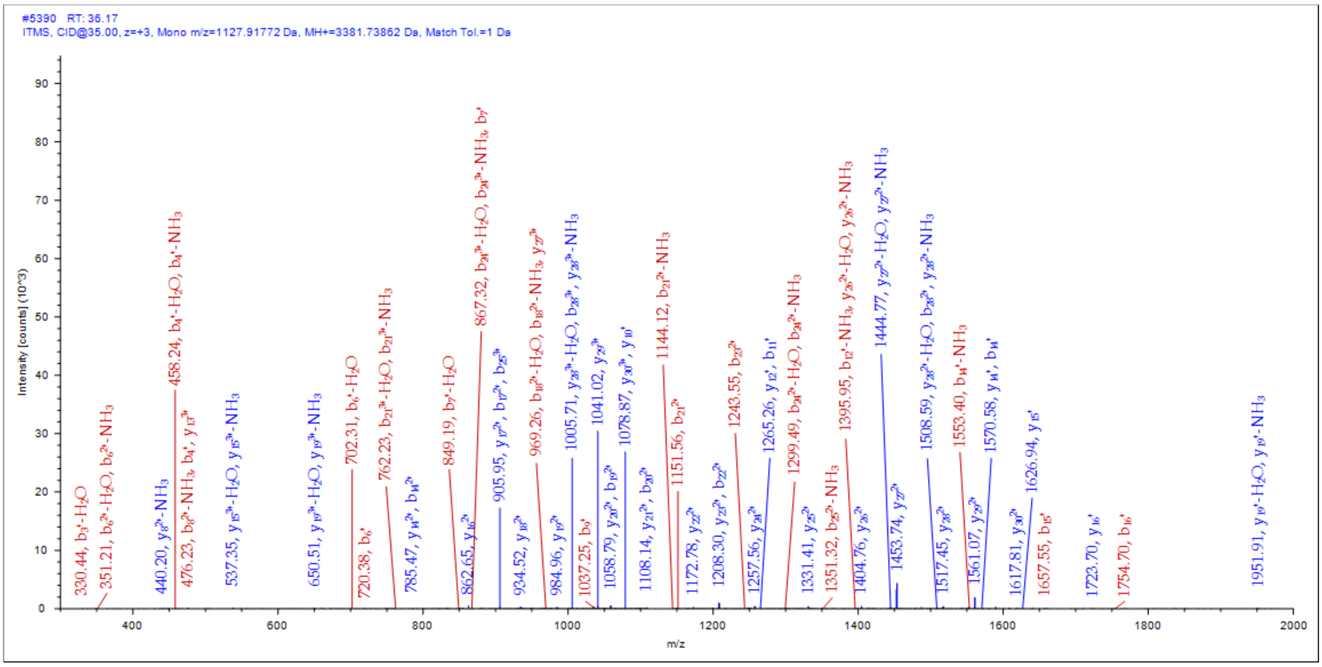

Transcript name (Phytozome *C. reinhardtii* database (Vs. 5.3.1)) or chloroplast (Cp) genome database	Function and/or homologies	Methylated peptide	z	Xcorr	x-times found
Cp genome	atpB, ATP synthase subunit beta	ELQDIIAILGLDELSEEDRm[b]	2	3.51	2

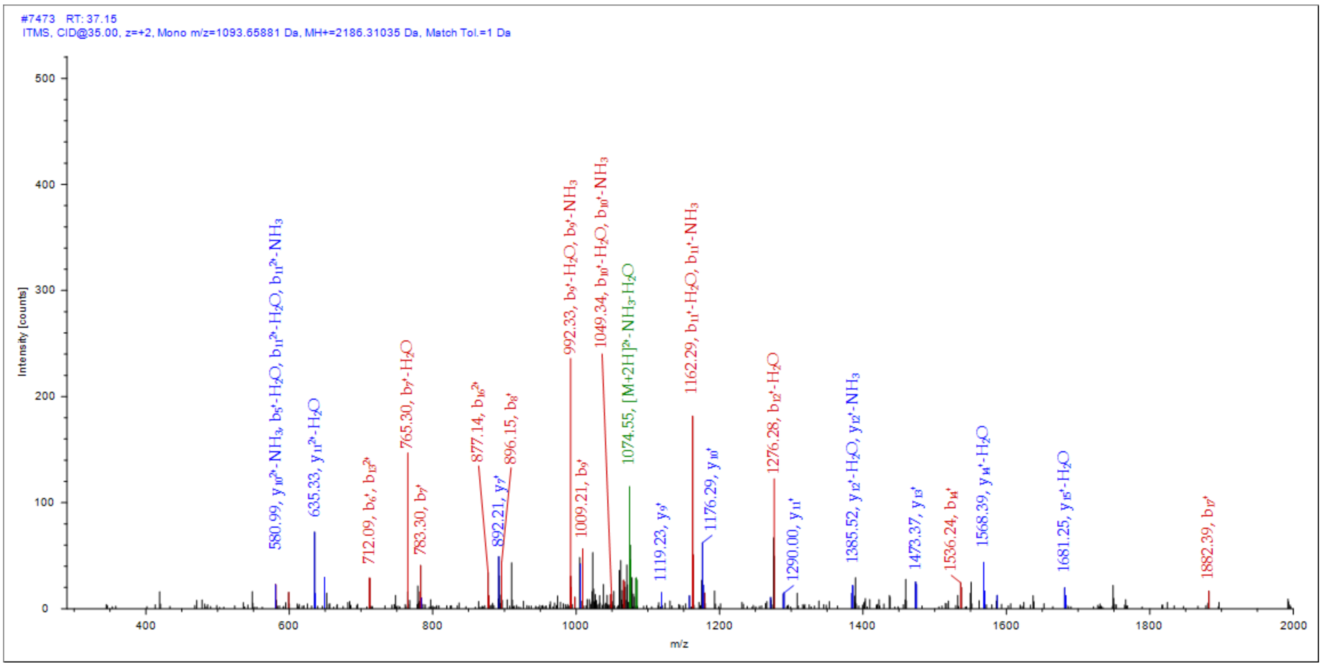

Cp genome	atpB, ATP synthase subunit beta	GMEVVDTGKm[2]PLSVPVGK[a]	2	2.91	1

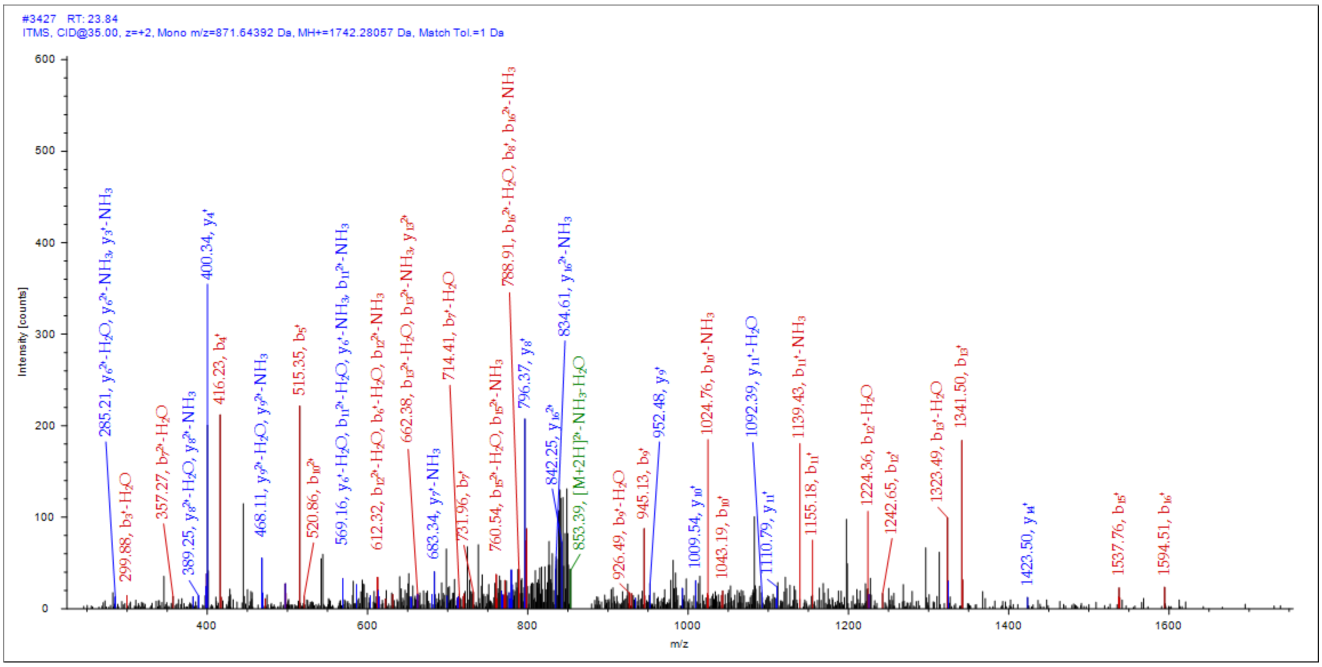

**Transcript name (Phytozome *C. reinhardtii* database (Vs. 5.3.1)) or chloroplast (Cp) genome database**	**Function and/or homologies**	**Methylated peptide**	**z**	**Xcorr**	**x-times found**
Cp genome	atpB, ATP synthase subunit beta	->GMoEVVDTGKPLSVPVGKm[b]	2	3.09	1

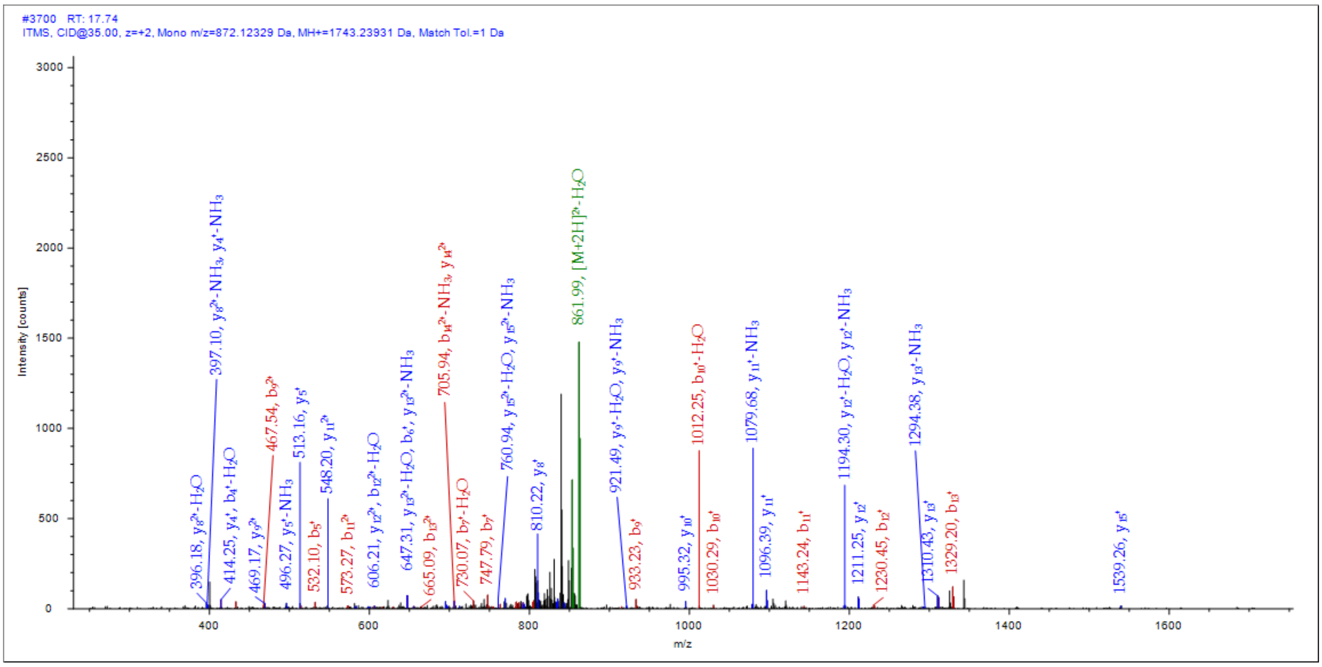

Cp genome	atpB, ATP synthase subunit beta	->GMoEVVDTGKmPLSVPVGK[b]	2	2.81	1

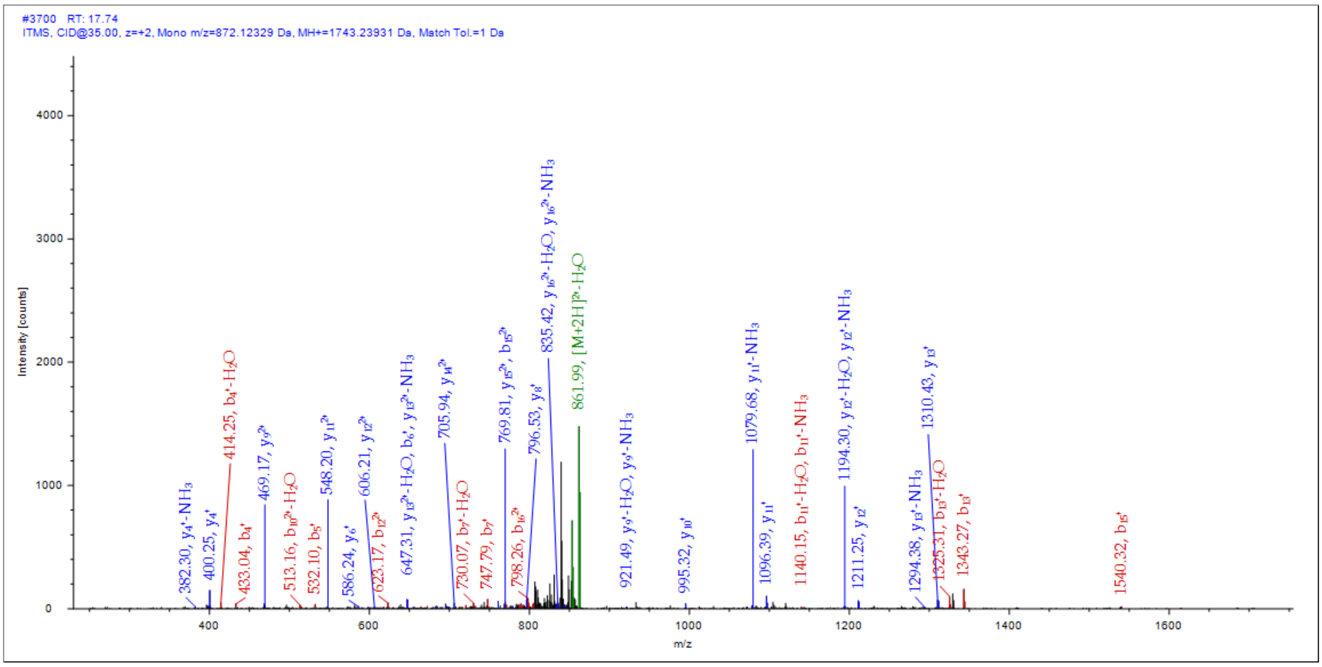

Transcript name (Phytozome *C. reinhardtii* database (Vs. 5.3.1)) or chloroplast (Cp) genome database	Function and/or homologies	Methylated peptide	z	Xcorr	x-times found
Cp genome	atpB, ATP synthase subunit beta	TVLIMoELINNIAKm[b]	2	2.73	1

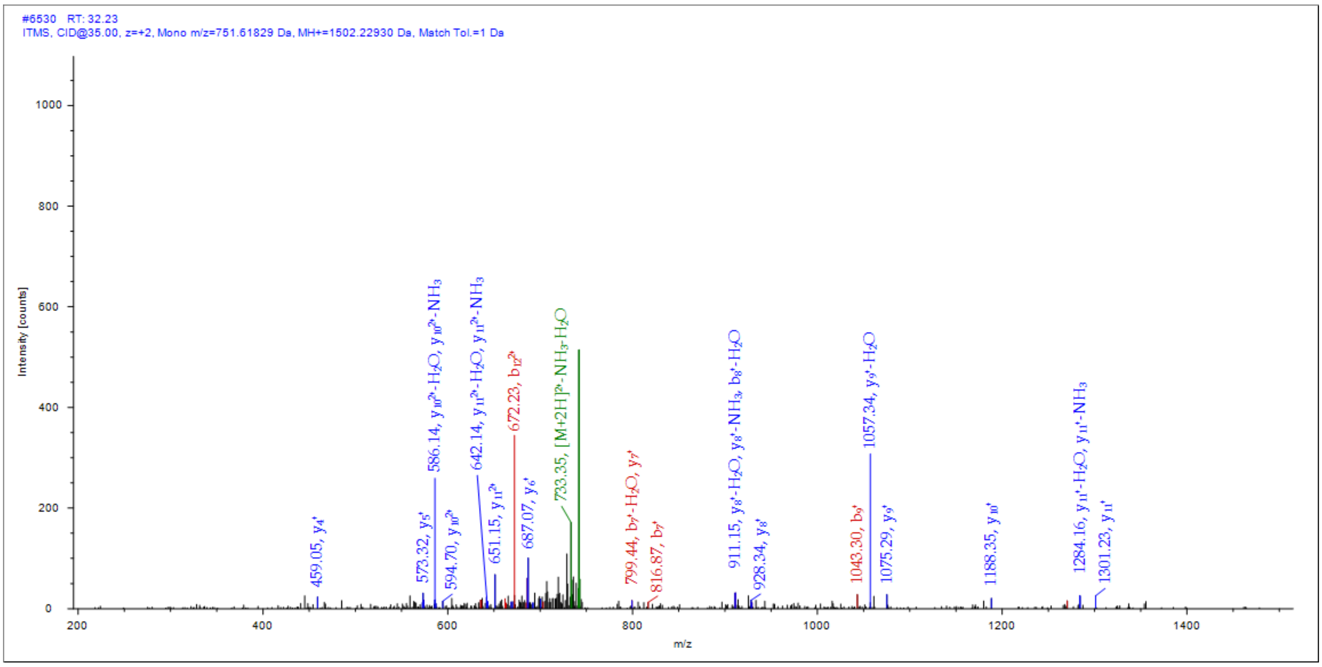

Cp genome	atpA, ATP synthase subunit alpha	SYLANSYPKm^{2}YGEILR[a]	2	2.93	1

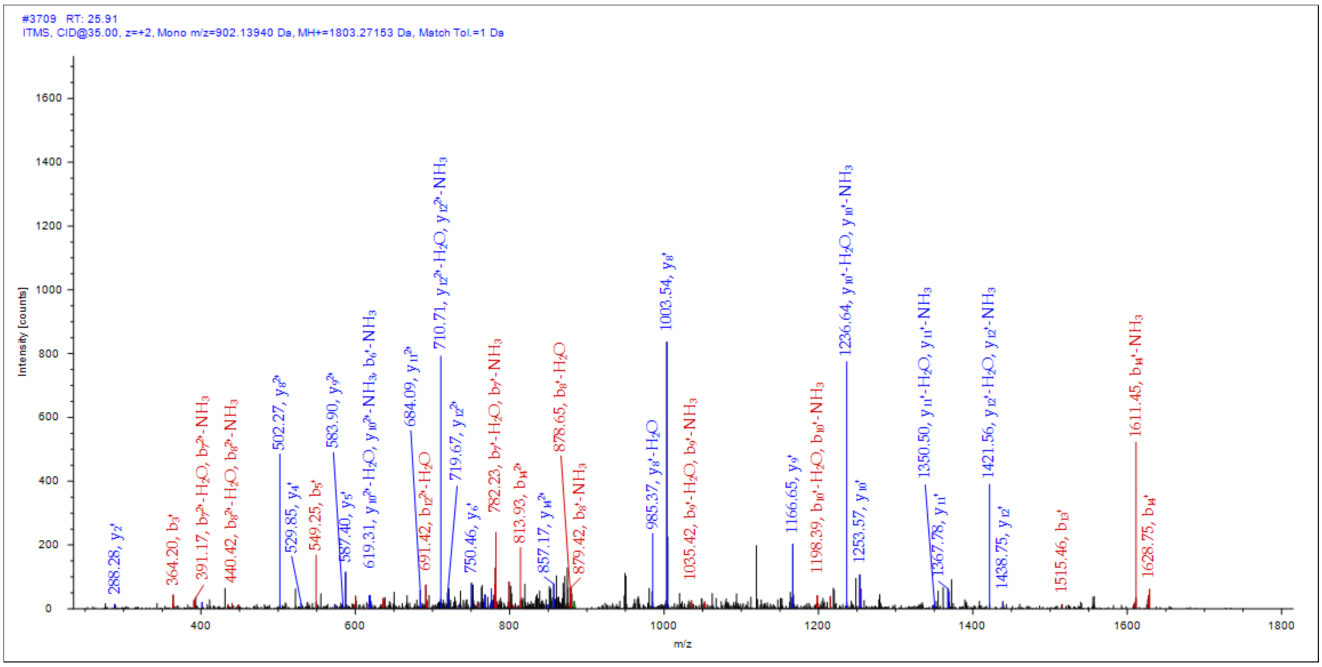

Transcript name (Phytozome *C. reinhardtii* database (Vs. 5.3.1)) or chloroplast (Cp) genome database	Function and/or homologies	Methylated peptide	z	Xcorr	x-times found
Cp genome	atpA, ATP synthase subunit alpha	SVYEPLATGLVAVDAMoIPVGRm[b]	2	3.38	1

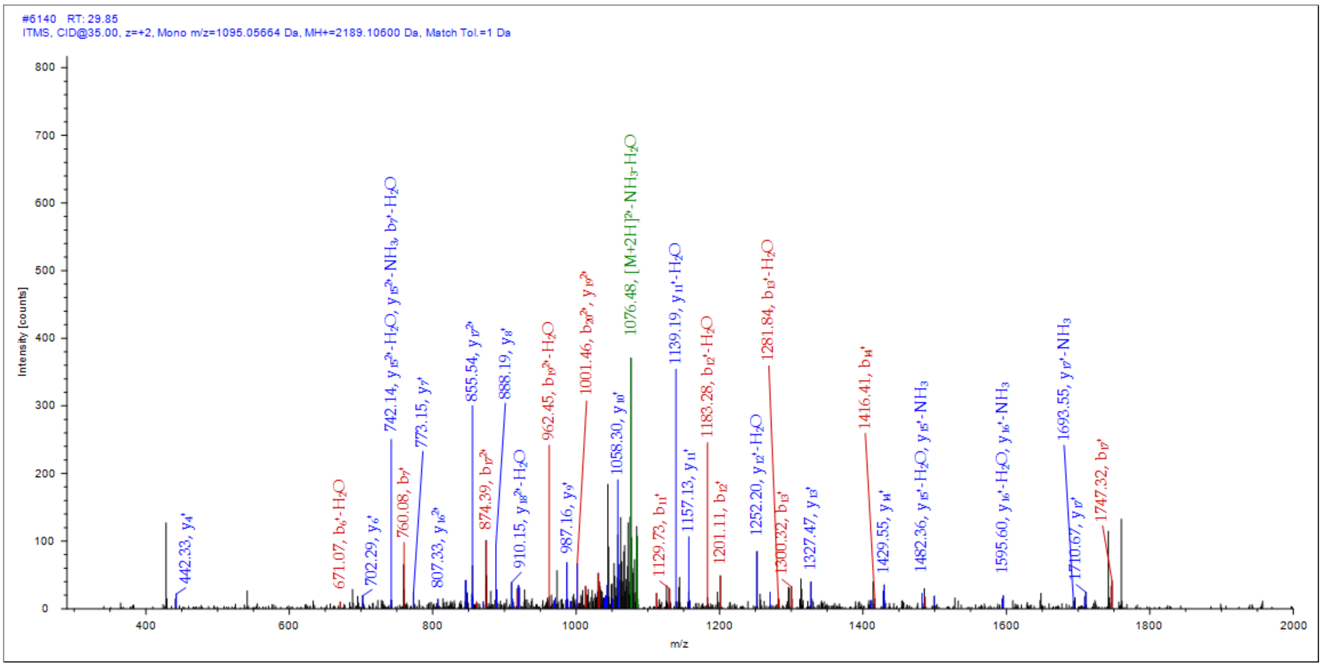

Cp genome	atpI, CF0 ATP synthase subunit I	YVEPAAFLLPINVLEDFTKm^{3}PLSLSFR[a]	3	3.11	1

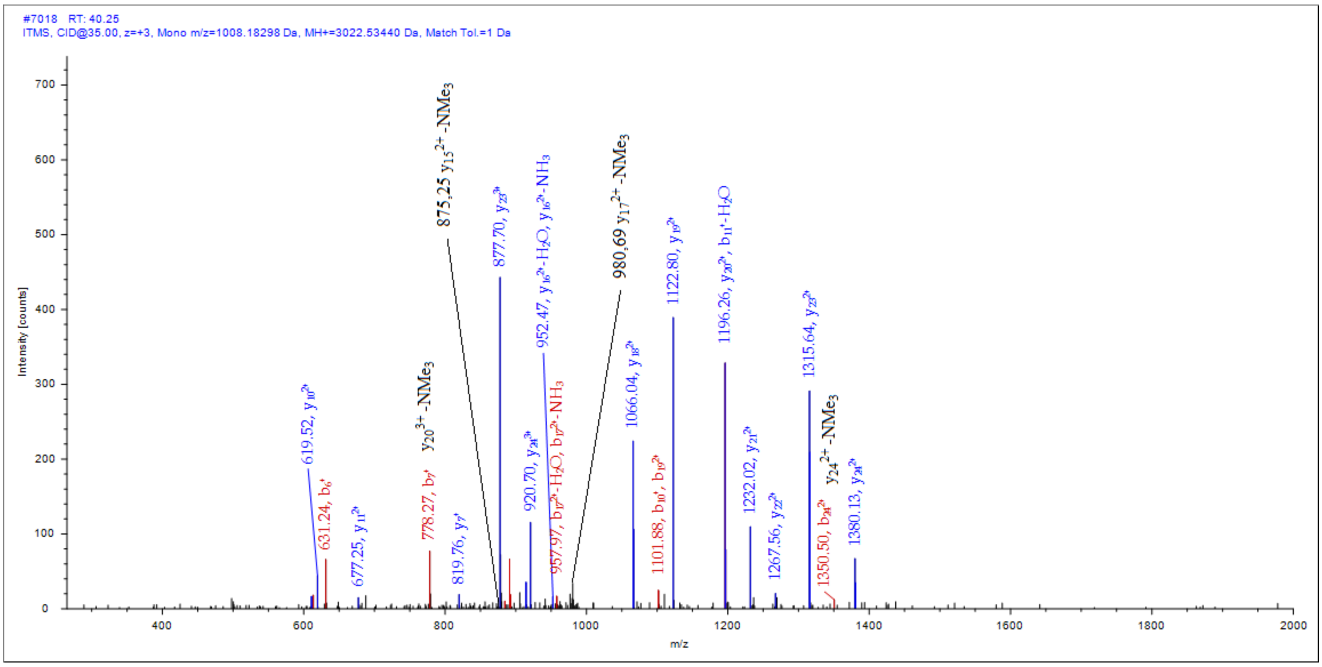

Transcript name (Phytozome *C. reinhardtii* database (Vs. 5.3.1)) or chloroplast (Cp) genome database	Function and/or homologies	Methylated peptide	z	Xcorr	x-times found
Cre06.g261000.t1.2	10 kDa PS II polypeptide	GKm^{2}GYGVYR[b]	1	2.26	2

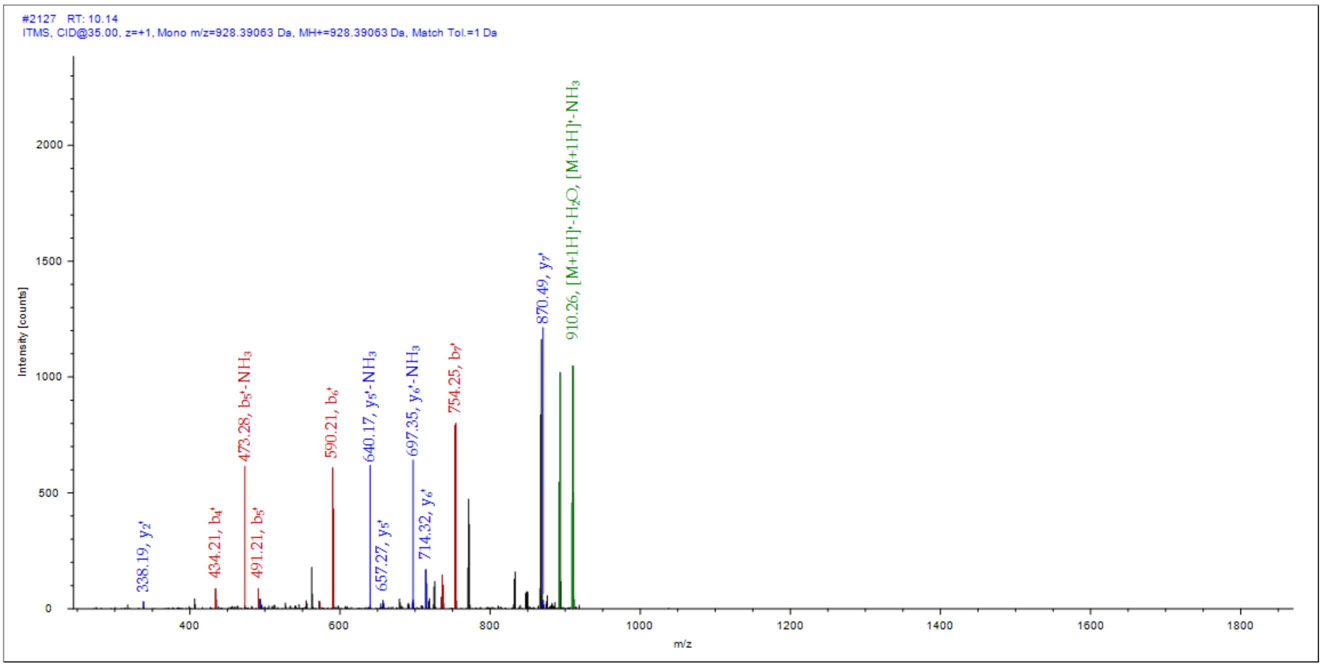

Cre06.g261000.t1.2	10 kDa PS II polypeptide	YEDKmYGANVDGYSPIYTPDLWTESGDS YTLGTK[a]	3	7.16	1

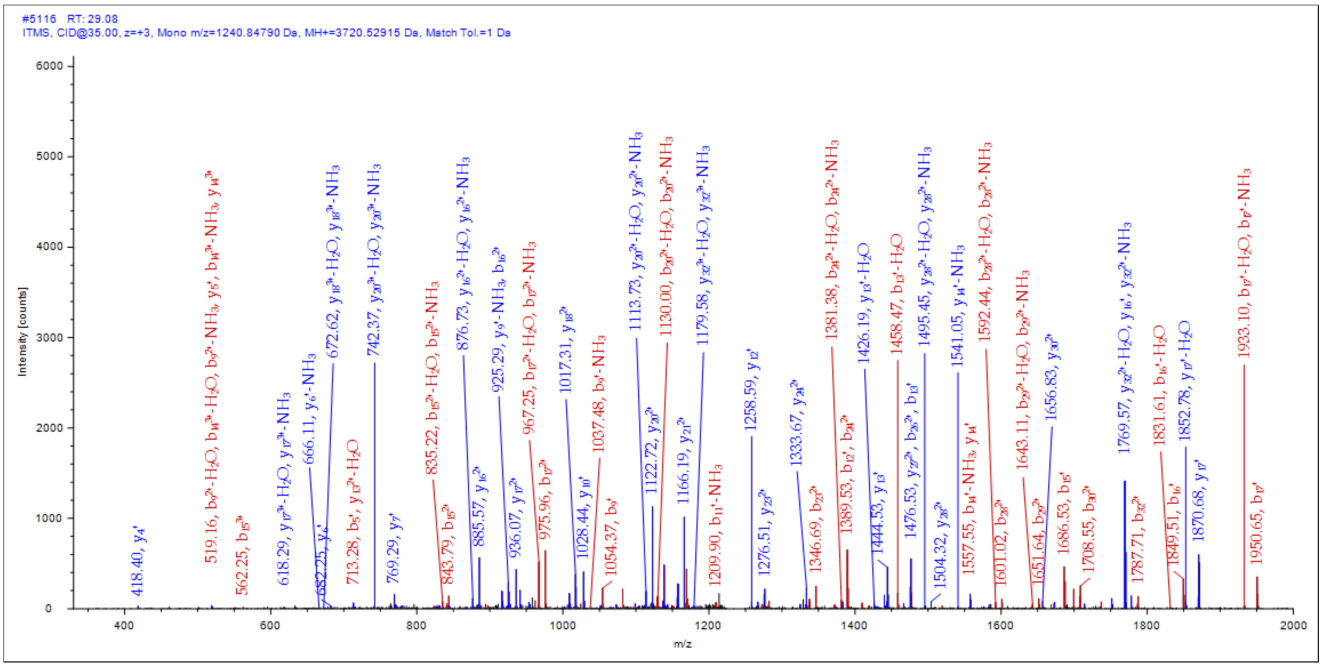

Transcript name (Phytozome *C. reinhardtii* database (Vs. 5.3.1)) or chloroplast (Cp) genome database	Function and/or homologies	Methylated peptide	z	Xcorr	x-times found
Cp genome	petA, Cytochrom f	KmYSEMVVPILSPDPAKm[b]	2	2.60	1

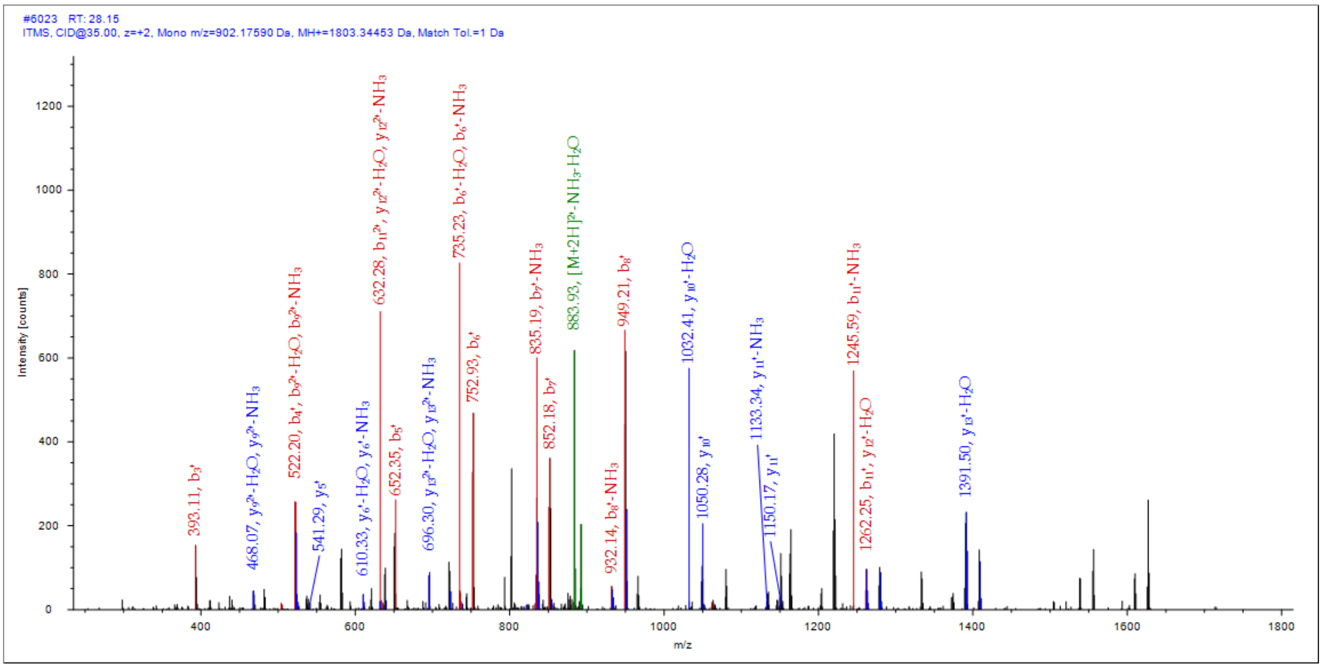

Cp genome	petA, Cytochrom f	->YSEMoVVPILSPDPAKm[b]	2	2.60	1

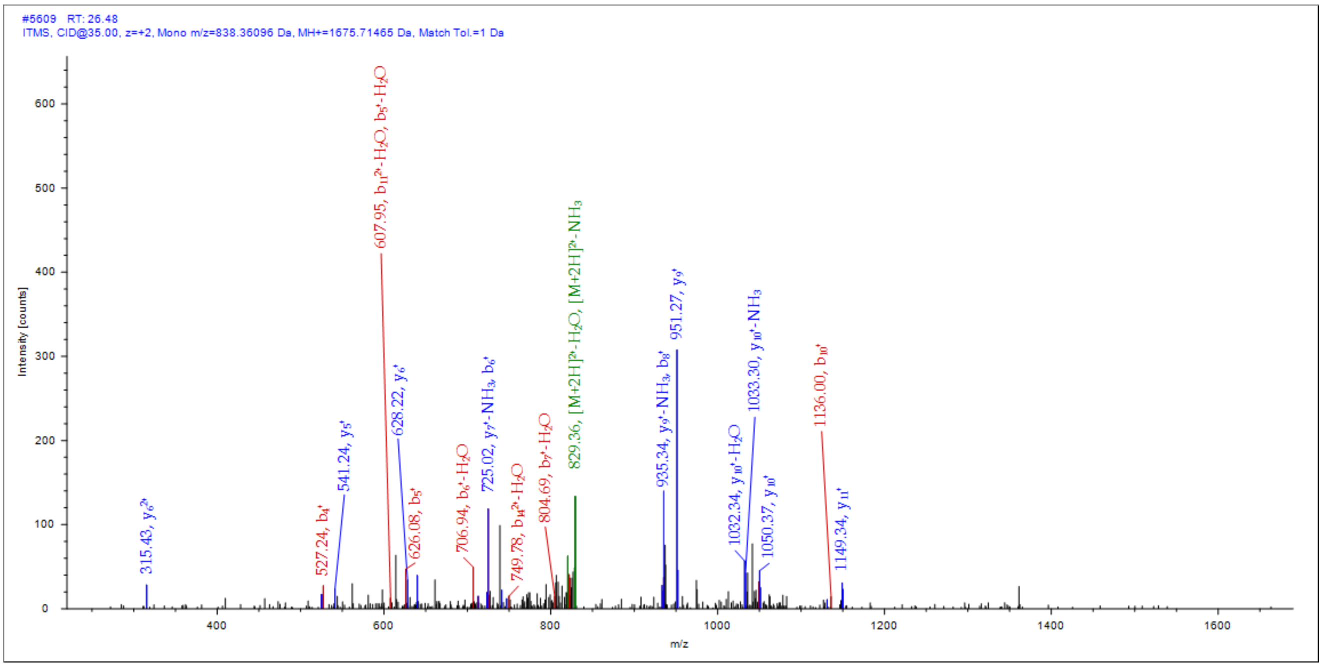

Transcript name (Phytozome *C. reinhardtii* database (Vs. 5.3.1)) or chloroplast (Cp) genome database	Function and/or homologies	Methylated peptide	z	Xcorr	x-times found
Cre16.g687900.t1.2	Lhca7, light-harvesting protein of PS I	NPGSQADGSFLGFTEEFKm[a]	2	3.48	5

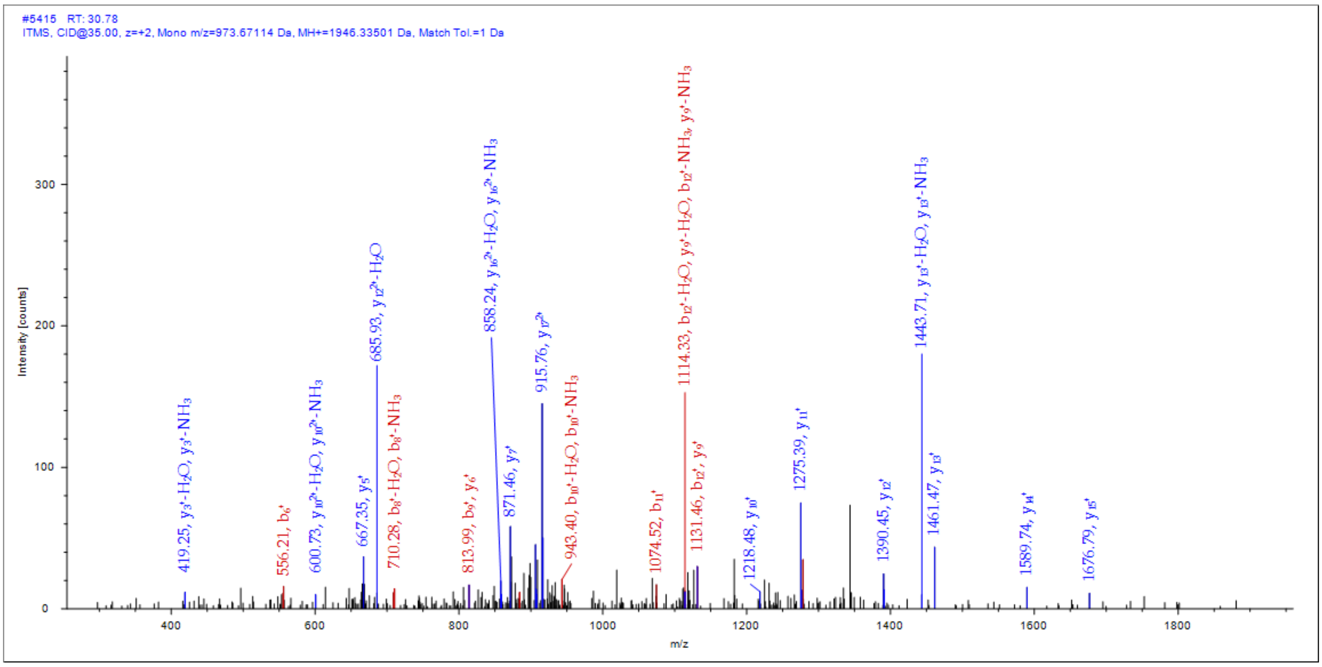

Cp genome	psbD, PS II D2 protein	AAEDPEFETFYTKm[a]	2	3.40	1

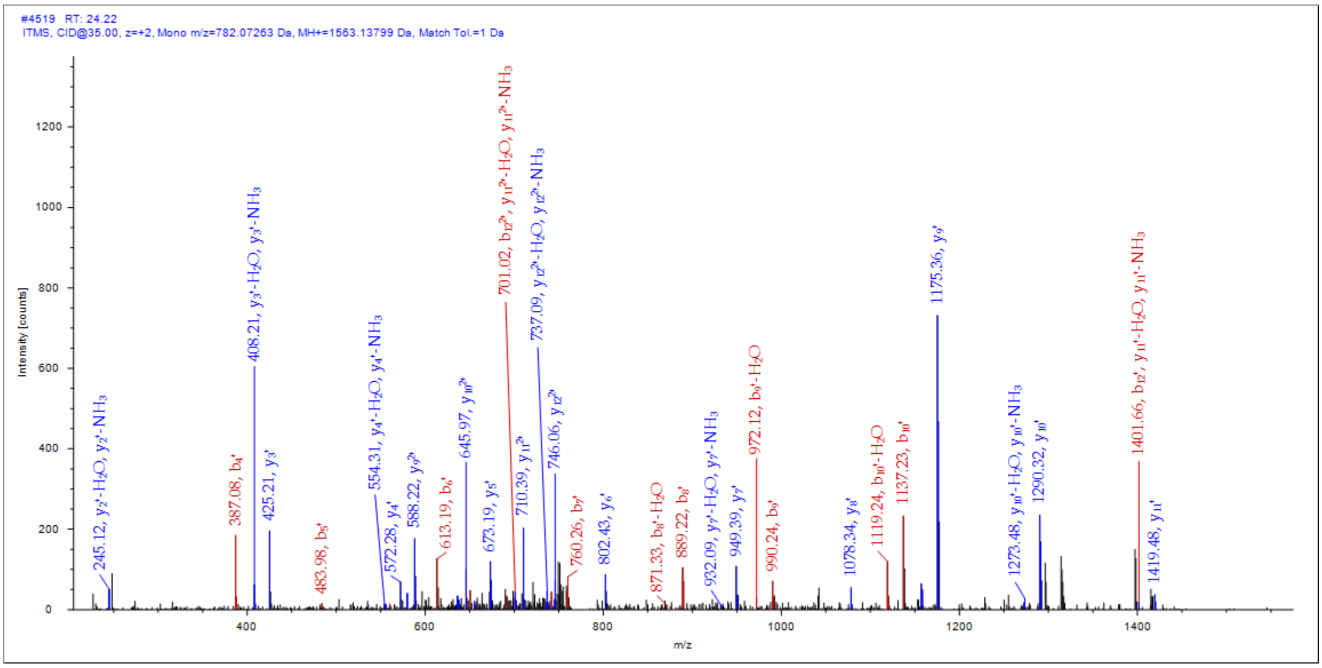

Transcript name (Phytozome *C. reinhardtii* database (Vs. 5.3.1)) or chloroplast (Cp) genome database	Function and/or homologies	Methylated peptide	z	Xcorr	x-times found
Cp genome	psaB, PS I P700 chlorophyll a apoprotein A2	GYWQELIETLVWAHEKmTPLANLVY WKm[a]	3	3.40	1

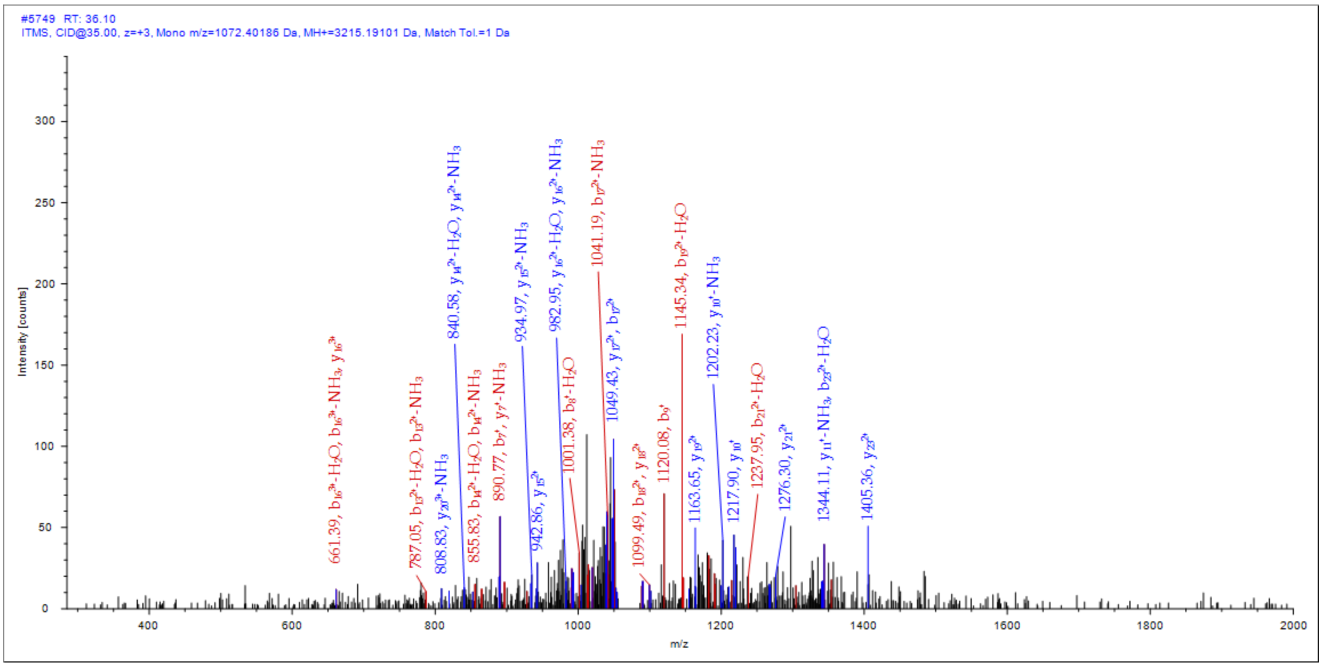

Cp genome	ycf4, PS I assembly protein	EIEKmQASELANFLQVSLEA[b]	2	2.77	1

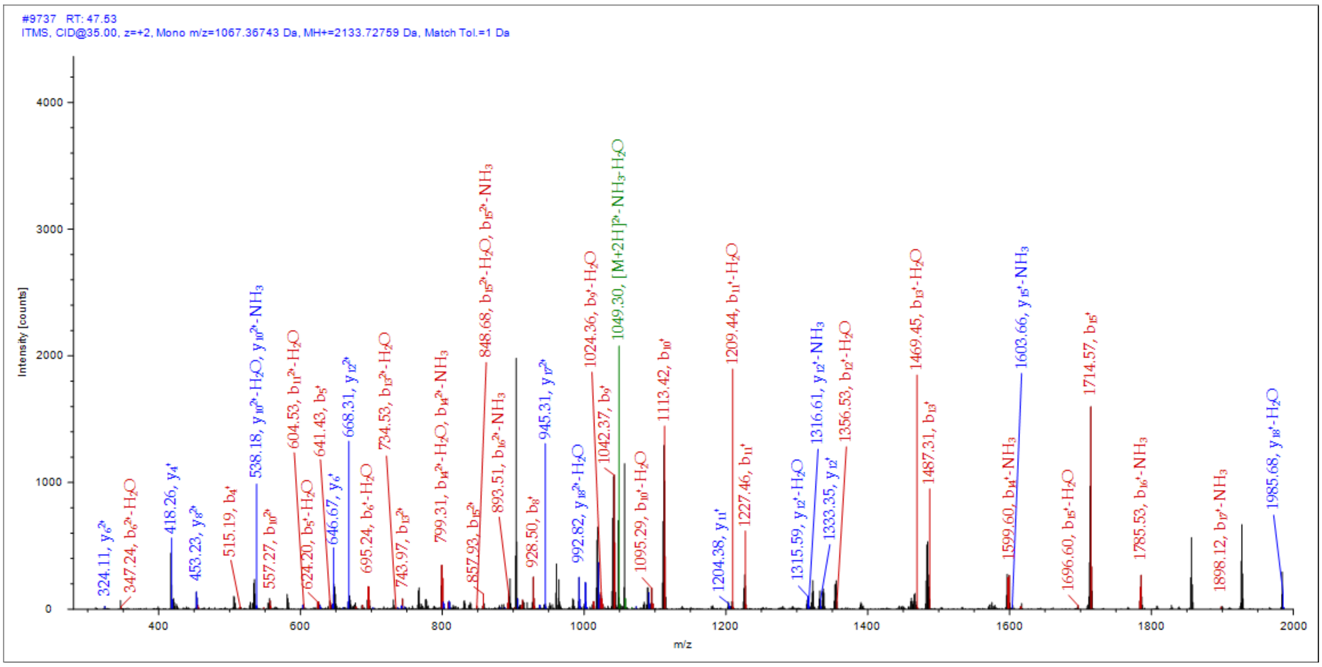

Transcript name (Phytozome *C. reinhardtii* database (Vs. 5.3.1)) or chloroplast (Cp) genome database	Function and/or homologies	Methylated peptide	z	Xcorr	x-times found
**Transporter**					
Cp genome	cemA (ycf10), inner envelope protein	FLKm^{3}QLFSDVDNLVIQEYR[a]	3	3.10	1

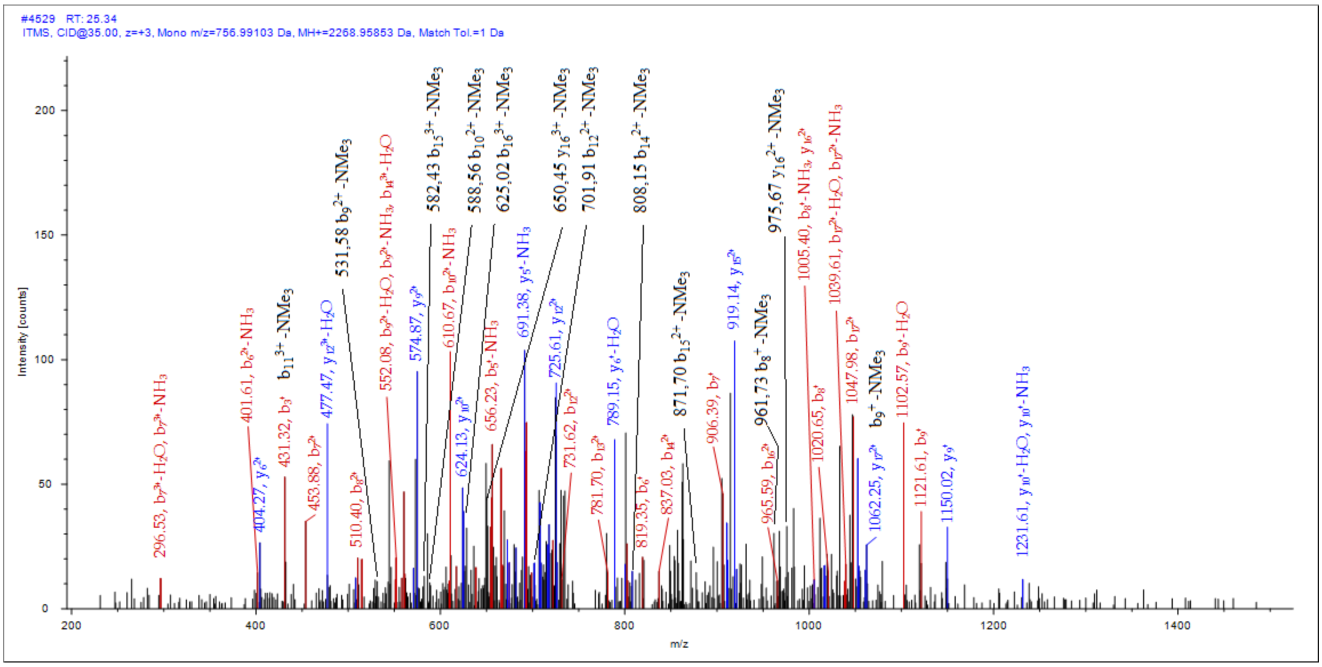

Cp genome	cemA (ycf10), inner envelope protein	GSLDSIKmNKm^{3}DISK[a]	3	3.00	1

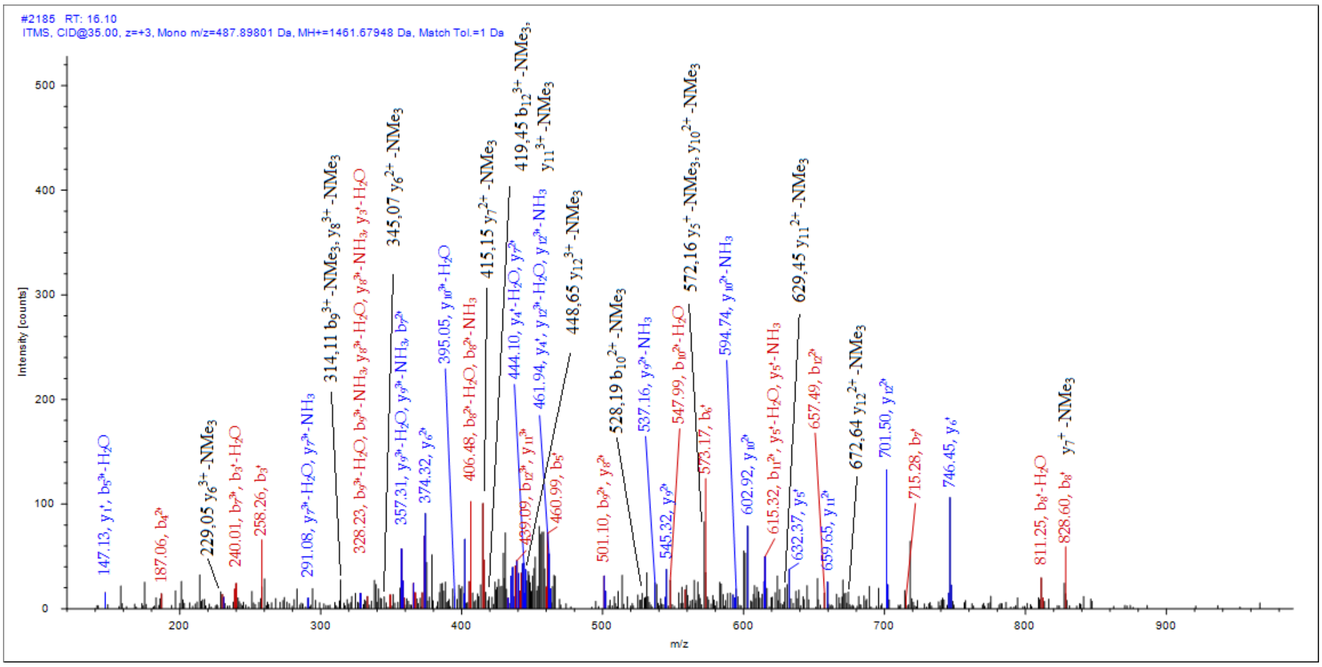

Transcript name (Phytozome *C. reinhardtii* database (Vs. 5.3.1)) or chloroplast (Cp) genome database	Function and/or homologies	Methylated peptide	z	Xcorr	x-times found
g11711.t1	Similar to ATPase components of ABC transporters	LQTTKIGMLSEGQKm^{2}SR[a]	2	3.28	1

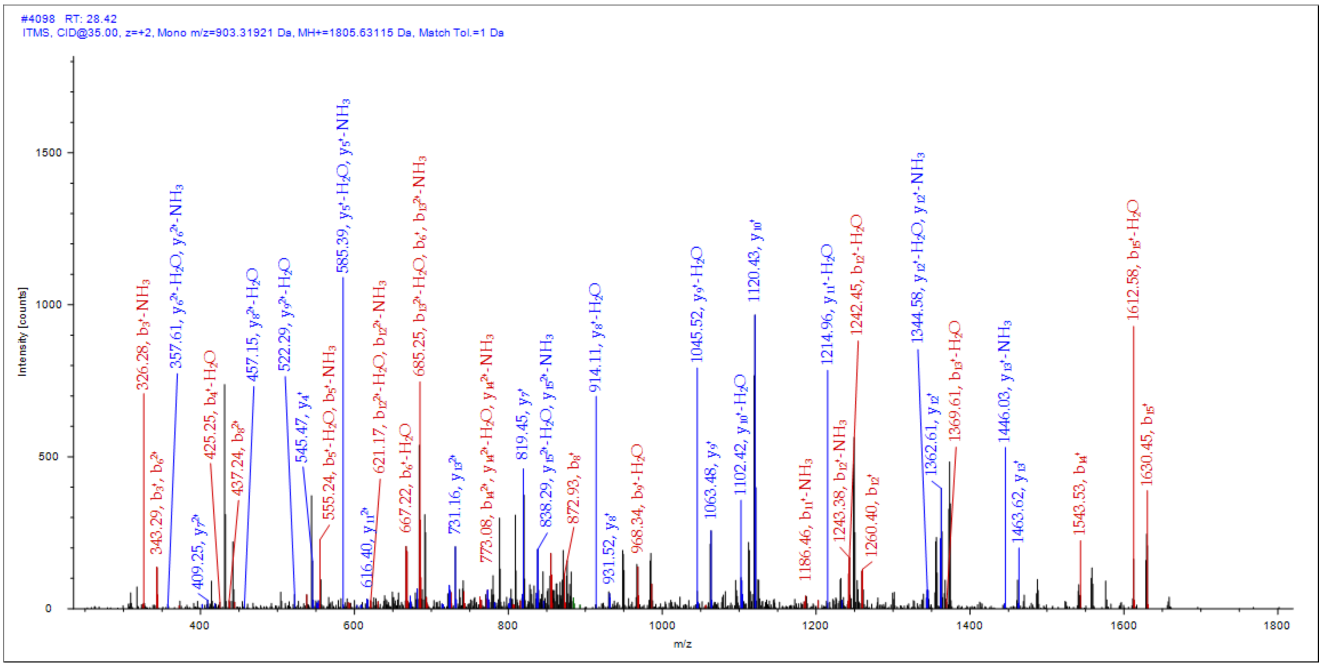

Ferredoxin and thioredoxin-related proteins

Cre11.g476750.t1.2	Ferredoxin-NADP reductase	KmGLCSNFLCDATPGTEISMoTGPTGK[a]	2	3.61	1

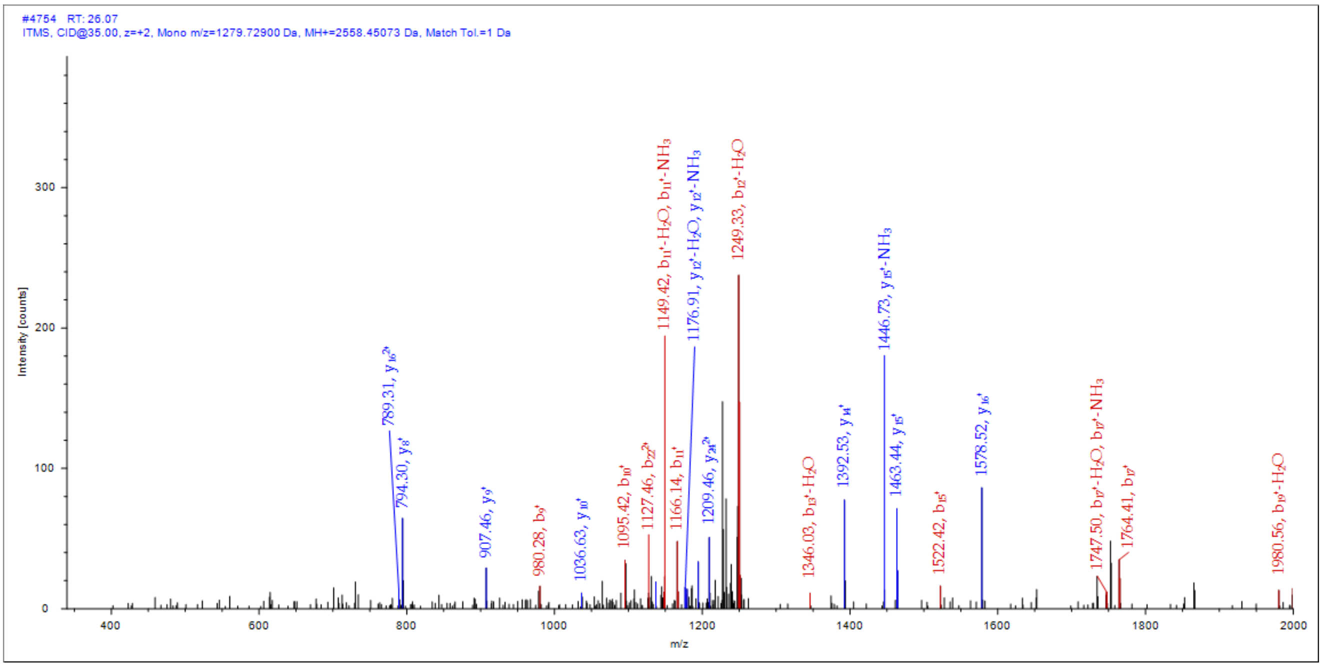

Transcript name (Phytozome *C. reinhardtii* database (Vs. 5.3.1)) or chloroplast (Cp) genome database	Function and/or homologies	Methylated peptide	z	Xcorr	x-times found
Cre11.g476750.t1.2	Ferredoxin-NADP reductase	->KmGLCSNFLCDATPGTEISMTGPTGK[a]	2	3.42	1

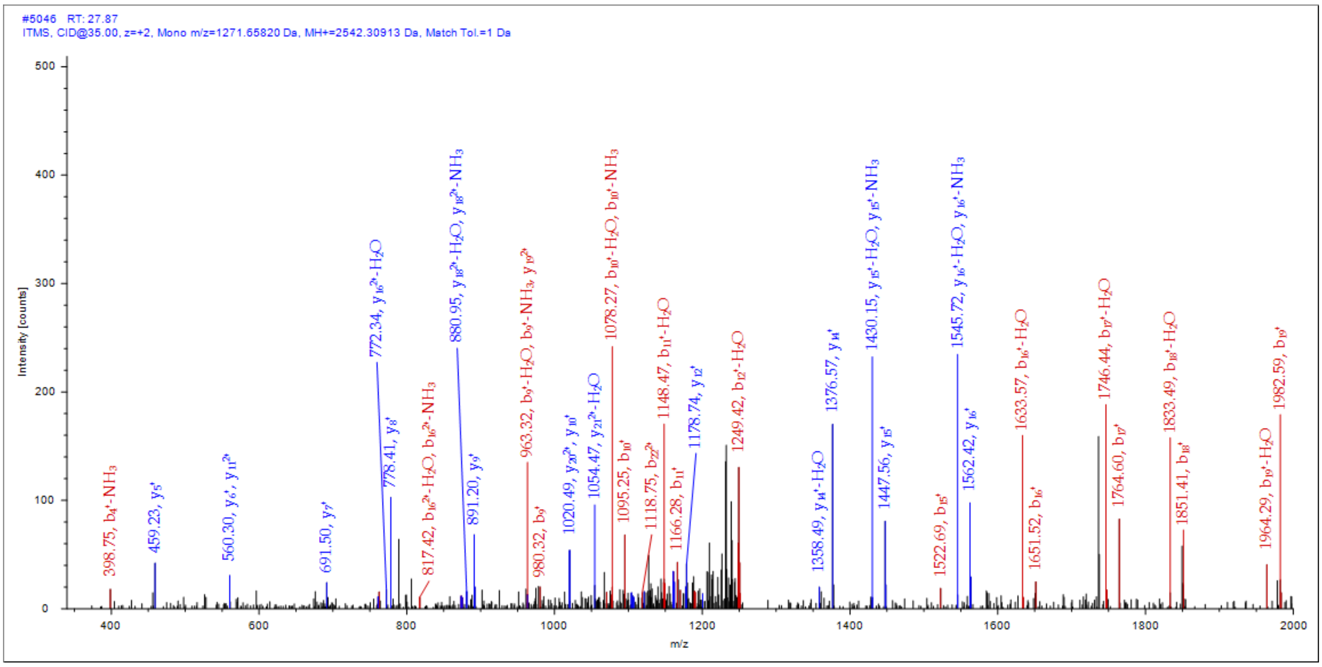

Cre11.g476750.t1.2	Ferredoxin-NADP reductase	IPFWEGQSYGVIPPGTKmINSKm[2 a]	3	3.82	1

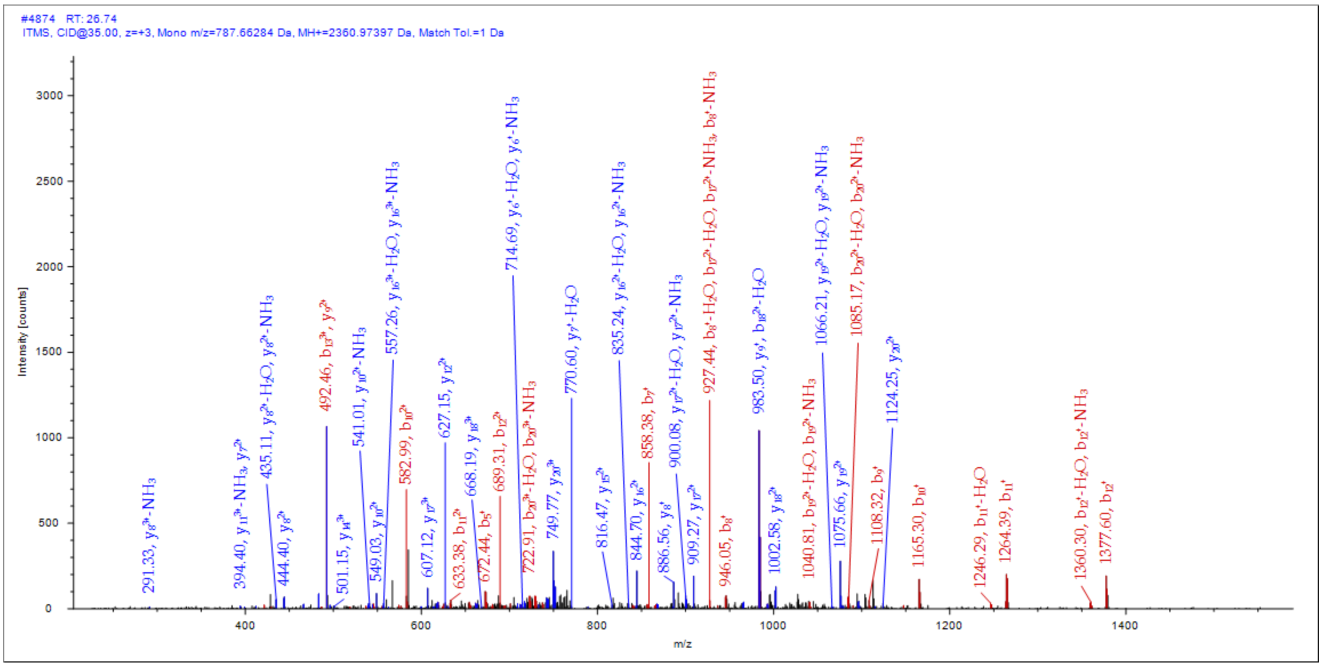

Transcript name (Phytozome *C. reinhardtii* database (Vs. 5.3.1)) or chloroplast (Cp) genome database	Function and/or homologies	Methylated peptide	z	Xcorr	x-times found
Cre11.g476750.t1.2	Ferredoxin-NADP reductase	->IPFWEGQSYGVIPPGTKINSKm[3 a]	3	3.84	1

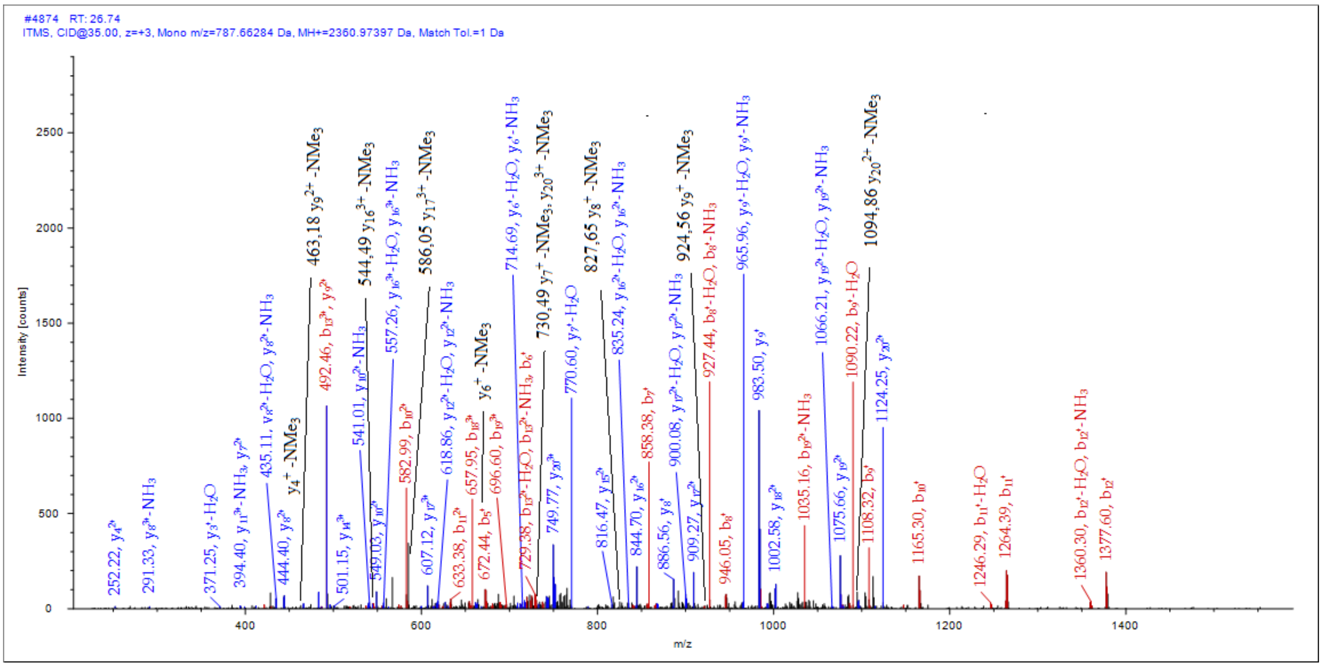

(Lipid) Metabolism

g11946.t1, g11946.t2	Similar to Cytochrome b5 reductase	APDYSQGEVSGLLKm[2 a]	2	3.32	3

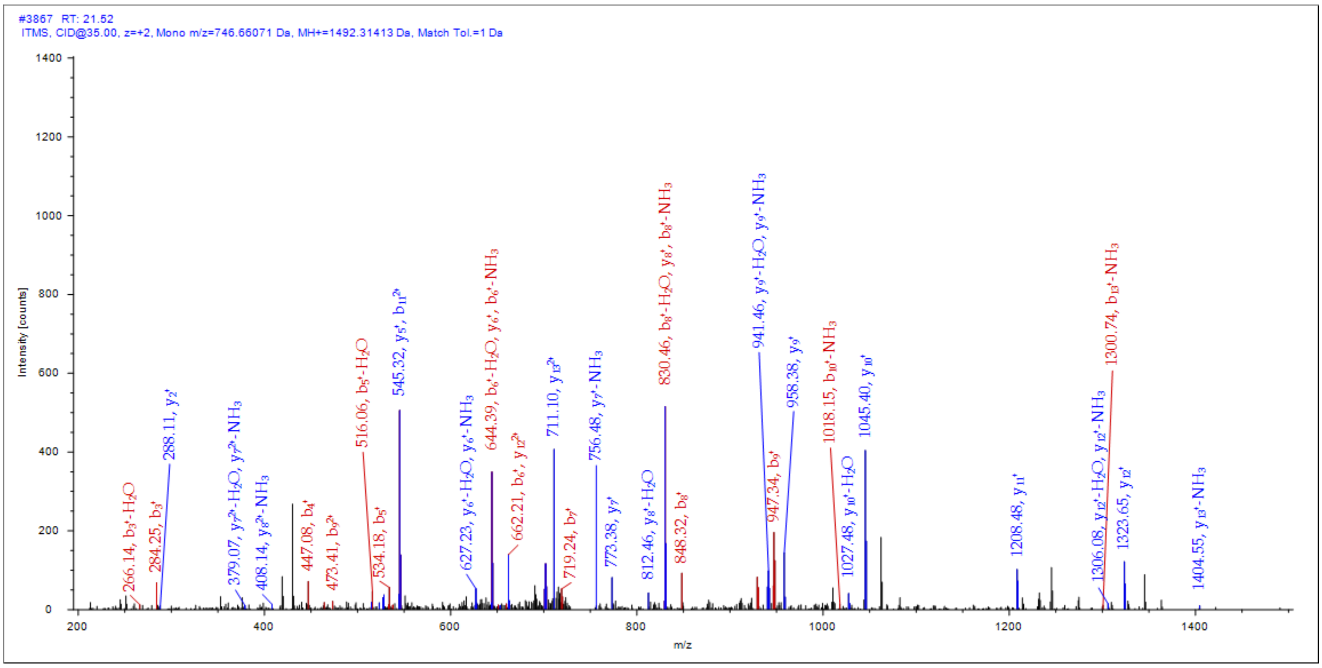

Transcript name (Phytozome *C. reinhardtii* database (Vs. 5.3.1)) or chloroplast (Cp) genome database	Function and/or homologies	Methylated peptide	z	Xcorr	x-times found
Cre07.g349700.t1.2	Similar to 3-beta hydroxysteroid dehydrogenase / isomerase	ALVRDVSKmATSGSGLLAGVGSTTE VVR[b]	3	3.93	2

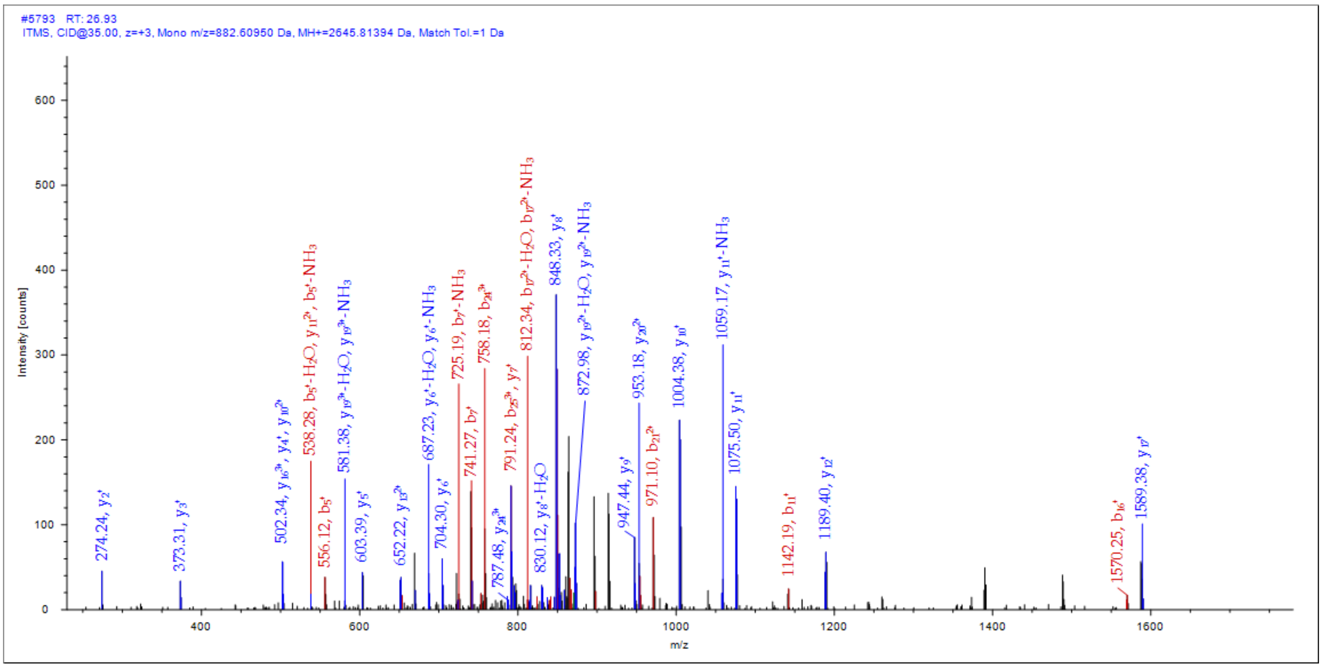

Transcript name (Phytozome *C. reinhardtii* database (Vs. 5.3.1)) or chloroplast (Cp) genome database	Function and/or homologies	Methylated peptide	z	Xcorr	x-times found
Cre07.g349700.t1.2	Similar to 3-beta hydroxysteroid dehydrogenase / isomerase	->ALVRmDVSKATSGSGLLAGVGSTTEV VR[b]	3	3.70	1

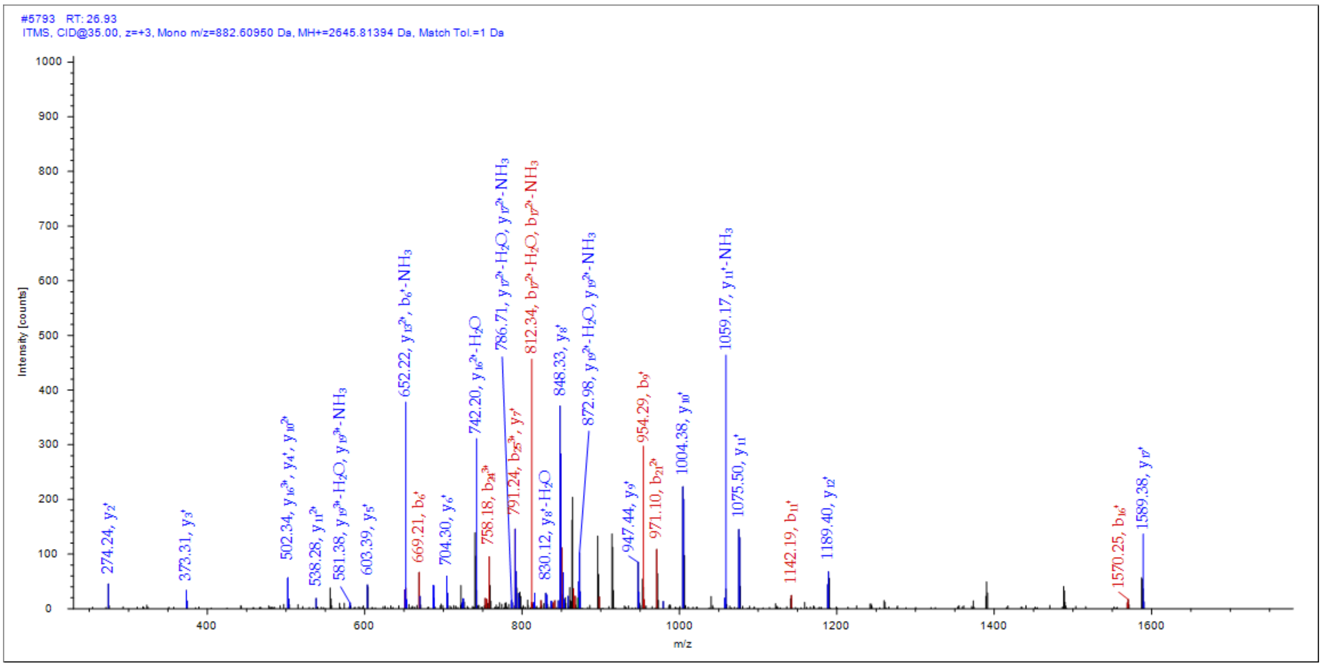

Transcript name (Phytozome *C. reinhardtii* database (Vs. 5.3.1)) or chloroplast (Cp) genome database	Function and/or homologies	Methylated peptide	z	Xcorr	x-times found
Cre01.g017100.t1.3	Similar to proteins with a acylglycerol/acyl-transferase domain	WFESFGAVKASPMAAFRm^{2}LLR[a]	3	3.87	1

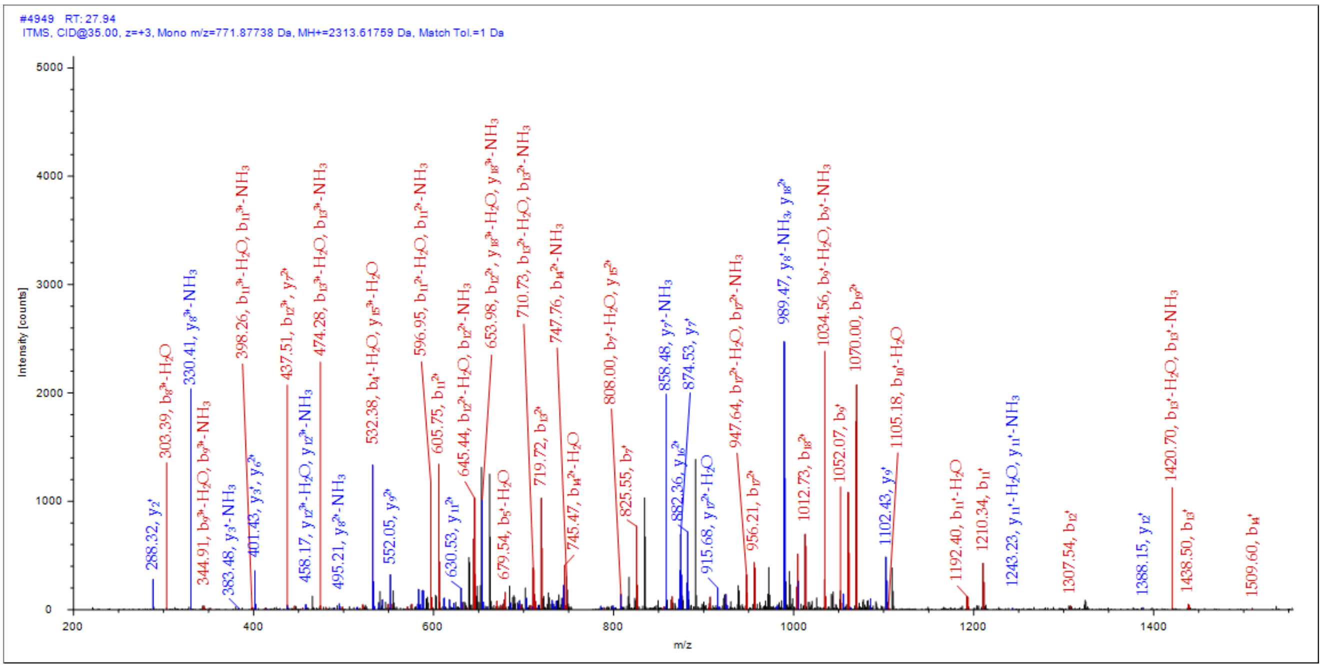

Proteins of unknown function

Transcript name	Function and/or homologies	Methylated peptide	z	Xcorr	x-times found
Cre06.g263250.t1.3	No significant hit in NCBI BLASTp	AAVADATGAASSAAADAKm^2 [a]	2	5.71	7

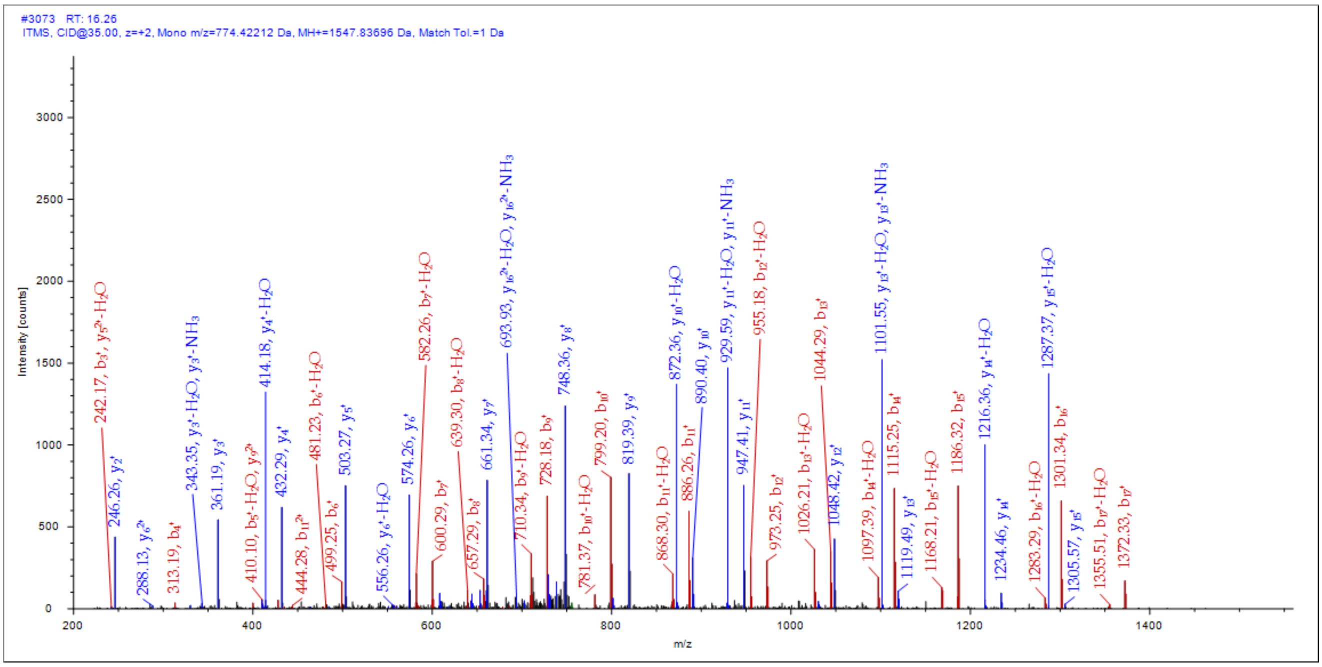

Transcript name (Phytozome *C. reinhardtii* database (Vs. 5.3.1)) or chloroplast (Cp) genome database	Function and/or homologies	Methylated peptide	z	Xcorr	x-times found
Cre06.g263250.t1.3	No significant hit in NCBI BLASTp	AAVADATGAASSAATDAKm[2 a]	2	4.20	3

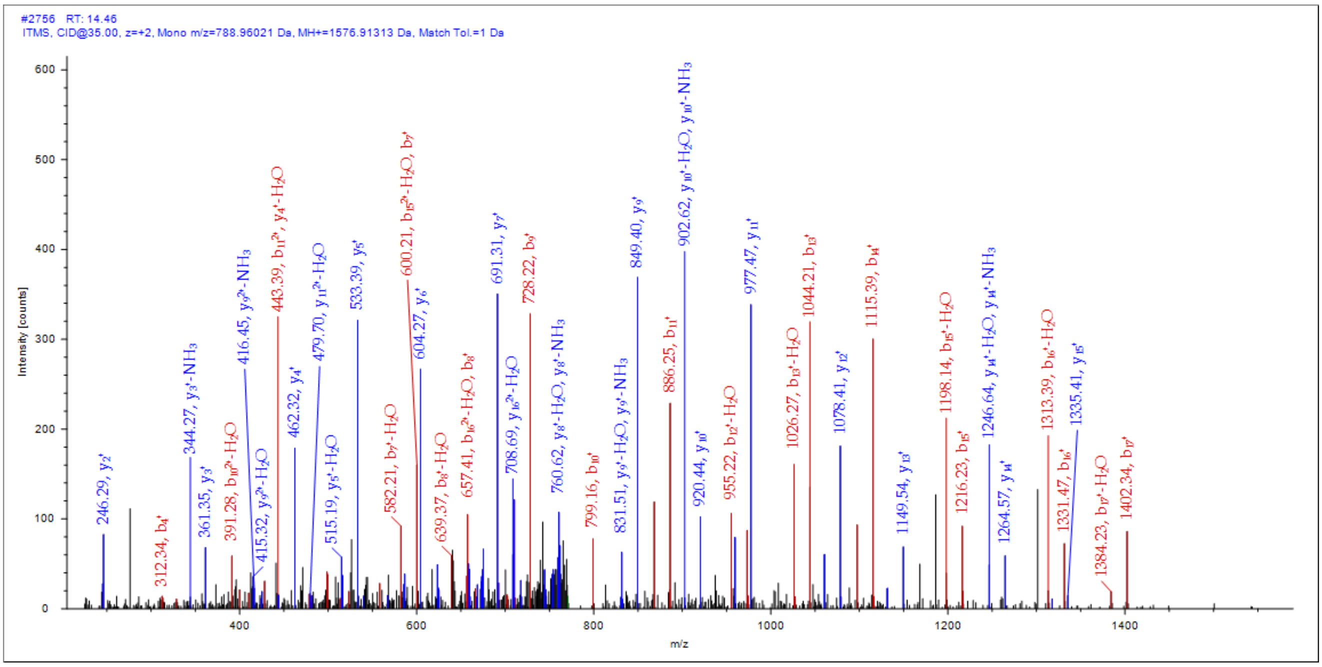

Cp genome	ORF1995 unknown protein	MALEDLSKm[3]WKm[3 a,b]	2	2.71	5

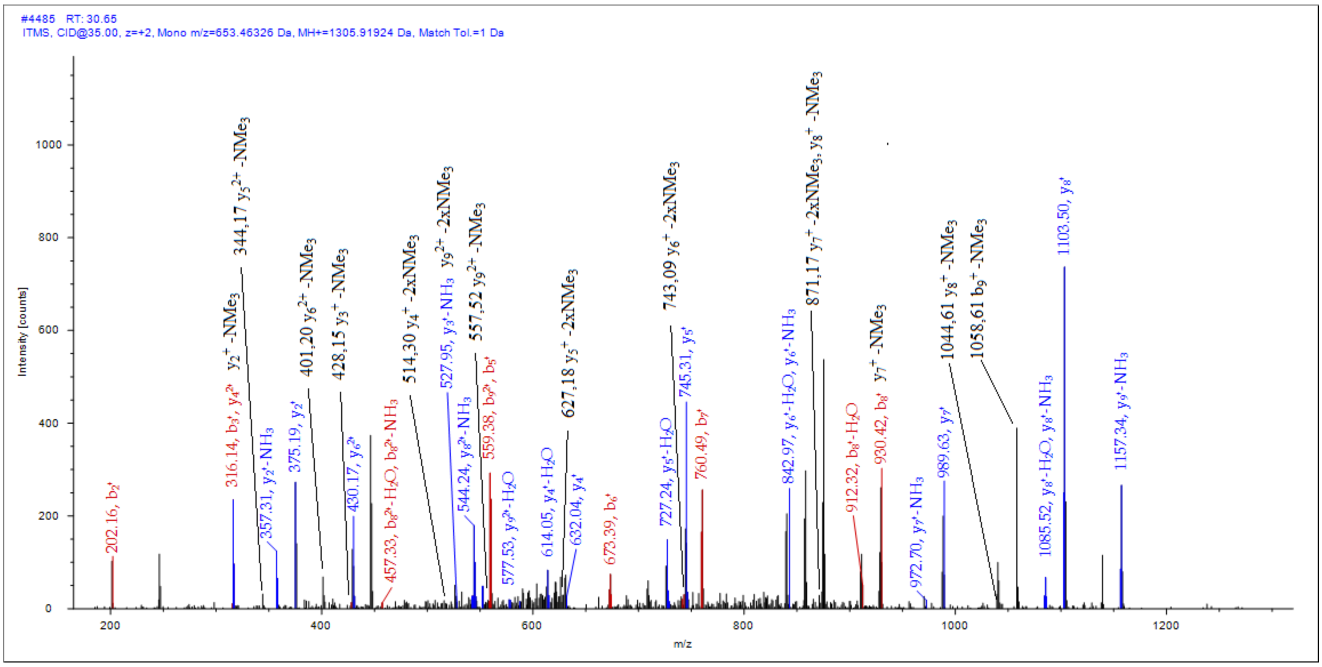

Transcript name (Phytozome *C. reinhardtii* database (Vs. 5.3.1)) or chloroplast (Cp) genome database	Function and/or homologies	Methylated peptide	z	Xcorr	x-times found
Cp genome	ORF1995 unknown protein	SFDITSMTTTLPFYAGWDESLKm[2 a]	2	4.61	3

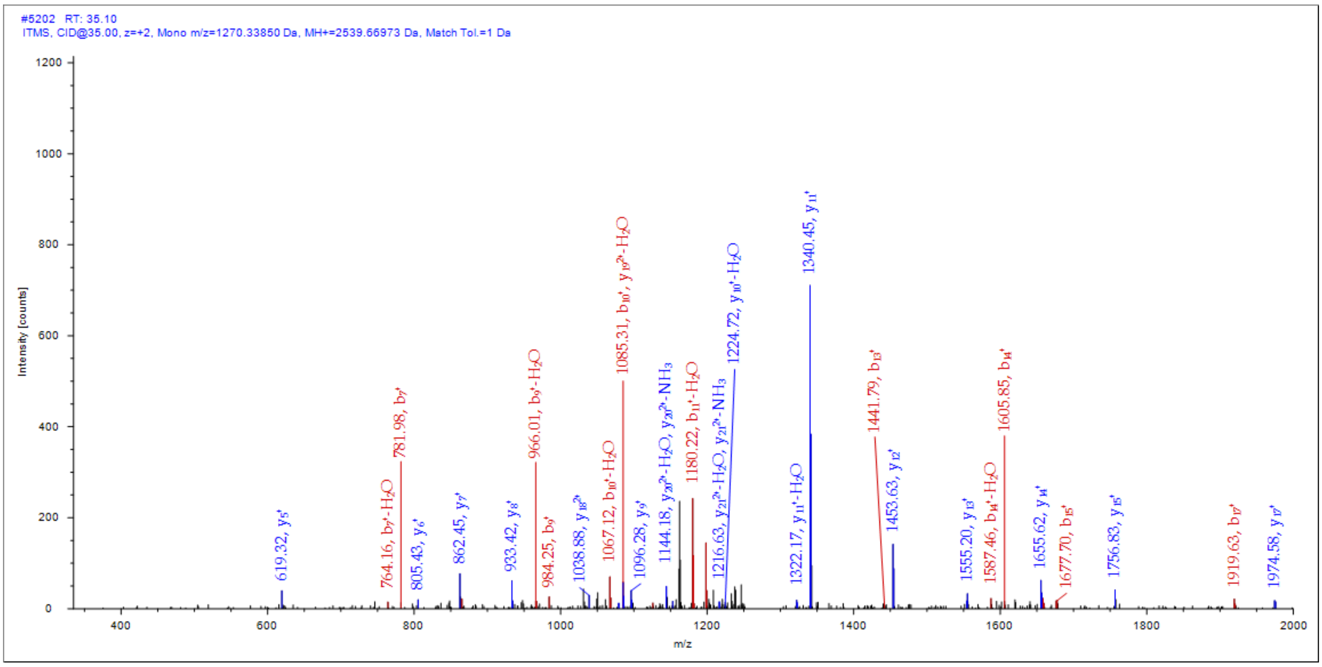

Cp genome	ORF1995 unknown protein	->SFDITSMoTTTLPFYAGWDESLKm[2 a]	2	3.59	1

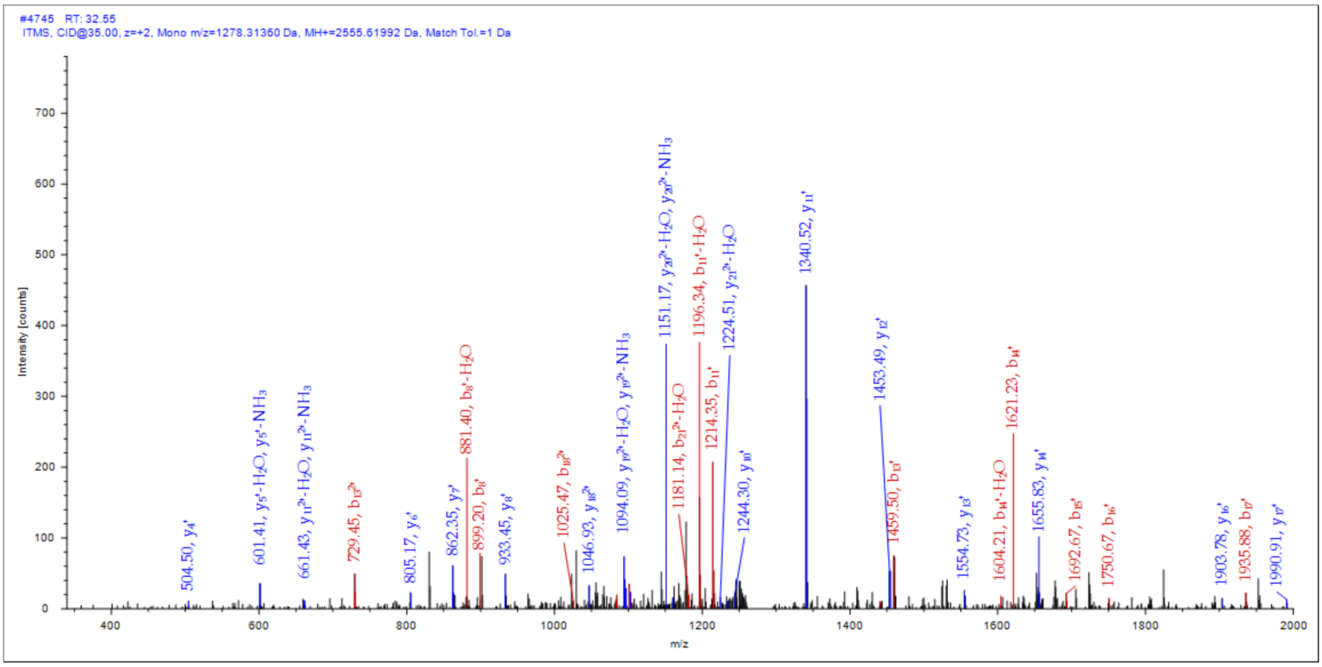

Transcript name (Phytozome *C. reinhardtii* database (Vs. 5.3.1)) or chloroplast (Cp) genome database	Function and/or homologies	Methylated peptide	z	Xcorr	x-times found
Cre01.g000900.t1.2	Similar to conserved plant/cyanobacterial proteins of unknown functions, contains 2 DUF1350 domains	LATVAGQLGVSAATAPLEELSRm[a,b]	2	4.03	4

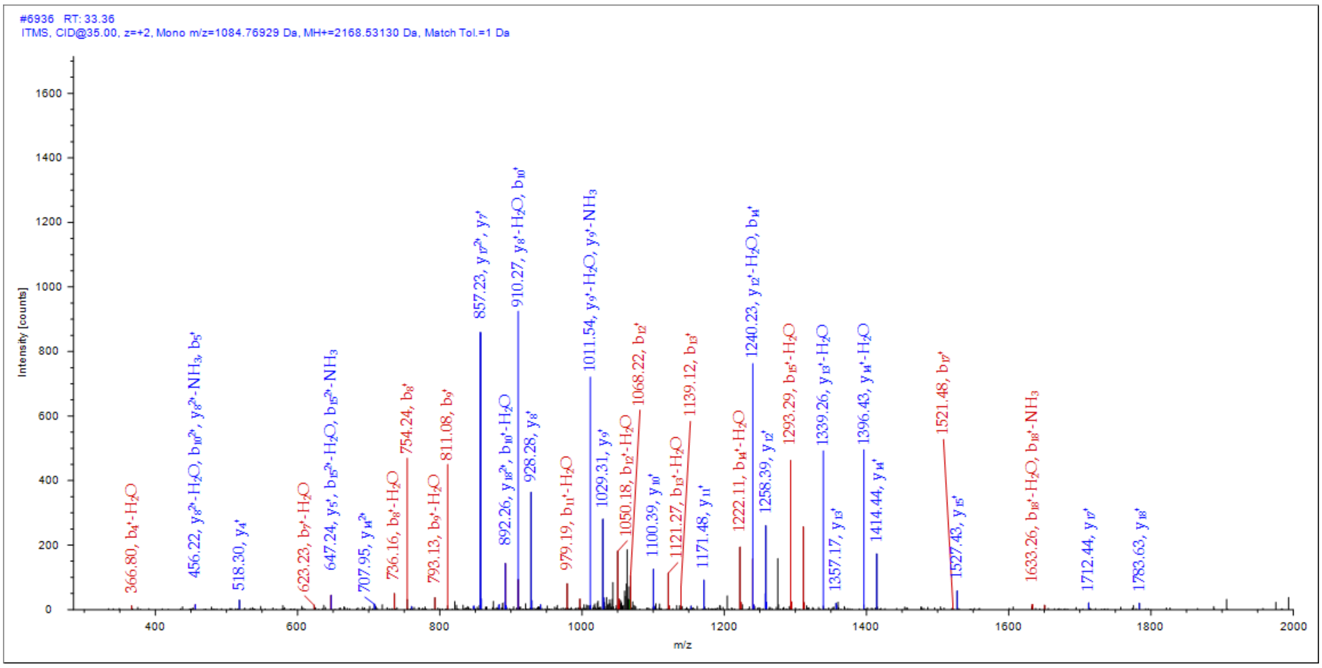

Cre01.g000900.t1.2	Similar to conserved plant/cyanobacterial proteins of unknown functions, contains 2 DUF1350 domains	FKDDSLDDTNNLVQLLQGSSSVGEVLDL TVRm[b]	3	4.35	1

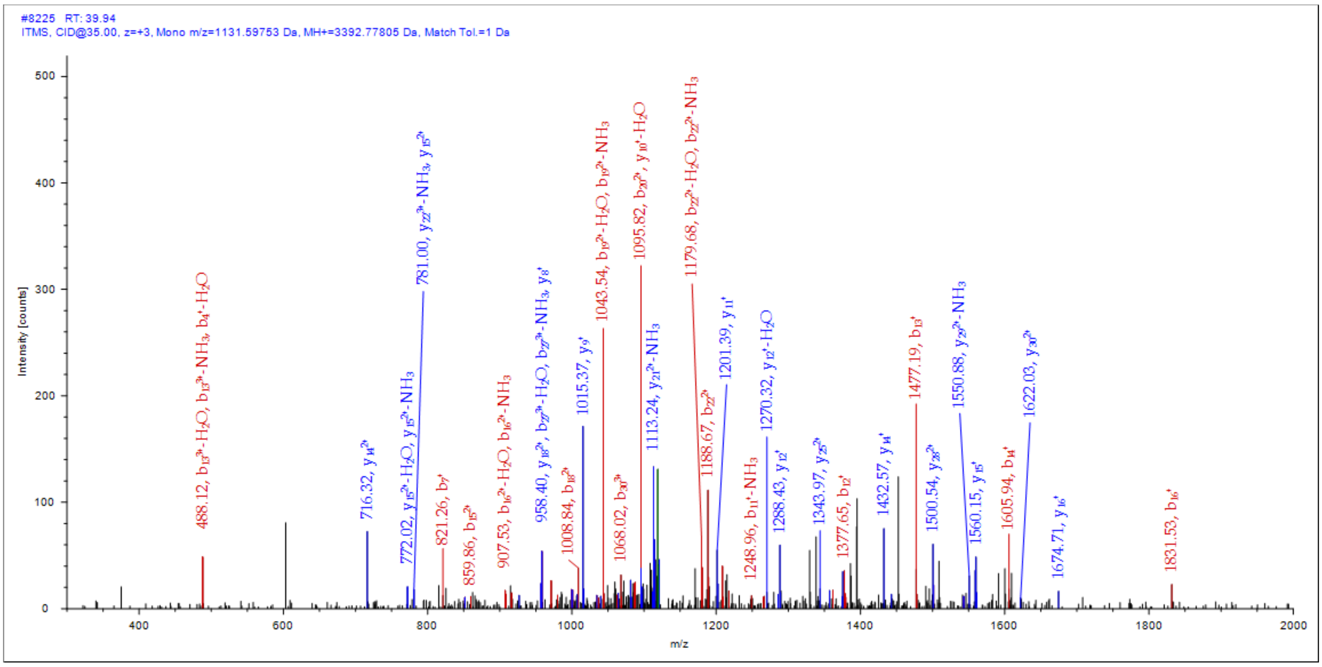

Transcript name (Phytozome *C. reinhardtii* database (Vs. 5.3.1)) or chloroplast (Cp) genome database	Function and/or homologies	Methylated peptide	z	Xcorr	x-times found
Cp genome	ORF2971 unknown protein	VAMoLAELSLSNLSAKm^{3}LDMITDLLVIIDSVRm[a]	3	3.31	1

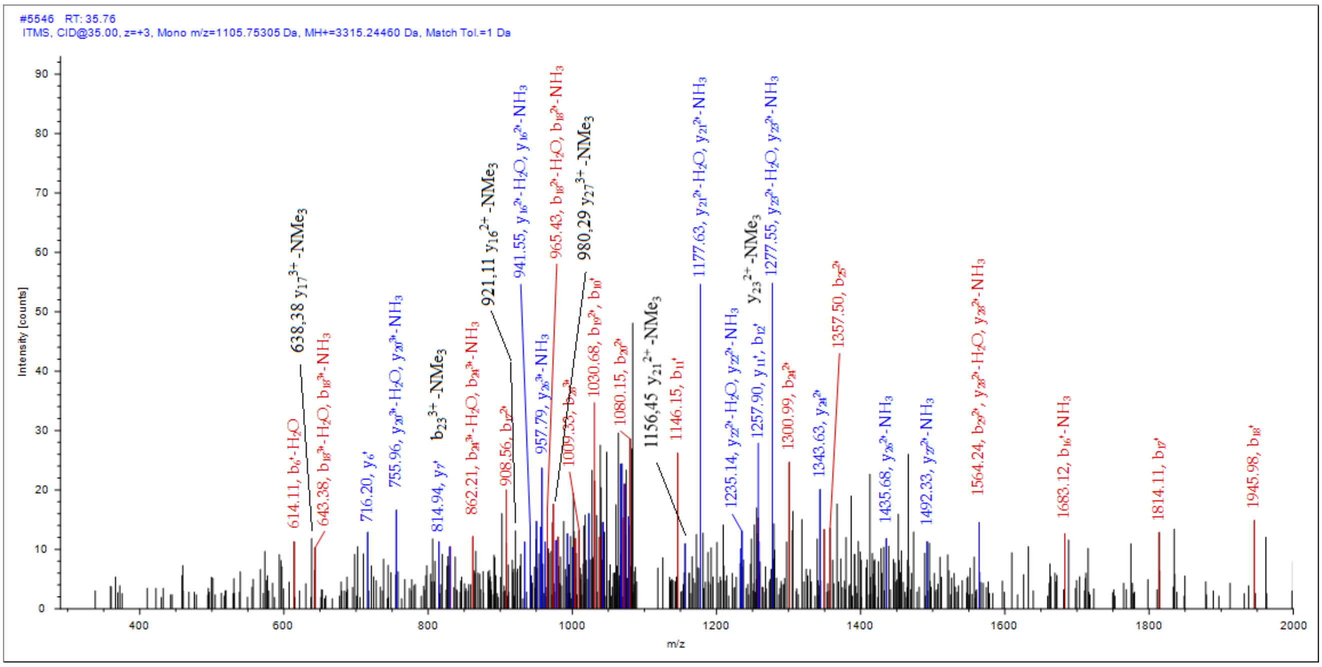

Cp genome	ORF2971 unknown protein	MoGQRmKmSQITLLEKm[a]	2	2.52	1

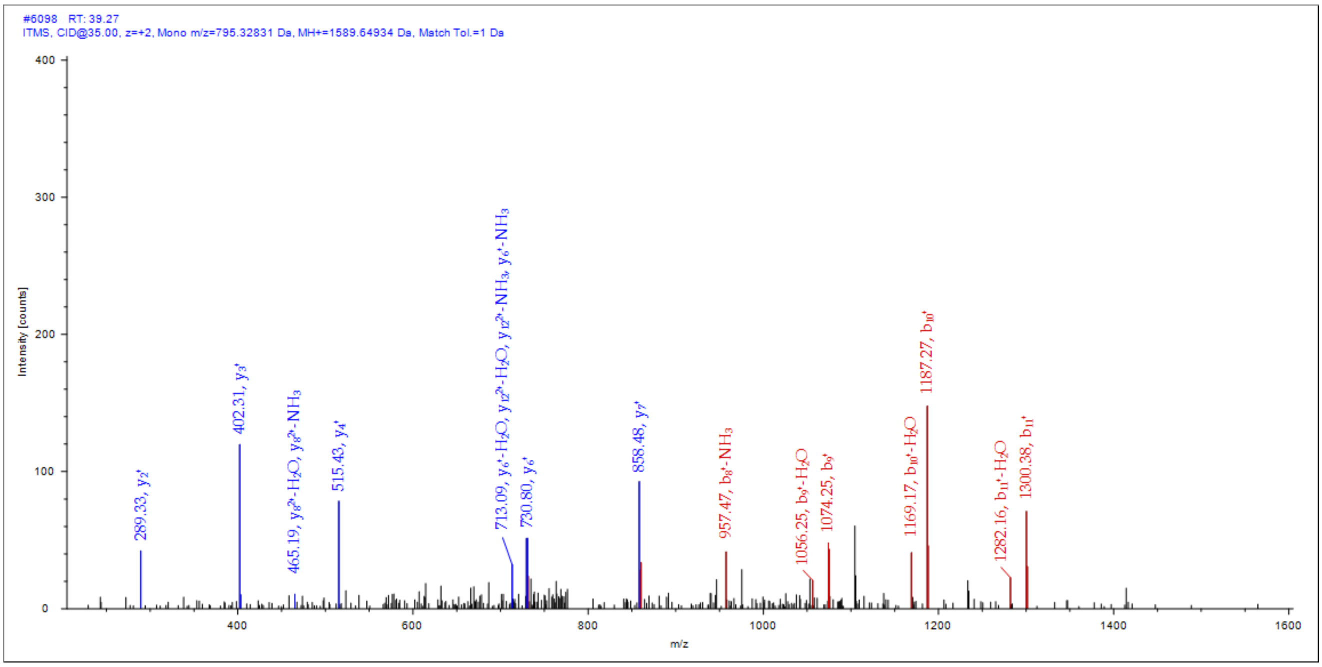

Transcript name (Phytozome *C. reinhardtii* database (Vs. 5.3.1)) or chloroplast (Cp) genome database	Function and/or homologies	Methylated peptide	z	Xcorr	x-times found
g2947.t1	No significant hit in NCBI BLASTp	ADGAAATATTAATGVLGAGFAKmADEA<br>AASATTAATGVLGAGFAKm^{2} [a]	3	3.67	1

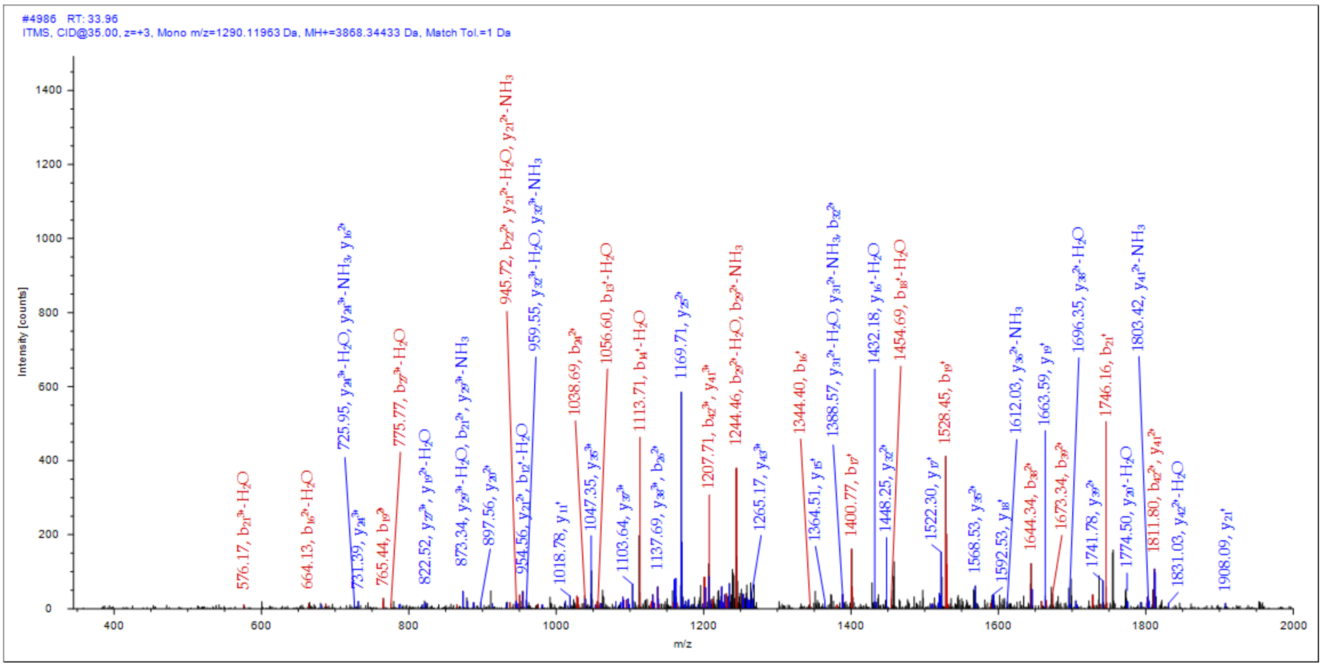

g14174.t1	No significant hit in NCBI BLASTp	GLGDVVGMKm^{3}GPAAEINNGR[a]	2	3.33	1

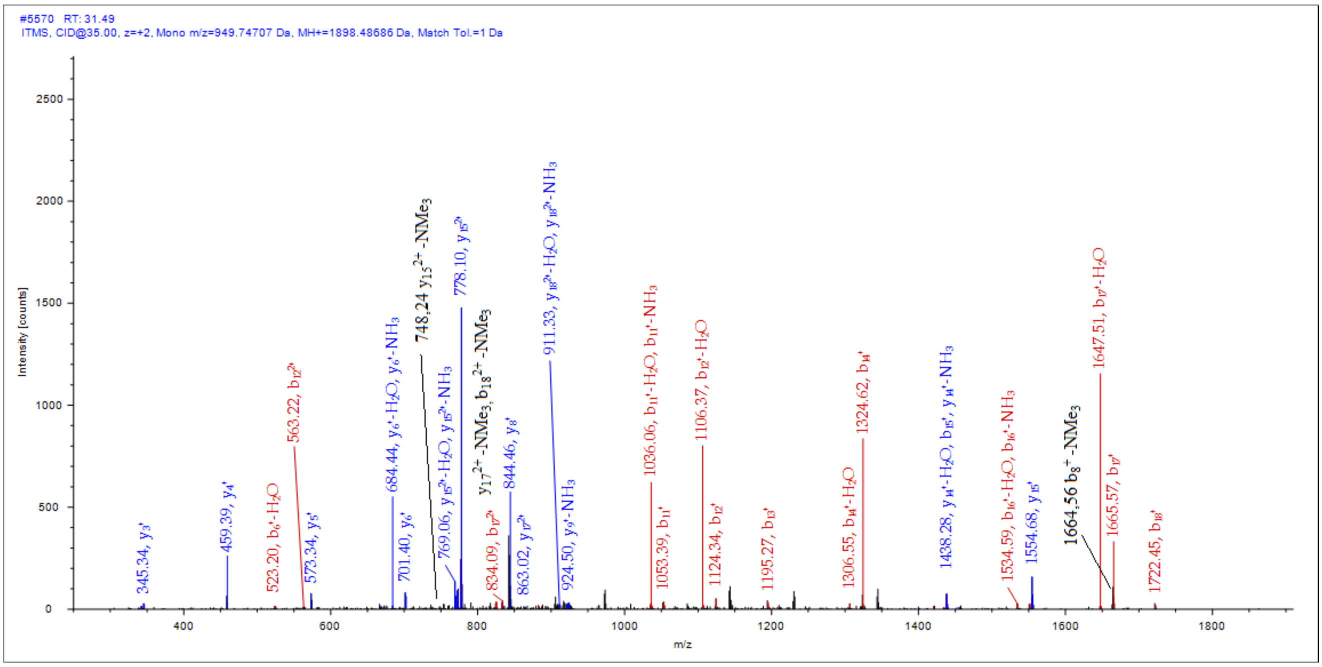

Transcript name (Phytozome *C. reinhardtii* database (Vs. 5.3.1)) or chloroplast (Cp) genome database	Function and/or homologies	Methylated peptide	z	Xcorr	x-times found
Cre10.g438450.t1.3	No significant hit in NCBI BLASTp	GWGKm^{2}LPDSGAALPAFLYKmHVLKm[a]	2	3.13	1

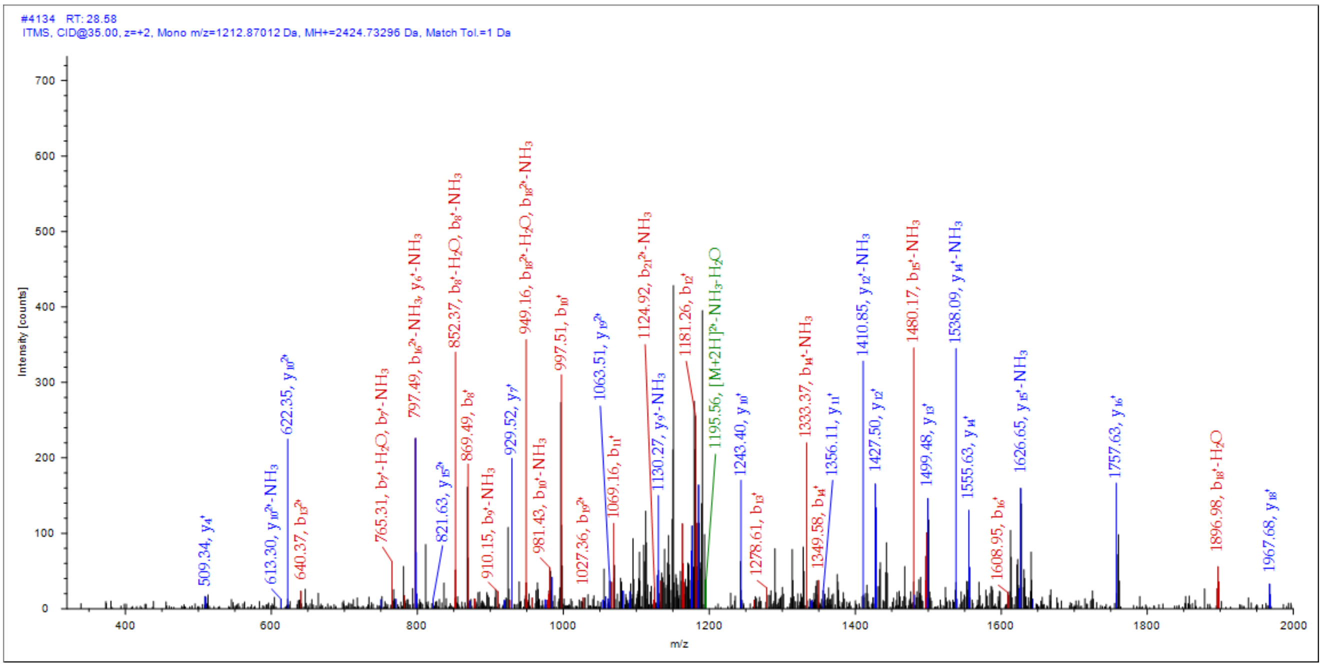

Supplemental Figure S2. Spectra of methylated peptides listed in Tables 2 and 3 along with their identified b and y ions.

All peptides were evaluated by the Proteome Discoverer software vs. 1.4 (Thermo Fisher Scientific) and the assignment of the methylations sites was in addition inspected manually. For each peptide, we considered the scan with the highest Xcorr, showing its corresponding MS/MS spectrum. The matching b (red) and y ions (blue) as well as the precursor ions that lost ammonia and/or water (green) are automatically labeled by the Proteome Discoverer software vs. 1.4. The trimethylamine neutral loss events originating by peptides bearing a trimethylated Lys were in addition manually labeled in black (-NMe_3). All m/z peaks with an intensity of 10 or more were taken into account. [a] Peptide was identified in an analysis for methylated proteins from the extended eyespot fraction $F2A_e$. [b] Peptide was identified in an analysis for methylated proteins from the published eyespot proteome [Schmidt et al., 2006]. Km, methylated Lys; Km^2; dimethylated Lys, Km^3, trimethylated Lys; Rm, methylated Arg; Rm^2, dimethylated Arg; Mo, oxidized Met; z, charge of the precursor ion; Xcorr, cross correlation factor according to Eng et al. (J. Am. Soc. Mass Spectrom. 5:976–989, 1994); Function and/or homologies of depicted proteins were determined by NCBI BLASTp.
